# Organocatalytic Synthesis of Amides Using Thioacids and Anilines via Electron Donor–Acceptor Photoactivation

**DOI:** 10.1021/acs.joc.6c00035

**Published:** 2026-03-30

**Authors:** Malibongwe P. Shandu, Andile R. Ngwenya, Jairus L. Lamola, Paseka T. Moshapo

**Affiliations:** † Research Centre for Synthesis and Catalysis, Department of Chemical Sciences, 61799 University of Johannesburg, Cnr Kingsway Avenue and University Road, P.O. Box 524, Auckland Park, Johannesburg 2006, South Africa; ‡ Research and Technology (R&T) Sasol (Pty) Ltd, 1 Klasie Havenga Road, Sasolburg 1947, South Africa

## Abstract

Amides are important structures with relevance in biology, material science, and synthetic chemistry. Their photochemical synthesis via aminolysis of acetyldisulfides relies on a reductive regime of thioacids using transition metal-based photocatalysts and organic dyes. Here, we report that readily available tetrachlorophthalimide can serve as an organocatalytic acceptor to thioacids for the electron donor–acceptor (EDA) photoactivation, thereby generating the desired thioacyl radicals suitable for dimerization and, subsequently, amidation via aminolysis. The usefulness of this EDA platform enabled us to develop and demonstrate a mechanistically distinct synthesis of sterically and electronically diverse amides using thioacids as acyl sources, with isolated yields of up to 97%. Furthermore, the reactions proved to be efficient and easy to operate as they could be conducted under an open-air atmosphere, eliminating the need to create a rigorous inert atmosphere. Notably, the gram-scale synthesis of paracetamol was achieved using the developed photochemical transformation.

## Graphical abstract

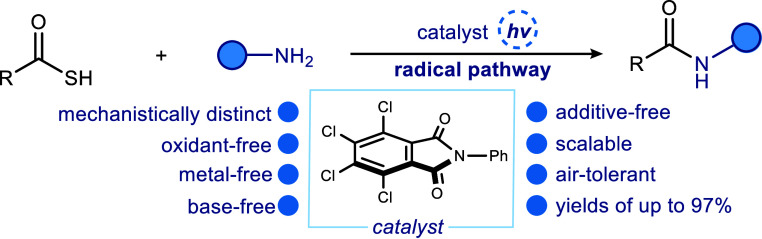

## Introduction

Amides play a crucial role in medicinal chemistry, agrochemicals, and the textile industry.^1–3^ Moreover, they serve as valuable synthetic precursors that can be transformed into other important functional groups. For example, they can be hydrolyzed into carboxylic acids,^4,5^ dehydrated into nitriles,^6,7^ and reduced into amines.^8–10^ Furthermore, the amide functional group is present in the top 20 of the 2024 best-selling pharmaceutical agents.^11^ Therefore, the continuous development of efficient, easy-to-operate, and environmentally benign synthetic strategies for their synthesis remains desirable. The classical methods for amide synthesis rely on the condensation reaction between carboxylic acids and various amines (Figure 1a).^12–14^ However, stoichiometric amounts of activators such as DCC and HATU are required,^15^ leading to excessive waste generation.^16^ Although advancements have been made in this field, other amide bond synthesis techniques require strictly anhydrous conditions, transition metal catalysts, high reaction temperatures, and external CO sources.^17–22^

**Figure 1:**
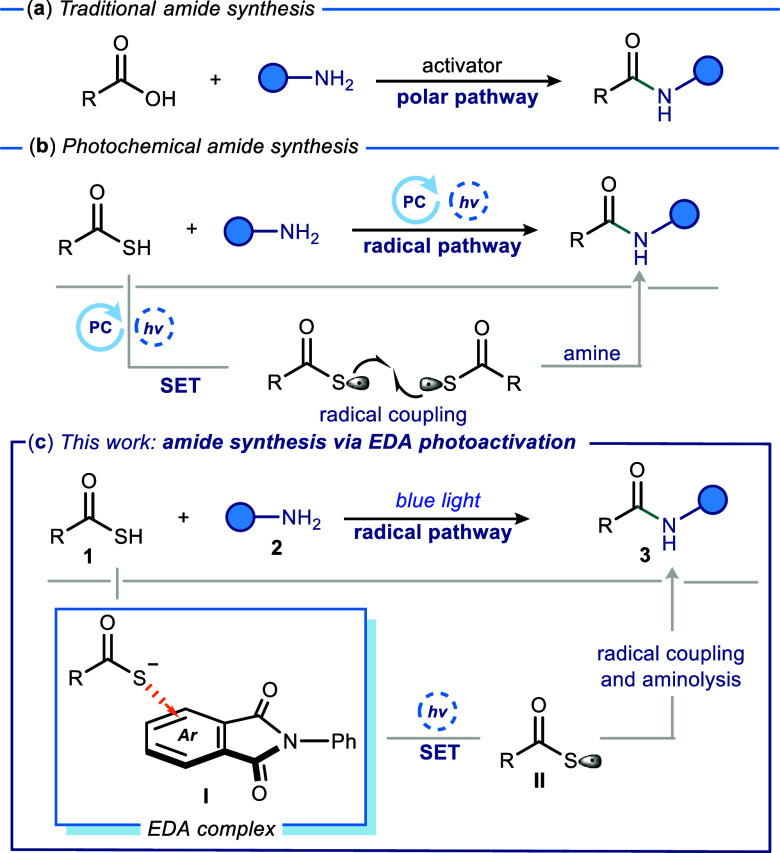
(**a**) Traditional methods for the synthesis of amides. (**b**) Photochemical methods for the synthesis of amides using thioacids. (**c**) Synthesis of amides using thioacids via EDA photoactivation.

Thioacids have been demonstrated to be efficient acyl sources for the synthesis of amide functional groups.^23,24^ Although the same reactivity can be achieved via polar pathways, photochemical techniques (Figure 1b) eliminate the necessity of additives such as HOBt and isocyanides and elevated reaction temperatures and longer reaction times.^25–30^ For example, photochemical amide synthesis using thioacids and amines has been achieved using transition metal-based photocatalysts (PC) such as Ru­(bpy)_3_Cl_2_ and CdS nanoparticles.^26–28^ Furthermore, the catalytic amount of the organic dye Mes-Acr-MeBF_4_ rendered this transformation effective, successfully forming an amide moiety with various amino acids.^25^ These photochemical techniques follow a similar catalytic cycle. Generally, the photoactive photocatalyst undergoes a single-electron transfer (SET) process, thereby generating the thioacyl radical. Following the radical–radical addition, the resulting disulfide undergoes aminolysis with an amine, thus generating the desired amide (Figure 1b).

Although these seminal works present unprecedented applicability of thioacids in photochemical amide synthesis, the EDA photoactivation pathway remains, to the best of our knowledge, unexplored. Motivated by the use of tetrachlorophthalide-based substrates as organocatalysts suitable for EDA complex formation with various donors for C–C bond formation,^31,32^ we surmised that they would be suitable acceptors for the deprotonated thioacids. Therefore, we present a mechanistically distinct base and additive-free radical generation photochemical technique for amide synthesis. We hypothesized that EDA photoactivation **I** between the thioacids **1** and tetrachlorophthalide-based organocatalyst would generate radical **II**. Thereafter, upon radical–radical recombination, aminolysis with aniline **2** would generate desired amide **3** (Figure 1c). The reports that demonstrated thioester synthesis using thioacids and their salt variations as donors for EDA photoactivation further supported the feasibility of our design plan.^33–36^

## Results and Discussion

We began our investigations with thioacetic acid **1a** and *p*-toluidine **2a** in acetonitrile (MeCN) for 6 h (Table 1). Irradiating the reaction mixture with blue LEDs furnished product **3a** in good to excellent yields using catalysts **A** (*E* ^red^ = −1.78 V vs Ag/AgCl (3.5 M KCl))^31^ and **B** (*E* ^red^ = −0.84 V vs Ag/AgCl (3.5 M KCl))^31^ (75–90%, entries 1 and 2). Maintaining catalyst **B**, with dichloroethane (DCE) solvent, resulted in a 79% product yield (entry 3). Evaluating other common organic solvents resulted in the formation of product **3a** in moderate yields (37–48%, entries 4–6). Lowering the catalyst loading resulted in an excellent yield, albeit with a low efficiency (84%, entry 8). Moreover, the addition of K_2_CO_3_ did not improve the yield (90%, entry 7). Reducing the reaction time and the thioacetic acid **1a** equivalent also resulted in slightly diminished product yields of **3a** (65–77%, entries 9 and 10). Control experiments demonstrated that light and the catalyst were essential for this transformation (entries 11 and 12).

**Table 1:**
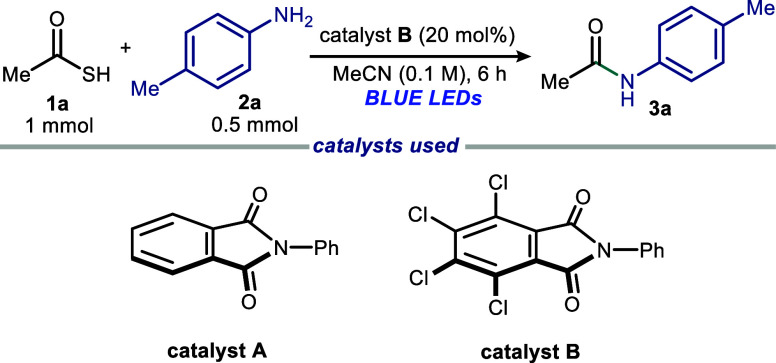
Optimization and Control Experiments^a^

entry	variations	yield (%)^b^
1	none	90
2	catalyst **A**	75
3	DCE	79
4	DMF	42
5	DMSO	37
6	DMA	48
7	K_2_CO_3_	90
8	10 mol % catalyst **B**	84
9	3 h	77
10	0.5 mmol **1a**	65
11	no light	trace
12	no catalyst	trace

a Reactions performed on a 0.5 mmol scale for 6 h using 2 equiv of **1a** and 1 equiv of **2a** under illumination by a blue LED strip (460–465 nm).

b Reported yields are isolated yields of **3a**.

We next performed experiments to gain mechanistic insights. The formation of the EDA complex in the ground state was suggested through UV–vis spectroscopic analysis (Figure 2).^37^ Upon mixing **1a**, **2a**, and catalyst **B**, a marked bright-yellow color resulted (see Supporting Information), and a new charge-transfer absorption band in the visible region (purple line), consistent with the formation of a ground-state EDA complex, was observed. Furthermore, upon mixing **1a** with catalyst **B** and K_2_CO_3_, a bright-orange color and a new charge-transfer band were observed (see the Supporting Information). This alluded to potassium thioacetate participation in the ground-state EDA formation with catalyst **B**.

**Figure 2:**
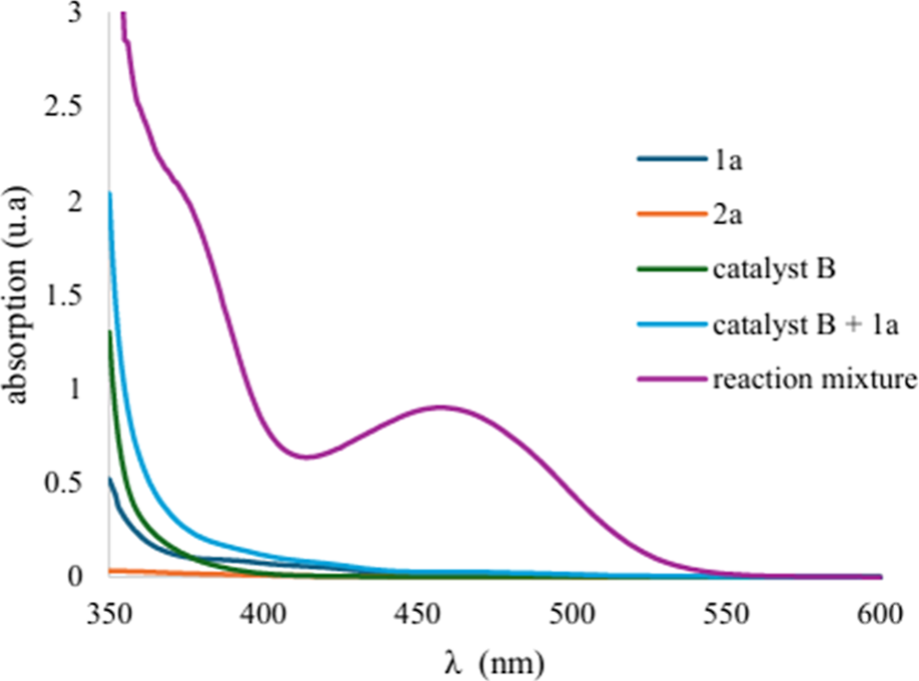
Optical absorption spectra, recorded in MeCN in 1 mm path quartz cuvettes of the separate reaction components **1a**, **2a**, and catalyst **B**, and appearance of the colored EDA complex between **1a**, **2a**, and catalyst **B**. [**1a**] = 0.2 M, [**2a**] = 0.1 M, and [catalyst **B**] = 0.02 M.

With the established reaction conditions at hand (Table 1, entry 1), we then evaluated the compatibility of thioacetic acid **1a** with various aniline derivatives of **2** (Figure 3). It is worth noting that, similar to other reported methods,^25,26,28,31^ this photochemical transformation required no additional stoichiometric amounts of bases and oxidants. *p*-Substituted anilines such as 4-bromo **2b**, 4-chloro **2c**, 4-methylthio **2d**, and 4-trifluoromethylthio **2e** reacted efficiently, furnishing products **3b**–**3e** in almost quantitative yields (85–92%). Furthermore, anilines substituted with heterocyclic moieties, **2f** and **2g**, known to possess biological relevance,^38–41^ were well-tolerated, furnishing the corresponding products **3f** and **3g** in excellent yields of 91% and 82%, respectively. The unsubstituted **2h** and polycyclic **2i** anilines also reacted efficiently, offering amide products **3h** and **3i** in excellent yields (83–97%). However, although *m*-substituted aniline **2j** was well-tolerated (**3j**, 78%), other sterically hindered anilines, **2k** and **2l**, remained completely unreactive, possibly due to steric hindrance. Furthermore, *N*-methylaniline **2m** and *N*-benzylamine **2n** were also unreactive, although *p*-methoxy-substituted benzylamine **2o** resulted in product **3o** in 69% yield.

**Figure 3:**
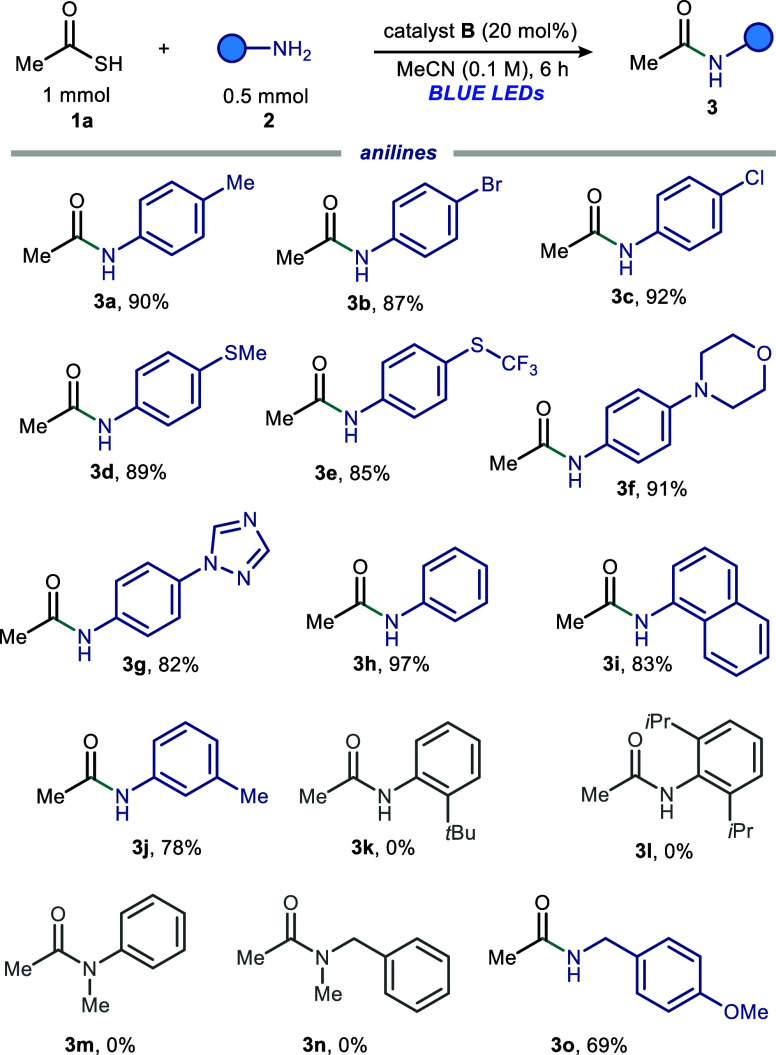
Evaluation of electronically and sterically diverse aniline **2** derivatives for amide synthesis, performed on a 0.5 mmol scale using 2 equiv of **1a**. Reported yields are the isolated yields of amide products **3**.

We then evaluated the ability of tetrachlorophthalimide-based catalyst **B** to undergo EDA photoactivation with electronically and sterically diverse thioacids **1** (Figure 4). Thiobenzoic acid **1p** reacted efficiently to produce product **3p** in 81% yield. Coupling of *o*-substituted thiobenzoic acids **1q** and **1r** proceeded efficiently to give the corresponding amide products **3q** and **3r** in good yields (70–78%). Cinnamic thioacid **1s** was also well-tolerated (**3s**, 84%). Straight-chain alkyl thioacids **1t** and **1u**, as well as branched **1v**, provided amide products **3t**–**3v** in 70–81% yields, respectively. Photochemical coupling of 4-chlorophenylthioacetic acid **1w**, 4-methylphenylthioacetic acid **1x**, 3-bromophenylthioacetic acid **1y**, and polycyclic-based 1-naphthalenethioacetic acid **1z** resulted in amide products **3w**–**3z** with good to excellent yields of 78–83%. Moreover, cycloalkyl thioacid **1aa** afforded product **3aa** in a good yield of 71%. Lastly, the heterocyclic indole-based thioacid **1ab** reacted efficiently (**3ab**, 86%), demonstrating excellent regioselectivity.

**Figure 4:**
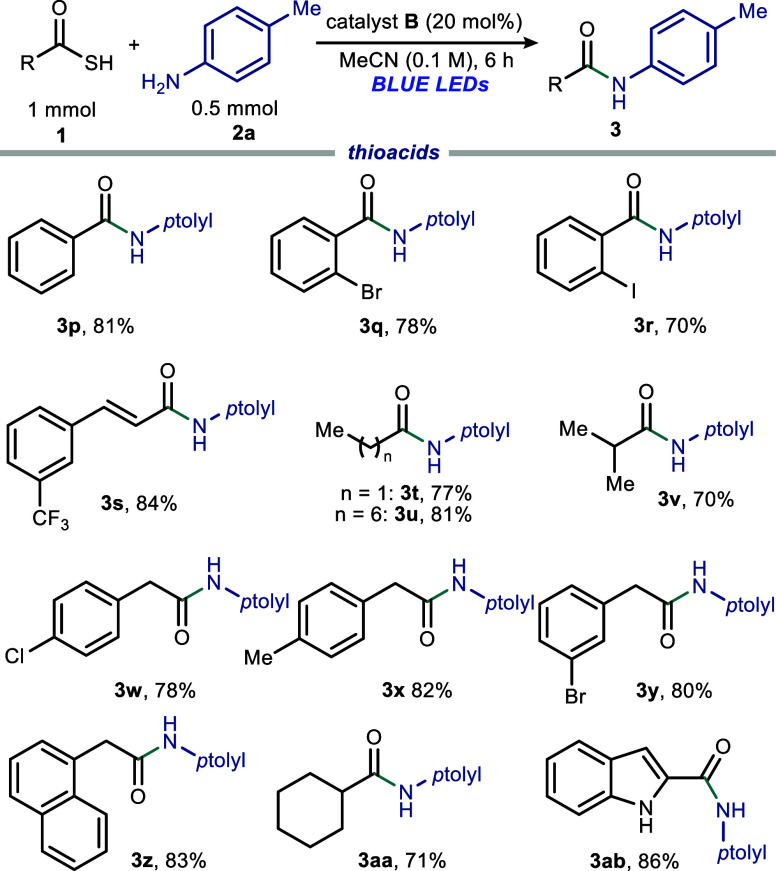
Evaluation of electronically and sterically diverse thioacid **1** derivatives for amide synthesis performed on a 0.5 mmol scale using 2 equiv of **1**. Reported yields are the isolated yields of amide products **3**.

We next examined the synthetic utility of the developed method on a scale-up reaction basis (Scheme 1a). Using the model reaction on a 10 mmol scale, product **3a** and paracetamol **3ac** were obtained in yields of 67% and 69%, respectively. Finally, we carried out reactions to elucidate the reaction mechanism (Scheme 1b). When the model reaction was conducted in the presence of TEMPO, a well-established radical trap, only a trace of product **3a** was observed, suggesting a radical pathway for this developed protocol. Furthermore, in the presence of argon, a lowered yield of product **3a** was obtained (37%), and using a stoichiometric amount of **B** under an argon atmosphere afforded product **3a** in 88% yield. This indicated the possible importance of atmospheric oxygen in catalyst regeneration. Additionally, the exclusion of *p*-toluidine **2a** under the established reaction conditions resulted in the formation acyl disulfide **4a** with 62% yield. Subsequently, we performed an experiment using the preformed acyl disulfide **4a** in the dark, furnishing amide product **3a** with 76%. This suggests that the aminolysis of **4a** with anilines proceeds via a polar pathway. Lastly, to investigate the interaction between thioacetic acid **1a** and *p*-toluidine **2a** prior to the formation of EDA complex **I**, ^1^H NMR titration experiments were conducted in CDCl_3_ at room temperature (see the Supporting Information). Incremental addition of *p*-toluidine **2a** to thioacetic acid **1a** resulted in the systematic disappearance of the thioacetic acid **1a** acidic proton and the emergence of proton signals attributed to the *p*-toluidinium species, demonstrating the favorable deprotonation of thioacetic acid **1a** by *p*-toluidine **2a**.

**Scheme 1:**
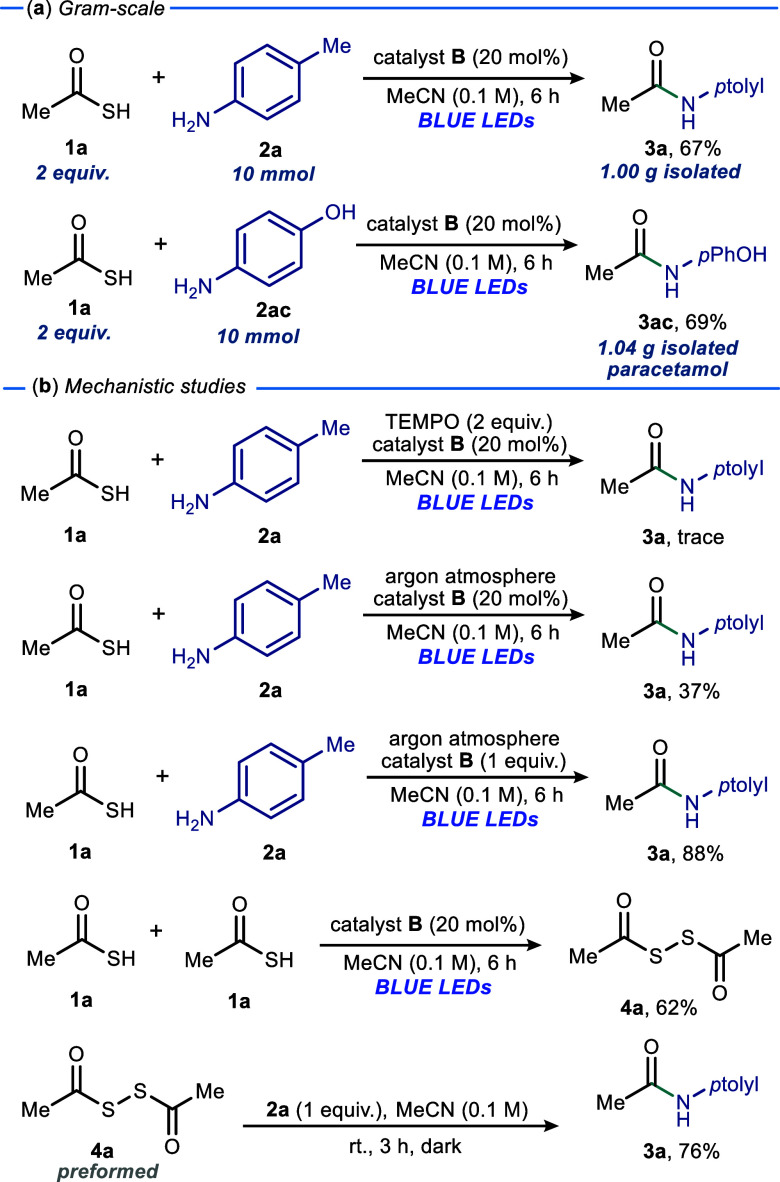
Gram-Scale Synthesis and Mechanistic Evaluation for Amide Bond Formation

Figure 5 illustrates the proposed reaction mechanism for the developed protocol based on the findings demonstrated in Scheme 1b and the ^1^H NMR titration experiments. Following the deprotonation of thioacid **1** by aniline **2**, resulting electron-rich intermediate **III** forms EDA complex **I** with catalyst **B**. Upon visible-light irradiation, SET results in the generation of radical **II**. This intermediate **II** subsequently undergoes radical coupling to yield intermediate **4**. Aminolysis with aniline **2** then follows, furnishing desired amide product **3** with simultaneous release of intermediate **5**. This intermediate **5** could then undergo aminolysis with aniline **2** to produce amide product **3**.^25^ On the other hand, radical anion **IV** undergoes oxidative turnover likely by molecular oxygen, thus regenerating catalyst **B** and completing the catalytic cycle.

**Figure 5:**
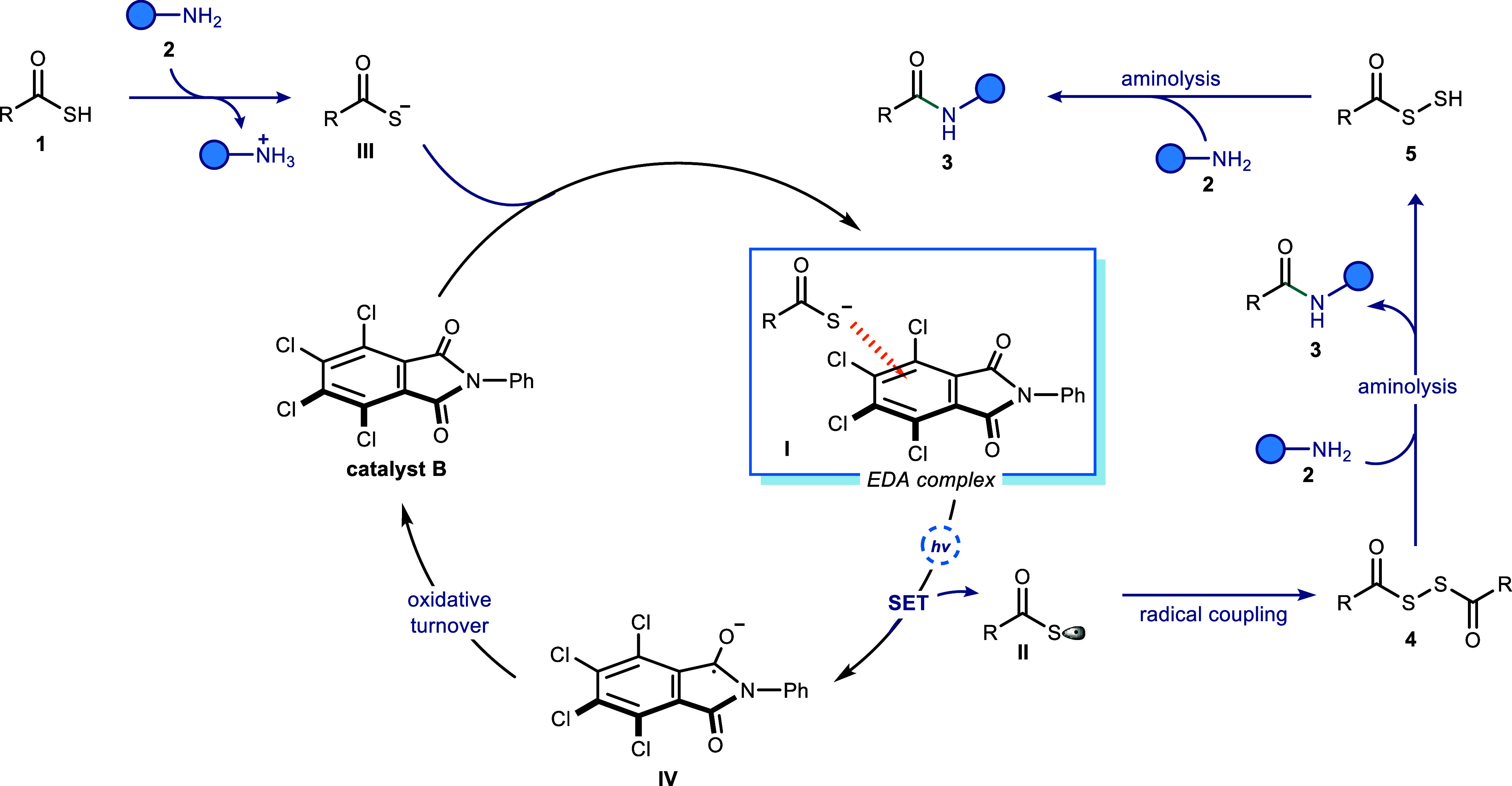
Proposed reaction mechanism for the amide synthesis via EDA photoactivation.

## Conclusions

In summary, we have demonstrated that tetrachlorophthalimide-based catalyst **B** is an effective organocatalyst for the synthesis of electronically and sterically diverse amides **3** via EDA photoactivation. This protocol, which follows a mechanistically distinct amide **3** synthesis using thioacids **1** as carbonyl sources, proved to be efficient, atom-economic, and easy to operate. Good to excellent isolated product yields of up to 97% were obtained, regardless of the structural variations of thioacids **1** and anilines **2**. Furthermore, we disclose the practicality of this protocol through the gram-scale synthesis of paracetamol **3ac**. Given the prevalence of the amide moiety in bioactive molecules and the textiles industry, we anticipate this protocol will find broad use in the synthetic community.

## Supplementary Material



## Data Availability

The data underlying this study are available in the published article and its Supporting Information.

## Notes

Details of experimental procedures, full characterization data, and NMR spectra (PDF)
