# Supplementary material for: Organocatalytic Synthesis of Amides Using Thioacids and Anilines via Electron Donor–Acceptor Photoactivation
Source: J Org Chem. 2026 Mar 30;91(14):5085–90. doi: 10.1021/acs.joc.6c00035 (PMC13077700; doi:10.1021/acs.joc.6c00035)
Supplement: Supplementary file 1 [file jo6c00035_si_001.pdf]

Organocatalytic Synthesis of Amides using Thioacids and Anilines *via* Electron Donor-Acceptor Photoactivation

Malibongwe P. Shandu,[a] Andile R. Ngwenya,[a] Jairus. L. Lamola,[b] and Paseka T. Moshapo*[a]

[a]Research Centre for Synthesis and Catalysis. Department of Chemical Sciences, University of Johannesburg, Cnr Kingsway Avenue and University Road, PO Box 524, Auckland Park, 2006, Johannesburg, South Africa.

[b]Research and Technology (R&T) Sasol (Pty) Ltd 1 Klasie Havenga Road, Sasolburg, 1947, South Africa.

*Correspondence to: pasekam@uj.ac.za

Supporting Information

Table of Contents

1. General Information S3

1.1 Reagent information S3

1.1 General analytical information S3

2. Synthesis of Catalysts S3

3. Synthesis of Thioacids S4

4. Experimental Procedures S4

4.1 Experimental Setup S4

4.2 General Procedure A S5

4.3 General Procedure B S17

4.4 Mechanistic Studies S19

4.4 Reference List S23

4.5 Spectral Data S25

1. General Information

1.1 Reagent information

The solvents and commercially available reagents used were purchased from Sigma Aldrich and used as received. Synthesised substrates were prepared using the reported protocol, and the spectral data were matched to those reported in the literature.

1.1 General analytical information

The reaction crudes were further monitored using thin-layer chromatography (TLC) on aluminium-backed Merck silica gel 60 F254 plates using an ascending technique. The TLC plates were visualised using UV light at 254 nm. The synthesised compounds were purified using gravity column chromatography on Merck silica gel 60 (230-400 mesh) and characterised using NMR spectroscopy, FTIR and melting point. The ^{1}H (7.26 ppm reference) and $^{13}C\{^{1}H\}$ (77.16 ppm reference) were recorded in $CDCl_3$ and the ^{1}H (2.50 ppm and 3.33 ppm reference) and $^{13}C\{^{1}H\}$ (39.52 ppm reference) were recorded in DMSO-d_6 solutions using a 500 MHz magnet coupled to an Avance III HD 500 MHz Console and reported in ppm. NMR data is reported as follows: chemical shift (δ ppm), multiplicity (s = singlet, d = doublet, t = triplet, q = quartet, m = multiplet, dd = doublet of doublets, ddd = doublet of doublet of doublets, coupling constant (Hz), and integration. To improve the solubility of amides in $CDCl_3$, a few drops of DMSO-d_6 were added. For mass analysis, a liquid chromatography-quadrupole time-of-flight tandem mass spectrometer (LC-MS-9030 QTOF-MS instrument, Shimadzu Corporation, Kyoto, Japan). For IR stretching frequencies, SHIMADZU IR Affinity-1s in cm^{-1} was used. The melting points were obtained using a Stuart SMP10 thermobaric hot-stage equipped with a microscope.

2. Synthesis of Catalysts

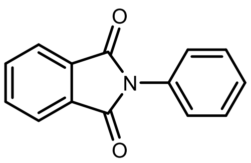

2-Phenylisoindoline-1,3-dione: To an oven-dried Schlenk tube equipped with a stirrer was added phthalic anhydride (2.96 g, 20 mmol), aniline (2.17 mL, 24 mmol, 1.2 equiv.) and glacial acetic acid (40 mL). The reaction mixture was refluxed at 120 °C using an oil bath, and after 3 h, the solution was concentrated under *vacuo*. The resulting solids on cooling were collected by filtration and sequentially washed with 10% sodium carbonate (3 × 100 mL), water (3 × 100 mL), and methanol (3 × 100 mL). The resulting white solids were dried under *vacuo* to afford catalyst A (81%, 3.78 g). Characterisation data were consistent with the literature values.[1]

^{1}H NMR (500 MHz, $CDCl_3$) δ 7.98 – 7.91 (m, 2H), 7.79 (d, *J* = 3.1 Hz, 2H), 7.51 (t, *J* = 7.5 Hz, 2H), 7.47 – 7.37 (m, 3H).

^{13}C{^{1}H} NMR (126 MHz, $CDCl_3$) δ 167.3, 134.4, 131.8, 129.2, 128.1, 126.6, 123.8.

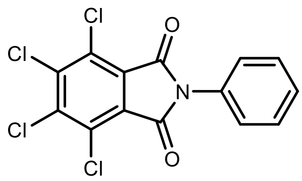

4,5,6,7-tetrachloro-2-phenylisoindoline-1,3-dione: To an oven-dried Schlenk tube equipped with a stirrer was added tetrachlorophthalic anhydride (5.72 g, 20 mmol), aniline (2.17 mL, 24 mmol, 1.2 equiv.) and glacial acetic acid (40 mL). The reaction mixture was refluxed at 120 °C using an oil bath, and after 3 h, the solution was concentrated under *vacuo*. The resulting solids on cooling were collected by filtration and sequentially washed with 10% sodium carbonate (3 × 100 mL), water (3 × 100 mL), and methanol (3 × 100 mL). The resulting white solids were dried under *vacuo* to afford catalyst B (76%, 5.48 g). Characterisation data were consistent with the literature values.[2]

^{1}H NMR (500 MHz, $CDCl_3$) δ 7.52 (t, J = 7.7 Hz, 2H), 7.43 (m, 3H).

^{13}C{^{1}H} NMR (126 MHz, $CDCl_3$) δ 162.6, 140.7, 131.0, 130.3, 129.4, 128.9, 127.4, 126.6.

3. Synthesis of Thioacids

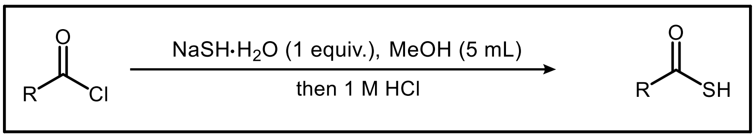

To an oven-dried round-bottom flask equipped with a stirrer was added sodium hydrosulfide monohydrate (0.31 g, 4 mmol), acid chloride (2 mmol) and MeOH (10 mL) at 0 °C using an ice bath. After 1 h, the reaction mixture was allowed to warm to room temperature and stirred for an additional 2 h. Water was then added, and the mixture was acidified with 1 M HCl (20 mL). The resulting mixture was extracted using EtOAc (3 × 20 mL). The combined organic layers were dried over anhydrous sodium sulphate and concentrated under reduced pressure, and used without any further purification.[3]

Acid chlorides were synthesised by dissolving carboxylic acids (5 mmol) in anhydrous DCM (10 mL) and DMF (catalytic amounts = 4 drops) at 0 °C. Oxalyl chloride (1.5 equiv.) was then added dropwise, and the reaction was allowed to warm to room temperature. After 4 h, the reaction mixture was concentrated under reduced pressure and used without any further purification.[4]

4. Experimental Procedures

4.1 Experimental Setup

Setup 1: The reactions were performed using a blue LED strip as a light source wrapped inside a recrystallising dish (Figure S1) using a 20 mL borosilicate reaction vial was used and the reactions were carried out in air. A fan was used to cool down the reactions (measured temperature after stopping the reactions = 22 – 26 °C). The 10 W, USB-powered 2 m strip (460 – 465 nm) was purchased from QingXi Technology Co., Ltd.

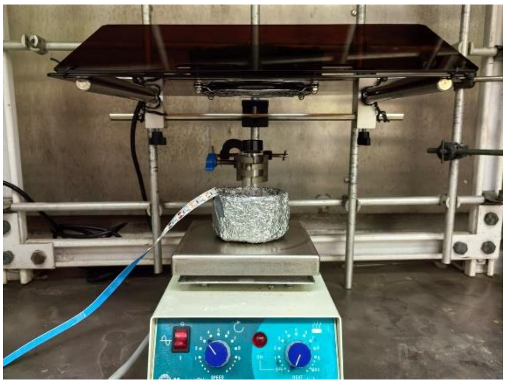

Figure S1. Blue LED photoreactor used in the amidation reactions

Setup 2: The scale reactions were performed using a blue LED light source (Figure S2). A fan was used to cool down the reactions (measured temperature after stopping the reaction = 24 °C). The 19.7 W, USB-powered light (λmax = 460 nm) was purchased from WOLEZEK LED. The 100 mL round-bottom flasks were placed 2 – 3 cm away from the light source, and the reactions were carried out in air.

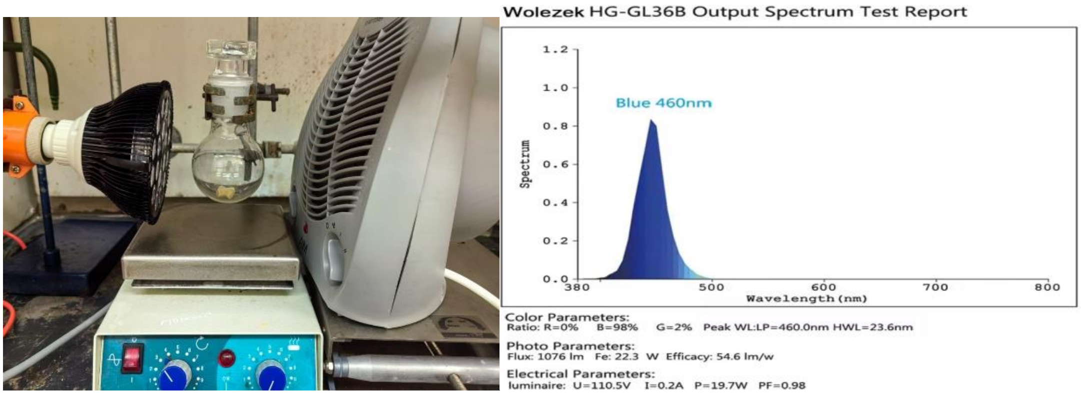

Figure S2. Blue LED photoreactor system used in the scale-up amidation reactions, and the emission spectrum of the LED used in this setup

4.2 General Procedure A

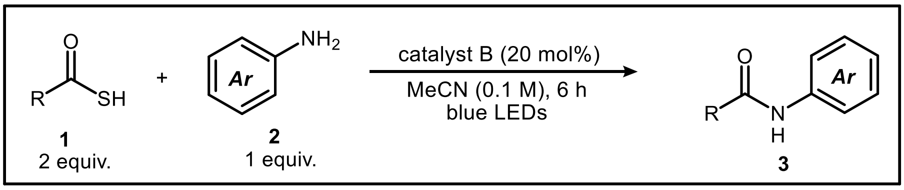

To a 20 ml reaction vial was added aniline **2** (0.5 mmol, 1 equiv.), catalyst B (0.036 g, 20 mol%, 0.2 equiv.), MeCN (5 mL, 0.1 M) and thioacid **1** (1 mmol, 2 equiv.). The reaction vial was capped and irradiated using setup 1. After 6 h, the reaction mixture was quenched with brine (10 mL) and extracted with EtOAc (3 × 20 mL). The combined organic layers were dried under anhydrous Na_2SO_4,

concentrated under reduced pressure and purified *via* gravity column chromatography on silica gel using 15% EtOAc in *n*-hexanes to afford the desired products **3**.

Characterisation Data for Products Generated from General Procedure A

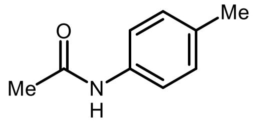

***N*-(*p*-tolyl)acetamide 3a**: According to General Procedure A, *p*-toluidine (0.05 g, 0.5 mmol, 1 equiv.), catalyst B (0.036 g, 20 mol%, 0.2 equiv.), thioacetic acid (0.07 mL, 1 mmol, 2 equiv.), and MeCN (5 mL, 0.1 M). After 6 h, the reaction was subjected to a workup process outlined in General Procedure A and purified *via* gravity column chromatography using EtOAc/hexane = 15:85 (v/v) as the eluent to afford the title product as a white solid (0.067 g, 90%). Characterisation data were consistent with the literature values.[3]

^{1}H NMR (500 MHz, $CDCl_3$) δ 7.72 (s, 1H), 7.37 (d, J = 8.0 Hz, 2H), 7.09 (d, J = 7.9 Hz, 2H), 2.30 (s, 3H), 2.13 (s, 3H).

^{13}C{^{1}H} NMR (126 MHz, $CDCl_3$) δ 168.7, 135.5, 133.9, 129.5, 120.3, 24.4, 20.9.

IR (film): ν_{max} 3287, 3256, 3186, 3109, 3048, 1667, 1589, 1528, 1481, 1389, 1304, 1250, 1003, 818 cm^{-1}.

Melting point: 146 – 148 °C.

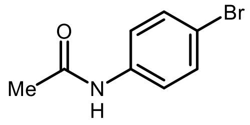

***N*-(*p*-bromophenyl)acetamide 3b**: According to General Procedure A, 4-bromoaniline (0.09 g, 0.5 mmol, 1 equiv.), catalyst B (0.036 g, 20 mol%, 0.2 equiv.), thioacetic acid (0.07 mL, 1 mmol, 2 equiv.), and MeCN (5 mL, 0.1 M). After 6 h, the reaction was subjected to a workup process outlined in General Procedure A and purified *via* gravity column chromatography using EtOAc/hexane = 15:85 (v/v) as the eluent to afford the title product as a white solid (0.093 g, 87%). Characterisation data were consistent with the literature values.[5]

^{1}H NMR (500 MHz, DMSO-d_6) δ 9.61 (s, 1H), 7.49 (d, J = 6.1 Hz, 2H), 7.34 (d, J = 8.7 Hz, 2H), 2.08 (s, 3H).

^{13}C{^{1}H} NMR (126 MHz, DMSO-d_6) δ 168.4, 137.8, 130.9, 120.8, 115.0, 23.6.

IR (film): ν_{max} 3287, 3256, 3186, 3109, 3048, 1667, 1589, 1528, 1481, 1389, 1304, 1250, 1003, 818 cm^{-1}.

Melting point: 172 – 173 °C.

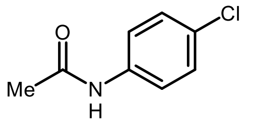

***N*-(*p*-chlorophenyl)acetamide (3c)**: According to General Procedure A, 4-chloroaniline (0.06 g, 0.5 mmol, 1 equiv.), catalyst B (0.036 g, 20 mol%, 0.2 equiv.), thioacetic acid (0.07 mL, 1 mmol, 2 equiv.), and MeCN (5 mL, 0.1 M). After 6 h, the reaction was subjected to a workup process outlined in General Procedure A and purified *via* gravity column chromatography using EtOAc/hexane = 15:85 (v/v) as the eluent afford the title product as a white solid (0.078 g, 92%). Characterisation data were consistent with the literature values.[3]

^{1}H NMR (500 MHz, DMSO-d$_6$) δ 9.64 (s, 1H), 7.32 (d, J = 7.5 Hz, 2H), 6.96 (d, J = 7.6 Hz, 2H), 1.83 (s, 3H).

^{13}C{^{1}H} NMR (126 MHz, DMSO-d$_6$) δ 168.7, 137.7, 128.1, 127.2, 120.6, 23.8.

IR (film): ν_{max} 3194, 3125, 1659, 1597, 1535, 1481, 1366, 1312, 1258, 1173, 1088, 1011, 964, 826 cm^{-1}.

Melting point: 178 – 179 °C.

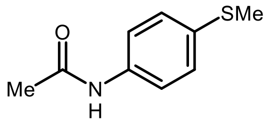

***N*-(*p*-(methylthio)phenyl)acetamide (3d)**: According to General Procedure A, 4-methylthioaniline (0.06 mL, 0.5 mmol, 1 equiv.), catalyst B (0.036 g, 20 mol%, 0.2 equiv.), thioacetic acid (0.07 mL, 1 mmol, 2 equiv.), and MeCN (5 mL, 0.1 M). After 6 h, the reaction was subjected to a workup process outlined in General Procedure A and purified *via* gravity column chromatography using EtOAc/hexane = 15:85 (v/v) as the eluent to afford the title product as a white solid (0.080 g, 89%). Characterisation data were consistent with the literature values.[6]

^{1}H NMR (500 MHz, DMSO-d$_6$) δ 9.69 (s, 1H), 7.67 (d, J = 8.1 Hz, 2H), 7.31 (d, J = 8.2 Hz, 2H), 2.57 (s, 3H), 2.24 (s, 3H).

^{13}C{^{1}H} NMR (126 MHz, DMSO-d$_6$) δ 168.2, 136.4, 131.6, 127.2, 119.8, 23.6, 16.0.

IR (film): ν_{max} 3271, 3102, 2916, 1643, 1589, 1528, 1489, 1389, 1366, 1312, 1258, 1096, 1042, 1011, 957, 818 cm^{-1}.

Melting point: 130 – 133 °C.

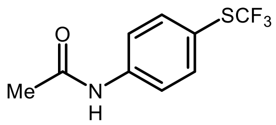

***N*-(*p*-((trifluoromethyl)thio)phenyl)acetamide (3e)**: According to General Procedure A, 4-(trifluoromethyl)aniline (0.08 mL, 0.5 mmol, 1 equiv.), catalyst B (0.036 g, 20 mol%, 0.2 equiv.), thioacetic acid (0.07 mL, 1 mmol,

2 equiv.), and MeCN (5 mL, 0.1 M). After 6 h, the reaction was subjected to a workup process outlined in General Procedure A and purified *via* gravity column chromatography using EtOAc/hexane = 15:85 (v/v) as the eluent to afford the title product as a white solid (0.099 g, 85%). Characterisation data were consistent with the literature values.[7]

^{1}H NMR (500 MHz, DMSO-d_6) δ 10.21 (s, 1H), 7.71 (d, *J* = 8.4 Hz, 2H), 7.60 (d, *J* = 8.4 Hz, 2H), 2.05 (s, 3H).

^{13}C{^{1}H} NMR (126 MHz, DMSO-d_6) δ 168.8, 142.2, 137.1, 129.55 (q, *J* = 308.0 Hz, 1C), 119.7, 115.5, 24.0.

IR (film): ν_{max} 3186, 3109, 1667, 1589, 1528, 1489, 1396, 1373, 1319, 1265, 1111, 1011, 826 cm^{-1}.

Melting point: 187 – 188 °C.

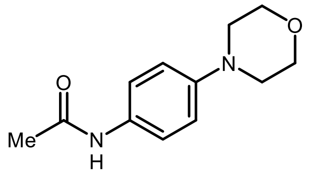

***N*-(*p*-morpholinophenyl)acetamide (3f)**: According to General Procedure A, 4-morpholinoaniline (0.09 g, 0.5 mmol, 1 equiv.), catalyst B (0.036 g, 20 mol%, 0.2 equiv.), thioacetic acid (0.07 mL, 1 mmol, 2 equiv.), and MeCN (5 mL, 0.1 M). After 6 h, the reaction was subjected to a workup process outlined in General Procedure A and purified *via* gravity column chromatography using EtOAc/hexane = 15:85 (v/v) as the eluent to afford the title product as a white solid (0.100 g, 91%). Characterisation data were consistent with the literature values.[8]

^{1}H NMR (500 MHz, DMSO-d_6) δ 9.08 (s, 1H), 7.39 (d, *J* = 8.4 Hz, 2H), 6.76 (d, *J* = 8.4 Hz, 2H), 3.76 (d, *J* = 3.8 Hz, 4H), 3.01 (d, *J* = 3.9 Hz, 4H), 2.02 (s, 3H).

^{13}C{^{1}H} NMR (126 MHz, DMSO-d_6) δ 168.0, 147.1, 131.2, 120.6, 115.4, 66.2, 49.2, 23.4.

IR (film): ν_{max} 3279. 2955, 1651, 1512, 1450, 1412, 1373, 1296, 1111, 1042, 926, 856, 818 cm^{-1}.

Melting point: 218 – 220 °C.

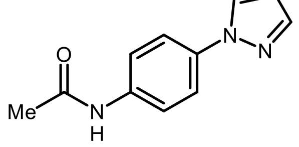

***N*-(*p*-(1*H*-1,2,4-triazol-1-yl)phenyl)acetamide (3g)**: According to General Procedure A, *N*-(4-(1*H*-1,2,4-triazol-1-yl)phenyl)aniline (0.08 g, 0.5 mmol, 1 equiv.), catalyst B (0.036 g, 20 mol%, 0.2 equiv.), thioacetic acid (0.07 mL, 1 mmol, 2 equiv.), and MeCN (5 mL, 0.1 M). After 6 h, the reaction was subjected to a workup process outlined in General Procedure A and purified *via* gravity column chromatography using EtOAc/hexane = 15:85 (v/v) as the eluent to afford the title product as a white solid (0.083 g, 82%). Characterisation data were consistent with the literature values.[9]

^{1}H NMR (500 MHz, DMSO-d$_6$) δ 10.04 (s, 1H), 8.42 (d, *J* = 9.1 Hz, 1H), 7.84 (d, *J* = 6.6 Hz, 3H), 7.76 (d, *J* = 7.9 Hz, 2H), 2.17 (s, 3H).

^{13}C{^{1}H} NMR (126 MHz, DMSO-d$_6$) δ 168.3, 139.2, 133.5, 131.5, 121.9, 120.3, 119.6, 23.7.

IR (film): ν_{max} 3279, 1754, 1651, 1520, 1366, 1219, 1111, 995, 817 cm^{-1}.

Melting point: 152 – 153 °C.

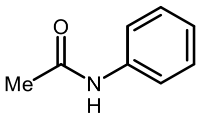

***N*-phenylacetamide (3h)**: According to General Procedure A, aniline (0.05 mL, 0.5 mmol, 1 equiv.), catalyst B (0.036 g, 20 mol%, 0.2 equiv.), thioacetic acid (0.07 mL, 1 mmol, 2 equiv.), and MeCN (5 mL, 0.1 M). After 6 h, the reaction was subjected to a workup process outlined in General Procedure A and purified *via* gravity column chromatography using EtOAc/hexane = 15:85 (v/v) as the eluent to afford the title product as a white solid (0.066 g, 97%). Characterisation data were consistent with the literature values.[6]

^{1}H NMR (500 MHz, CDCl$_3$) δ 8.04 (s, 1H), 7.50 (d, *J* = 7.9 Hz, 2H), 7.28 (t, *J* = 7.7 Hz, 2H), 7.08 (t, *J* = 7.3 Hz, 1H), 2.13 (s, 3H).

^{13}C{^{1}H} NMR (126 MHz, CDCl$_3$) δ 168.9, 138.1, 128.9, 124.3, 120.2, 24.4.

IR (film): ν_{max} 3287, 1659, 1597, 1535, 1489, 1435, 1366, 1391, 1258, 1011, 910 cm^{-1}.

Melting point: 114 – 116 °C.

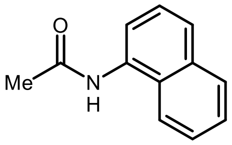

***N*-(naphthalen-1-yl)acetamide (3i)**: According to General Procedure A, 1-naphthylamine (0.08 g, 0.5 mmol, 1 equiv.), catalyst B (0.036 g, 20 mol%, 0.2 equiv.), thioacetic acid (0.07 mL, 1 mmol, 2 equiv.), and MeCN (5 mL, 0.1 M). After 6 h, the reaction was subjected to a workup process outlined in General Procedure A and purified *via* gravity column chromatography using EtOAc/hexane = 15:85 (v/v) as the eluent to afford the title product as a white solid (0.077 g, 83%). Characterisation data were consistent with the literature values.[5]

^{1}H NMR (500 MHz, DMSO-d$_6$) δ 9.91 (s, 1H), 8.08 (d, *J* = 7.5 Hz, 1H), 7.96 – 7.89 (m, 1H), 7.75 (d, *J* = 8.1 Hz, 1H), 7.70 (d, *J* = 7.2 Hz, 1H), 7.57 – 7.51 (m, 2H), 7.48 (t, *J* = 7.8 Hz, 1H), 2.19 (s, 3H).

^{13}C{^{1}H} NMR (126 MHz, DMSO-d$_6$) δ 168.8, 133.6, 128.0, 127.6, 125.8, 125.6, 125.4, 124.9, 122.6, 121.4, 23.4.

IR (film): ν_{max} 3264, 3048, 1268, 2029, 1960, 1651, 1535, 1504, 1342, 1273, 1165, 1018, 957 cm^{-1}.

Melting point: 160 – 162 °C.

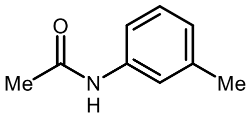

***N*-(*m*-tolyl)acetamide (3j)**: According to General Procedure A, *m*-methylaniline (0.08 g, 0.5 mmol, 1 equiv.), catalyst B (0.036 g, 20 mol%, 0.2 equiv.), thioacetic acid (0.07 mL, 1 mmol, 2 equiv.), and MeCN (5 mL, 0.1 M). After 6 h, the reaction was subjected to a workup process outlined in General Procedure A and purified *via* gravity column chromatography using EtOAc/hexane = 15:85 (v/v) as the eluent to afford the title product as a white solid (0.058 g, 78%). Characterisation data were consistent with the literature values.[5]

^{1}H NMR (500 MHz, $CDCl_3$) δ 7.61 (d, J = 7.7 Hz, 1H), 7.40 (s, 1H), 7.15 (d, J = 4.4 Hz, 2H), 7.06 (t, J = 7.0 Hz, 1H), 2.20 (s, 3H), 2.13 (s, 3H).

^{13}C{^{1}H} NMR (126 MHz, $CDCl_3$) δ 168.7, 135.7, 130.5, 130.2, 126.6, 125.5, 124.0, 24.0, 17.8.

IR (film): ν_{max} 3287, 1643, 1589, 1528, 1458, 1366, 1265, 1119, 1042, 856 cm^{-1}.

Melting point: 110 – 112 °C.

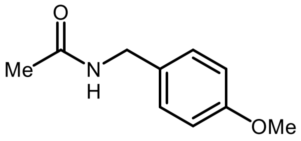

***N*-(*m*-tolyl)acetamide (3o)**: According to General Procedure A, *p*-methoxybenzylamine (0.065 mL, 0.5 mmol, 1 equiv.), catalyst B (0.036 g, 20 mol%, 0.2 equiv.), thioacetic acid (0.07 mL, 1 mmol, 2 equiv.), and MeCN (5 mL, 0.1 M). After 6 h, the reaction was subjected to a workup process outlined in General Procedure A and purified *via* gravity column chromatography using EtOAc/hexane = 15:85 (v/v) as the eluent to afford the title product as a white solid (0.061 g, 69%). Characterisation data were consistent with the literature values.[10]

^{1}H NMR (500 MHz, $CDCl_3$) δ 7.18 (d, J = 8.3 Hz, 2H), 6.84 (d, J = 8.3 Hz, 2H), 6.13 (s, 1H), 4.31 (d, J = 5.5 Hz, 2H), 3.77 (s, 3H), 1.97 (s, 3H).

^{13}C{^{1}H} NMR(126 MHz, $CDCl_3$) δ 170.1, 159.0, 130.4, 129.2, 114.1, 55.3, 43.2, 23.2.

IR (film): ν_{max} 3279, 2832, 1767, 1628, 1551, 1504, 1435, 1366, 1288, 1242, 1173, 1103, 1026, 810, 748 cm^{-1}.

Melting point: 88 – 90 °C.

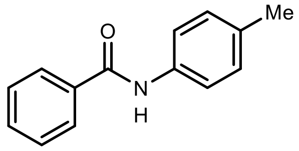

***N*-(*p*-tolyl)benzamide (3p)**: According to General Procedure A, 4-methylaniline (0.054 g, 0.5 mmol, 1 equiv.), catalyst B (0.036 g, 20 mol%, 0.2 equiv.), benzothioic *S*-acid (0.14 g, 1 mmol, 2 equiv.), and MeCN (5 mL, 0.1 M). After 6 h, the reaction was subjected to a workup process outlined in General Procedure A and purified *via* gravity column chromatography using EtOAc/hexane = 15:85 (v/v) as the eluent to afford the title product as a white solid (0.086 g, 81%). Characterisation data were consistent with the literature values.[5]

^{1}H NMR (500 MHz, $CDCl_3$) δ 8.04 (s, 1H), 7.84 (d, *J* = 7.4 Hz, 2H), 7.51 (t, *J* = 9.1 Hz, 3H), 7.43 (t, *J* = 7.5 Hz, 2H), 7.14 (d, *J* = 7.9 Hz, 2H), 2.33 (s, 3H).

^{13}C{^{1}H} NMR (126 MHz, $CDCl_3$) δ 165.9, 135.5, 135.1, 134.2, 131.7, 129.6, 128.7, 127.1, 120.5, 21.0.

IR (film): ν_{max} 3310, 1643, 1597, 1512, 1404, 1319, 1265, 1026, 903, 810 cm^{-1}.

Melting point: 156 – 157 °C.

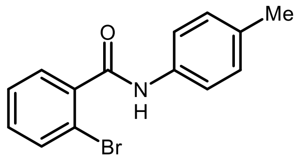

2-bromo-*N*-(*p*-tolyl)benzamide (3q): According to General Procedure A, 4-methylaniline (0.054 g, 0.5 mmol, 1 equiv.), catalyst B (0.036 g, 20 mol%, 0.2 equiv.), 2-bromobenzothioic *S*-acid (0.22 g, 1 mmol, 2 equiv.), and MeCN (5 mL, 0.1 M). After 6 h, the reaction was subjected to a workup process outlined in General Procedure A and purified *via* gravity column chromatography using EtOAc/hexane = 15:85 (v/v) as the eluent to afford the title product as a white solid (0.113 g, 78%). Characterisation data were consistent with the literature values.[11]

^{1}H NMR (500 MHz, $CDCl_3$) δ 7.94 (s, 1H), 7.59 – 7.46 (m, 4H), 7.33 (t, *J* = 7.4 Hz, 1H), 7.26 (dd, *J* = 10.9, 4.4 Hz, 1H), 7.14 (d, *J* = 8.1 Hz, 2H), 2.34 (s, 3H).

^{13}C{^{1}H} NMR (126 MHz, $CDCl_3$) δ 165.6, 137.9, 135.1, 134.5, 133.4, 131.5, 129.7, 129.6, 127.6, 120.3, 119.4, 21.0.

IR (film): ν_{max} 3233, 1651, 1597, 1396, 1327, 1258, 1111, 1026, 949, 895, 810 cm^{-1}.

Melting point: 140 – 142 °C.

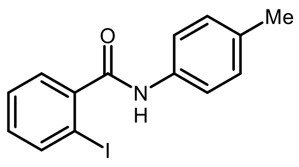

2-iodo-*N*-(*p*-tolyl)benzamide (3r): According to General Procedure A, 4-methylaniline (0.054 g, 0.5 mmol, 1 equiv.), catalyst B (0.036 g, 20 mol%, 0.2 equiv.), 2-iodobenzothioic *S*-acid (0.26 g, 1 mmol, 2 equiv.), and MeCN (5 mL, 0.1 M). After 6 h, the reaction was subjected to a workup process outlined in General Procedure A and purified *via* gravity column chromatography using EtOAc/hexane = 15:85 (v/v) as the eluent to afford the title product as a white solid (0.118 g, 70%). Characterisation data were consistent with the literature values.[12]

^{1}H NMR (500 MHz, DMSO-d_6) δ 10.20 (s, 1H), 8.30 (d, *J* = 7.9 Hz, 1H), 8.04 (d, *J* = 8.2 Hz, 2H), 7.93 – 7.75 (m, 2H), 7.55 (d, *J* = 8.0 Hz, 3H), 2.75 (s, 3H).

^{13}C{^{1}H} NMR (126 MHz, DMSO-d_6) δ 167.1, 142.7, 138.9, 135.7, 133.0, 130.3, 128.7, 127.7, 127.5, 119.7, 92.5, 20.3.

IR (film): ν_{max} 3233, 3109, 1736, 1651, 1597, 1535, 1450, 1396, 1327, 1258, 1227, 1111, 1011, 895, 810 cm^{-1}.

Melting point: 179 – 180 °C.

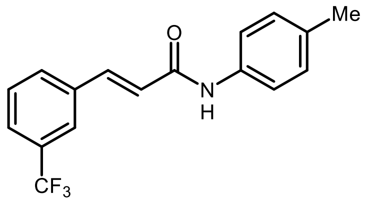

***N*-(*p*-tolyl)-3-(3-(trifluoromethyl)phenyl)acrylamide (3s)**: According to General Procedure A, 4-methylaniline (0.054 g, 0.5 mmol, 1 equiv.), catalyst B (0.036 g, 20 mol%, 0.2 equiv.), 3-(3-(trifluoromethyl)phenyl)prop-2-enethioic *S*-acid (0.23 g, 1 mmol, 2 equiv.), and MeCN (5 mL, 0.1 M). After 6 h, the reaction was subjected to a workup process outlined in General Procedure A and purified *via* gravity column chromatography using EtOAc/hexane = 15:85 (v/v) as the eluent to afford the title product as a white solid (0.128 g, 84%).

^{1}H NMR (500 MHz, DMSO-d_6) δ 10.03 (s, 1H), 8.17 – 7.75 (m, 7H), 7.46 (d, *J* = 7.5 Hz, 2H), 7.26 (d, *J* = 15.6 Hz, 1H), 2.66 (s, 3H).

^{13}C{^{1}H} NMR (126 MHz, DMSO) δ 163.0, 138.0, 135.8, 135.5, 132.7, 130.7, 128.9, 128.7, 125.1, 124.61 – 122.21 (q, *J* = 360.3 Hz, 1C), 20.2.

IR (film): ν_{max} 3194, 3132, 2978, 1659, 1605, 1512, 1450, 1404, 1373, 1304, 1250, 1196, 1072, 1018, 926, 810 cm^{-1}.

LC–MS: *m/z* calculated for $C_{17}H_{14}F_3NO$ $[M–H]^-$ 304.0954, found 304.0952.

Melting point: 187 – 189 °C.

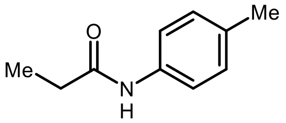

***N*-(*p*-tolyl)propionamide (3t)**: According to General Procedure A, 4-methylaniline (0.054 g, 0.5 mmol, 1 equiv.), catalyst B (0.036 g, 20 mol%, 0.2 equiv.), propanethioic *S*-acid (0.09 g, 1 mmol, 2 equiv.), and MeCN (5 mL, 0.1 M). After 6 h, the reaction was subjected to a workup process outlined in General Procedure A and purified *via* gravity column chromatography using EtOAc/hexane = 15:85 (v/v) as the eluent to afford the title product as a white solid (0.128 g, 77%). Characterisation data were consistent with the literature values.[13]

^{1}H NMR (500 MHz, $CDCl_3$) δ 8.02 (s, 1H), 7.41 (d, *J* = 7.5 Hz, 2H), 7.07 (d, *J* = 7.4 Hz, 2H), 2.35 (dd, *J* = 14.7, 7.3 Hz, 2H), 2.29 (s, 3H), 1.20 (t, *J* = 7.4 Hz, 3H).

^{13}C{^{1}H} NMR (126 MHz, $CDCl_3$) δ 172.6, 135.7, 133.6, 129.3, 120.3, 30.5, 20.8, 9.8.

IR (film): ν_{max} 3194, 3132, 2978, 1659, 1605, 1512, 1450, 1404, 1373, 1304, 1250, 1196, 1072, 1018, 926, 810 cm^{-1}.

Melting point: 130 – 132 °C.

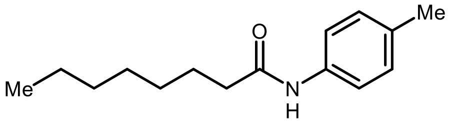

***N*-(*p*-tolyl)octanamide (3u)**: According to General Procedure A, 4-methylaniline (0.054 g, 0.5 mmol, 1 equiv.), catalyst B (0.036 g, 20 mol%, 0.2 equiv.), octanoic *S*-acid (0.16 g, 1 mmol, 2 equiv.), and MeCN (5 mL, 0.1 M). After 6 h, the reaction was subjected to a workup process outlined in General Procedure A and purified *via* gravity column chromatography using EtOAc/hexane = 15:85 (v/v) as the eluent to afford the title product as a white solid (0.092 g, 79%). Characterisation data were consistent with the literature values.[14]

^{1}H NMR (500 MHz, $CDCl_3$) δ 7.59 (s, 1H), 7.40 (d, *J* = 8.2 Hz, 2H), 7.09 (d, *J* = 8.1 Hz, 2H), 2.32 (s, 1H), 2.30 (s, 3H), 1.75 – 1.62 (m, 2H), 1.38 – 1.16 (m, 9H), 0.88 (t, *J* = 6.8 Hz, 3H).

^{13}C{^{1}H} NMR (126 MHz, $CDCl_3$) δ 171.7, 135.6, 133.7, 129.4, 120.1, 37.8, 31.8, 29.3, 29.1, 25.8, 22.7, 20.9, 14.1.

IR (film): ν_{max} 3302, 2916, 2855, 1659, 1597, 1528, 1466, 1404, 1296, 1250, 1180, 1111, 964, 818 cm^{-1}.

Melting point: 80 – 82 °C.

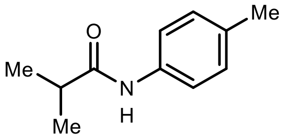

***N*-(*p*-tolyl)octanamide (3v)**: According to General Procedure A, 4-methylaniline (0.054 g, 0.5 mmol, 1 equiv.), catalyst B (0.036 g, 20 mol%, 0.2 equiv.), 2-methylpropanethioic *S*-acid (0.10 g, 1 mmol, 2 equiv.), and MeCN (5 mL, 0.1 M). After 6 h, the reaction was subjected to a workup process outlined in General Procedure A and purified *via* gravity column chromatography using EtOAc/hexane = 15:85 (v/v) as the eluent to afford the title product as a white solid (0.062 g, 70%). Characterisation data were consistent with the literature values.[15]

^{1}H NMR (500 MHz, $CDCl_3$) δ 7.64 (d, J = 8.1 Hz, 2H), 7.17 (d, J = 7.6 Hz, 2H), 6.14 (s, 1H), 4.29 – 4.20 (m, 1H), 2.35 (s, 3H), 1.22 (d, J = 6.6 Hz, 6H).

^{13}C{^{1}H} NMR (126 MHz, $CDCl_3$) δ 166.7, 141.5, 132.2, 129.1, 126.9, 41.8, 22.8, 21.4.

IR (film): ν_{max} 3302, 2970, 1628, 1535, 1458, 1342, 1288, 1165, 1134, 880, 826 cm^{-1}.

Melting point: 108 – 110 °C

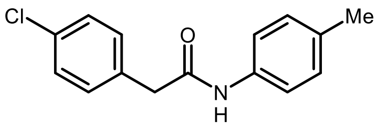

2-(4-chlorophenyl)-*N*-(*p*-tolyl)acetamide (3w): According to General Procedure A, 4-methylaniline (0.054 g, 0.5 mmol, 1 equiv.), catalyst B (0.036 g, 20 mol%, 0.2 equiv.), 2-(4-chlorophenyl)ethanethioic *S*-acid (0.19 g, 1 mmol, 2 equiv.), and MeCN (5 mL, 0.1 M). After 6 h, the reaction was subjected to a workup process outlined in General Procedure A and purified *via* gravity column chromatography using EtOAc/hexane = 15:85 (v/v) as the eluent to afford the title product as a white solid (0.101 g, 78%). Characterisation data were consistent with the literature values.[16]

^{1}H NMR (500 MHz, DMSO) δ 9.89 (s, 1H), 7.49 (d, J = 7.6 Hz, 2H), 7.32 (dd, J = 28.6, 7.5 Hz, 4H), 7.07 (d, J = 7.5 Hz, 2H), 3.64 (s, 2H), 2.28 (s, 3H).

^{13}C{^{1}H} NMR (126 MHz, DMSO) δ 168.2, 136.0, 134.3, 132.1, 131.5, 130.3, 128.5, 127.8, 119.2, 42.4, 20.2.

IR (film): ν_{max} 3302, 2916, 1651, 1597, 1527, 1404, 810 cm^{-1}.

Melting point: 98 – 100 °C.

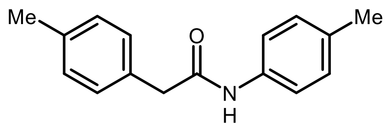

2-(4-chlorophenyl)-*N*-(*p*-tolyl)acetamide (3x): According to General Procedure A, 4-methylaniline (0.054 g, 0.5 mmol, 1 equiv.), catalyst B (0.036 g, 20 mol%, 0.2 equiv.), 2-(4-methylphenyl)ethanethioic *S*-acid (0.17 g, 1 mmol, 2 equiv.), and MeCN (5 mL, 0.1 M). After 6 h, the reaction was subjected to a workup process outlined in General Procedure A and purified *via* gravity column chromatography using EtOAc/hexane = 15:85 (v/v) as the eluent to afford the title product as a white solid (0.098 g, 82%). Characterisation data were consistent with the literature values.[10]

^{1}H NMR (500 MHz, $CDCl_3$) δ 7.28 (t, *J* = 7.8 Hz, 2H), 7.21 (s, 4H), 7.07 (d, *J* = 7.7 Hz, 2H), 3.68 (s, 2H), 2.37 (s, 3H), 2.28 (s, 3H).

^{13}C{^{1}H} NMR (126 MHz, $CDCl_3$) δ 169.3, 137.5, 135.2, 134.1, 131.5, 130.0, 129.5, 129.5, 120.0, 44.5, 21.2, 20.9.

IR (film): ν_{max} 3342, 2916, 1651, 1605, 1543, 1512, 1404, 1358, 1258, 1165, 1119, 1018, 810, 756 cm^{-1}.

Melting point: 162 – 164 °C.

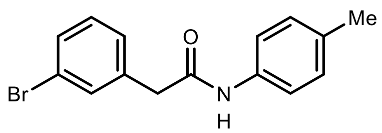

2-(3-bromophenyl)-*N*-(*p*-tolyl)acetamide (3y): According to General Procedure A, 4-methylaniline (0.054 g, 0.5 mmol, 1 equiv.), catalyst B (0.036 g, 20 mol%, 0.2 equiv.), 2-(3-bromophenyl)ethanethioic *S*-acid (0.23 g, 1 mmol, 2 equiv.), and MeCN (5 mL, 0.1 M). After 6 h, the reaction was subjected to a workup process outlined in General Procedure A and purified *via* gravity column chromatography using EtOAc/hexane = 15:85 (v/v) as the eluent to afford the title product as a white solid (0.122 g, 80%).

^{1}H NMR (500 MHz, $CDCl_3$) δ 7.65 (s, 1H), 7.42 – 7.33 (m, 2H), 7.25 (d, *J* = 7.8 Hz, 2H), 7.21 – 7.09 (m, 2H), 7.01 (d, *J* = 7.9 Hz, 2H), 3.55 (s, 2H), 2.23 (s, 3H).

^{13}C{^{1}H} NMR (126 MHz, $CDCl_3$) δ 168.7, 136.9, 135.0, 134.4, 132.5, 130.6, 130.5, 129.5, 128.1, 123.0, 120.4, 43.9, 20.9.

IR (film): ν_{max} 3302, 2916, 1651, 1528, 818 cm^{-1}.

LC–MS: *m/z* calculated for $C_{15}H_{14}{}^{81}BrNO$ $[M–H]^-$ 306.0311, found 306.0311.

Melting point: 198 – 198 °C.

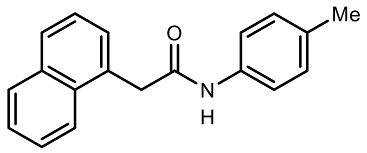

2-(naphthalen-1-yl)-*N*-(*p*-tolyl)acetamide (3z): According to General Procedure A, 4-methylaniline (0.054 g, 0.5 mmol, 1 equiv.), catalyst B (0.036 g, 20 mol%, 0.2 equiv.), 2-(naphthalen-1-yl)ethanethioic *S*-acid (0.20 g, 1 mmol, 2 equiv.), and MeCN (5 mL, 0.1 M). After 6 h, the reaction was subjected to a workup process outlined in General Procedure A and purified *via* gravity column chromatography using EtOAc/hexane = 15:85 (v/v) as the eluent to afford the title product as a white solid (0.114 g, 83%). Characterisation data were consistent with the literature values.[16]

^{1}H NMR (500 MHz, $CDCl_3$) δ 8.03 (d, *J* = 7.9 Hz, 1H), 7.88 (ddd, *J* = 9.0, 7.7, 4.6 Hz, 2H), 7.57 – 7.45 (m, 4H), 7.18 (d, *J* = 8.3 Hz, 2H), 7.03 (t, *J* = 11.0 Hz, 3H), 4.16 (s, 2H), 2.25 (s, 3H).

^{13}C{^{1}H} NMR (126 MHz, $CDCl_3$) δ 169.0, 135.0, 134.2, 134.2, 132.2, 130.9, 129.4, 129.0, 128.9, 128.5, 127.1, 126.4, 125.8, 123.8, 120.2, 42.9, 20.9.

IR (film): ν_{max} 3287, 1659, 1597, 1512, 1404, 1342, 1296, 1250, 957, 810 cm^{-1}.

Melting point: 190 – 193 °C.

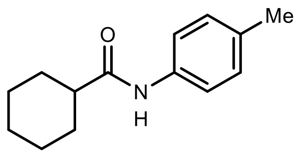

N-(*p*-tolyl)cyclohexanecarboxamide **(3aa)**: According to General Procedure A, 4-methylaniline (0.054 g, 0.5 mmol, 1 equiv.), catalyst B (0.036 g, 20 mol%, 0.2 equiv.), cyclohexanecarbothioic *S*-acid (0.14 g, 1 mmol, 2 equiv.), and MeCN (5 mL, 0.1 M). After 6 h, the reaction was subjected to a workup process outlined in General Procedure A and purified *via* gravity column chromatography using EtOAc/hexane = 15:85 (v/v) as the eluent to afford the title product as a white solid (0.114 g, 71%). Characterisation data were consistent with the literature values.[17]

^{1}H NMR (500 MHz, $CDCl_3$) δ 7.72 (s, 1H), 7.42 (d, *J* = 7.7 Hz, 2H), 7.07 (d, *J* = 7.7 Hz, 2H), 2.29 (s, 3H), 2.22 (d, *J* = 11.4 Hz, 1H), 1.91 (d, *J* = 12.3 Hz, 2H), 1.79 (d, *J* = 9.5 Hz, 2H), 1.68 (s, 1H), 1.53 (dd, *J* = 23.0, 11.3 Hz, 2H), 1.24 (dd, *J* = 22.2, 13.2 Hz, 3H).

^{13}C{^{1}H} NMR (126 MHz, $CDCl_3$) δ 174.7, 135.8, 133.5, 129.3, 120.1, 46.3, 29.7, 25.7, 25.7, 20.8.

IR (film): ν_{max} 2924, 2855, 1651, 1597, 1512, 1443, 1404, 1288, 1250, 1196, 1034, 957, 895, 810 cm^{-1}.

Melting point: 152 – 153 °C.

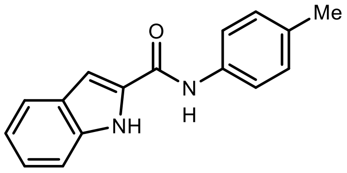

N-(*p*-tolyl)-1*H*-indole-2-carboxamide **(3ab)**: According to General Procedure A, 4-methylaniline (0.054 g, 0.5 mmol, 1 equiv.), catalyst B (0.036 g, 20 mol%, 0.2 equiv.), 1*H*-indole-2-carbothioic *S*-acid (0.18 g, 1 mmol, 2 equiv.), and MeCN (5 mL, 0.1 M). After 6 h, the reaction was subjected to a workup process outlined in General Procedure A and purified *via* gravity column chromatography using EtOAc/hexane = 15:85 (v/v) as the eluent to afford the title product as a white solid (0.108 g, 86%). Characterisation data were consistent with the literature values.[18]

^{1}H NMR (500 MHz, DMSO-d$_6$) δ 11.38 (s, 1H), 9.86 (s, 1H), 7.57 (d, *J* = 47.0 Hz, 3H), 7.35 (d, *J* = 52.6 Hz, 2H), 7.04 (t, *J* = 34.6 Hz, 4H), 2.22 (s, 3H).

^{13}C{^{1}H} NMR (126 MHz, DMSO-d$_6$) δ 159.5, 136.6, 136.1, 132.4, 131.5, 128.7, 127.0, 123.3, 121.3, 120.1, 119.6, 112.0, 103.7, 20.4.

IR (film): ν_{max} 2429, 1651, 1597, 1512, 1404, 1250, 810 cm^{-1}.

Melting point: 252 – 253 °C.

4.3 General Procedure B

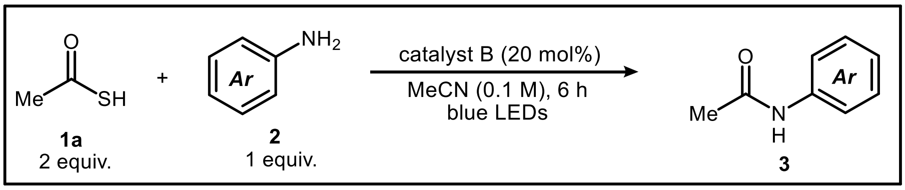

To a 100 ml round-bottom flask was added aniline **2** (0.9 mL, 0.5 mmol, 1 equiv.), catalyst B (0.72 g, 20 mol%, 0.2 equiv.), MeCN (5 mL, 0.1 M) and thioacetic acid **1a** (1.4 mL, 1 mmol, 2 equiv.). The reaction flask was capped and irradiated using setup 2. After 6 h, the reaction mixture was quenched with brine (50 mL) and extracted with EtOAc (6 × 20 mL). The combined organic layers were dried under anhydrous Na_2SO_4, concentrated under reduced pressure and purified *via* gravity column chromatography on silica gel using 15% EtOAc in *n*-hexanes to afford the desired products **3**.

Characterisation Data for Products Generated from General Procedure B

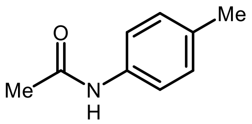

***N*-(*p*-tolyl)acetamide (3a)**: According to General Procedure A, *p*-toluidine (0.05 g, 0.5 mmol, 1 equiv.), catalyst B (0.72 g, 20 mol%, 0.2 equiv.), thioacetic

acid (0.07 mL, 1 mmol, 2 equiv.), and MeCN (5 mL, 0.1 M). After 6 h, the reaction was subjected to a workup process outlined in General Procedure A and purified *via* gravity column chromatography using EtOAc/hexane = 15:85 (v/v) as the eluent to afford the title product as a white solid (1.00 g, 67%). Characterisation data were consistent with the literature values.[3]

^{1}H NMR (500 MHz, $CDCl_3$) δ 7.72 (s, 1H), 7.37 (d, *J* = 8.0 Hz, 2H), 7.09 (d, *J* = 7.9 Hz, 2H), 2.30 (s, 3H), 2.13 (s, 3H).

^{13}C{^{1}H} NMR (126 MHz, $CDCl_3$) δ 168.7, 135.5, 133.9, 129.5, 120.3, 24.4, 20.9.

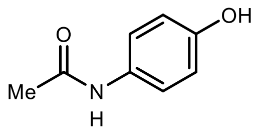

***N*-(*p*-bromophenyl)acetamide (3ab)**: According to General Procedure A, 4-hydroxyaniline (1.09 g, 0.5 mmol, 1 equiv.), catalyst B (0.72 g, 20 mol%, 0.2 equiv.), thioacetic acid (0.07 mL, 1 mmol, 2 equiv.), and MeCN (5 mL, 0.1 M). After 6 h, the reaction was subjected to a workup process outlined in General Procedure A and purified *via* gravity column chromatography using EtOAc/hexane = 15:85 (v/v) as the eluent to afford the title product as an off-white solid (1.04 g, 69%). Characterisation data were consistent with the literature values.[5]

^{1}H NMR (500 MHz, DMSO-d_6) δ 9.63 (s, 1H), 9.12 (s, 1H), 7.34 (d, *J* = 8.5 Hz, 2H), 6.68 (d, *J* = 8.5 Hz, 2H), 1.98 (s, 3H).

^{13}C{^{1}H} NMR (126 MHz, DMSO-d_6) δ 167.5, 153.1, 131.0, 120.8, 115.0, 23.7.

IR (film): ν_{max} 2429, 1754, 1651, 1605, 1512, 1250, 802 cm^{-1}.

Melting point: 170 – 172 °C.

4.4 Mechanistic Studies

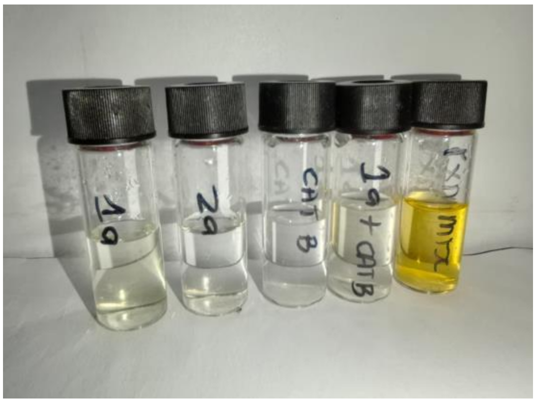

Figure S3: Separate reaction components (from left to right) **1a**, **2a** and catalyst **B**, **1a** + catalyst **B**, and appearance of the coloured EDA complex between **1a**, **2b** and catalyst **B**. [**1a**] = 0.2 M, [**2a**] = 0.1 M and [catalyst B] = 0.02 M.

UV-vis study between 1a, catalyst B, and K_2CO_3

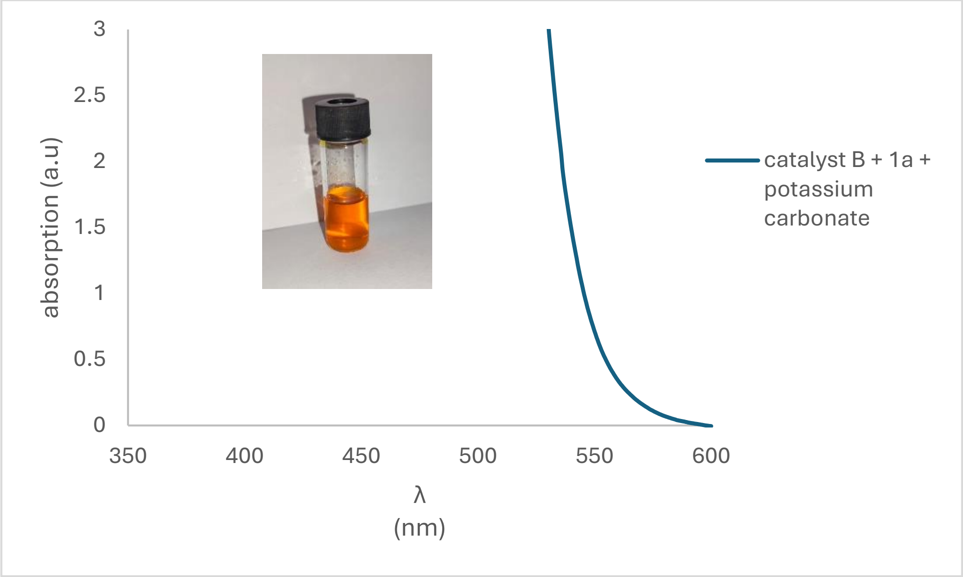

Figure S3. Optical absorption spectra, recorded in MeCN in 1 mm path quartz cuvettes of the separate reaction components thioacetic acid **1a**, catalyst **B** and K_2CO_3, and appearance of the coloured EDA complex between thioacetic acid **1a**, catalyst B, and K_2CO_3. [1a] = 0.2 M, [K_2CO_3] = 0.2 M and [catalyst **B**] = 0.02 M.

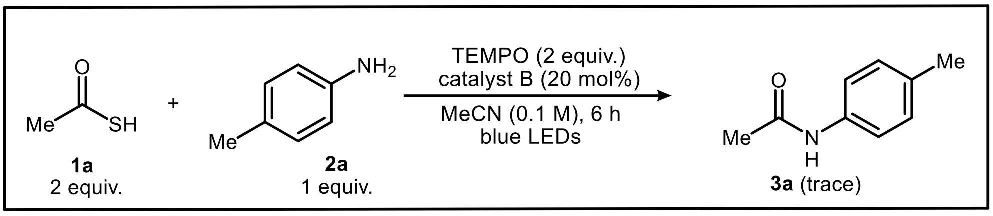

To a 20 ml reaction vial was added 4-methylaniline **2** (0.5 mmol, 1 equiv.), catalyst B (0.036 g, 20 mol%, 0.2 equiv.), TEMPO (0.156 g, 1 mmol, 2 equiv.), MeCN (5 mL, 0.1 M) and thioacetic acid **1** (1 mmol, 2 equiv.). The reaction vial was capped and irradiated using setup 1 for 6 h. The use of 1 equiv. of TEMPO afforded **3a** with 43% yield.

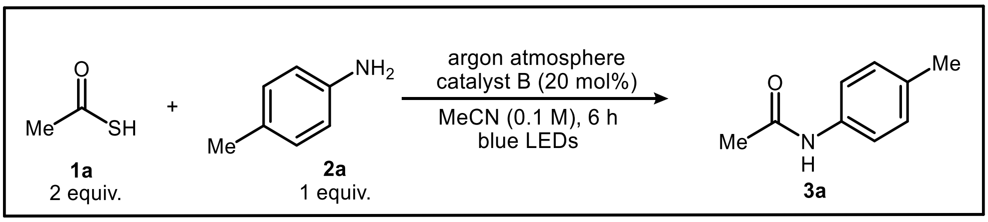

To a 20 ml reaction vial was added 4-methylaniline **2a** (0.5 mmol, 1 equiv.), catalyst B (0.036 g, 20 mol%, 0.2 equiv.), MeCN (5 mL, 0.1 M). The reaction was argon-sparged for 5 minutes, then thioacetic acid **1a** (1 mmol, 2 equiv.) was added. The reaction *via* was capped and irradiated using setup 1. After 6 h, the reaction mixture was quenched with brine (10 mL) and extracted with EtOAc (3 × 20 mL). The combined organic layers were dried under anhydrous Na_2SO_4, concentrated under reduced pressure and purified *via* gravity column chromatography on silica gel using EtOAc/hexane = 15:85 (v/v) as the eluent to afford the desired product **3a** as a white solid (0.028 g, 37%). Characterisation data were consistent with the literature values.[3]

^{1}H NMR (500 MHz, $CDCl_3$) δ 7.72 (s, 1H), 7.37 (d, *J* = 8.0 Hz, 2H), 7.09 (d, *J* = 7.9 Hz, 2H), 2.30 (s, 3H), 2.13 (s, 3H).

^{13}C{^{1}H} NMR (126 MHz, $CDCl_3$) δ 168.7, 135.5, 133.9, 129.5, 120.3, 24.4, 20.9.

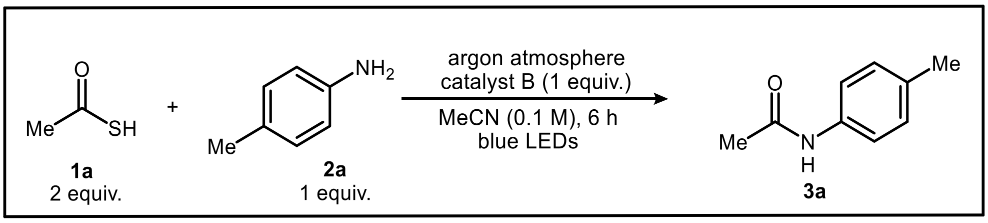

To a 20 ml reaction vial was added 4-methylaniline **2a** (0.5 mmol, 1 equiv.), catalyst B (0.036 g, 1 equiv.), MeCN (5 mL, 0.1 M). The reaction was argon-sparged for 5 minutes, then thioacetic acid **1a** (1 mmol, 2 equiv.) was added. The reaction *via* was capped and irradiated using setup 1. After 6 h, the reaction mixture was quenched with brine (10 mL) and extracted with EtOAc (3 × 20 mL). The

combined organic layers were dried under anhydrous Na_2SO_4, concentrated under reduced pressure and purified *via* gravity column chromatography using EtOAc/hexane = 15:85 (v/v) as the eluent to afford the desired product **3a** as a white solid (0.065 g, 88%). Characterisation data were consistent with the literature values.[3]

^{1}H NMR (500 MHz, $CDCl_3$) δ 7.72 (s, 1H), 7.37 (d, *J* = 8.0 Hz, 2H), 7.09 (d, *J* = 7.9 Hz, 2H), 2.30 (s, 3H), 2.13 (s, 3H).

^{13}C{^{1}H} NMR (126 MHz, $CDCl_3$) δ 168.7, 135.5, 133.9, 129.5, 120.3, 24.4, 20.9.

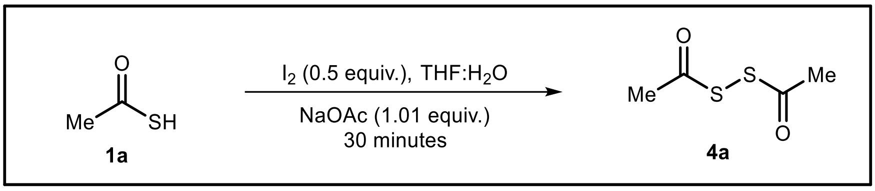

To an oven-dried 100 mL round-bottom flask containing thioacetic acid (0.35 mL, 5 mmol), THF (10 mL), NaOAc (0.42 g, 5.05 mmol) and H_2O (5 mL), was added I_2 (0.63 g, 2.5 mmol). After 30 minutes, the reaction mixture was quenched with H_2O (20 mL) and extracted with EtOAc (3 × 20 mL). The combined organic layers were washed with a saturated $Na_2S_2O_3$ solution, dried over anhydrous Na_2SO_4 and concentrated under reduced pressure. The resulting pale yellow oil was used without further purification.[19] Characterisation data were consistent with the literature values.[20]

^{1}H NMR (500 MHz, $CDCl_3$) δ 2.46 (s, 6H).

^{13}C{^{1}H} NMR (126 MHz, $CDCl_3$) δ 193.6, 30.5.

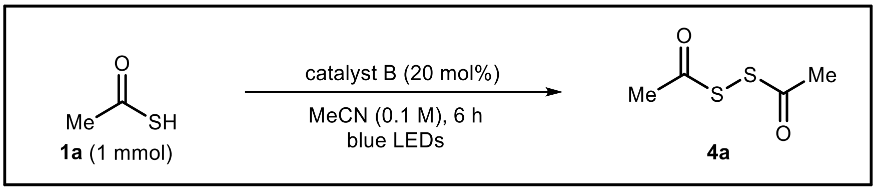

To a 20 ml reaction vial was added thioacetic acid **1a** (0.07 mL, 1 mmol, 1 equiv.), catalyst B (0.036 g, 1 equiv.), MeCN (5 mL, 0.1 M). The reaction *via* was capped and irradiated using setup 1. After 6 h, the reaction mixture was quenched with brine (10 mL) and extracted with EtOAc (3 × 20 mL). The combined organic layers were dried under anhydrous Na_2SO_4, concentrated under reduced pressure and purified *via* gravity column chromatography on silica gel using EtOAc/hexane = 10:90 (v/v) as the eluent to afford the desired product **3a** as a white solid (0.093 g, 62%). Characterisation data were consistent with the literature values.[20]

^{1}H NMR (500 MHz, $CDCl_3$) δ 2.46 (s, 6H).

^{13}C{^{1}H} NMR (126 MHz, $CDCl_3$) δ 193.6, 30.5.

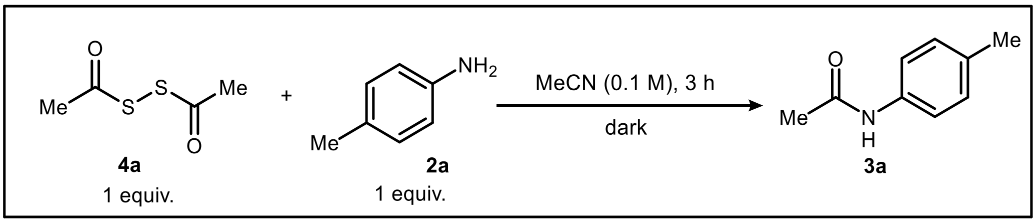

To a 20 ml reaction vial was added 4-methylaniline **2a** (0.5 mmol, 1 equiv.), acetic dithioperoxyanhydride **4a** (0.075 g, 0.5 mmol, 1 equiv.) and MeCN (5 mL, 0.1 M). The reaction *via* was capped, covered in tin foil. After 3 h, the reaction mixture was quenched with brine (10 mL) and extracted with EtOAc (3 × 20 mL). The combined organic layers were dried under anhydrous Na_2SO_4, concentrated under reduced pressure and purified *via* gravity column chromatography on silica gel using EtOAc/hexane = 15:85 (v/v) as the eluent to afford the desired product **3a** as a white solid (0.057 g, 76%). Characterisation data were consistent with the literature values.[3]

^{1}H NMR (500 MHz, $CDCl_3$) δ 7.72 (s, 1H), 7.37 (d, J = 8.0 Hz, 2H), 7.09 (d, J = 7.9 Hz, 2H), 2.30 (s, 3H), 2.13 (s, 3H).

^{13}C{^{1}H} NMR (126 MHz, $CDCl_3$) δ 168.7, 135.5, 133.9, 129.5, 120.3, 24.4, 20.9

NMR titration experiments

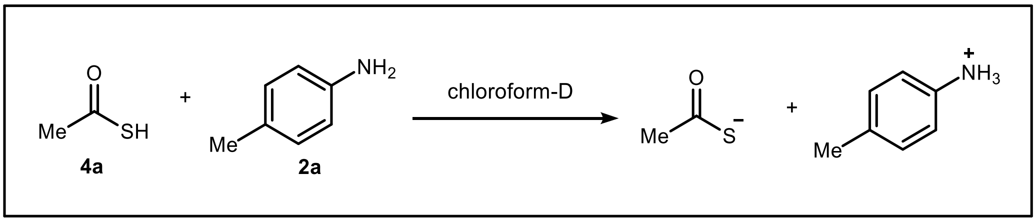

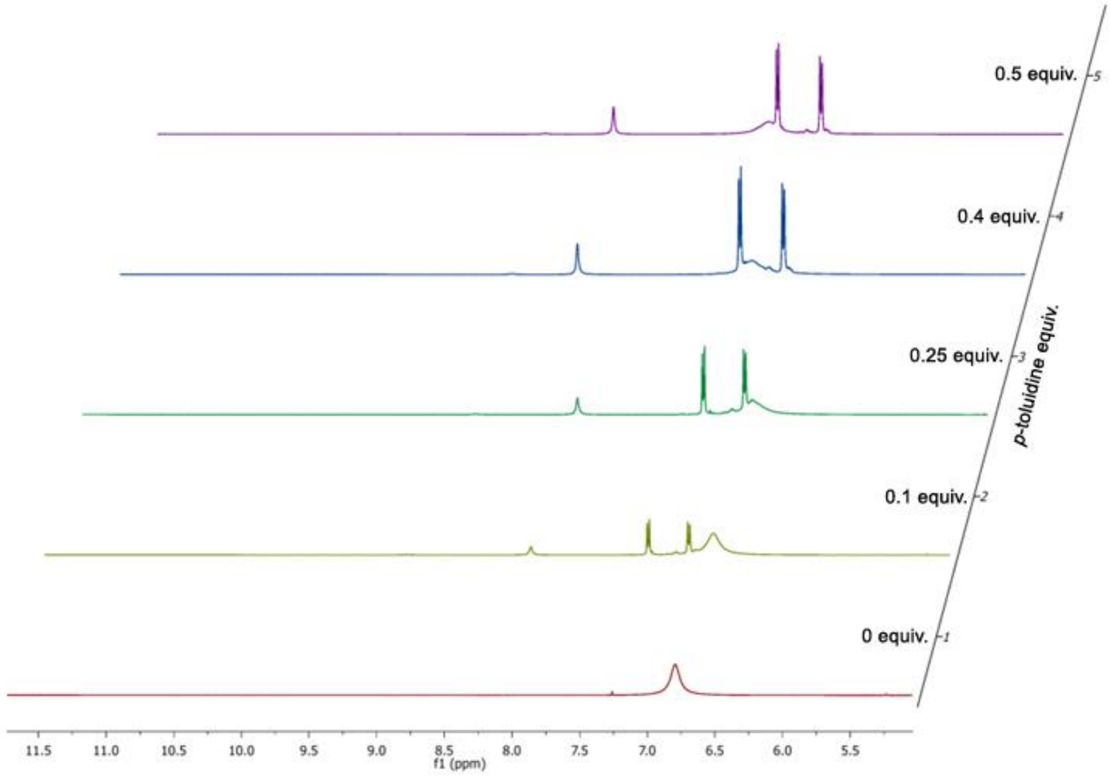

Figure S4. Overlay of ^{1}H NMR spectra acquired during the titration of thioacetic **1a** acid with *p*-toluidine **2a**. Reaction conditions: thioacetic acid **1a** (1 mmol, 0.07 mL), *p*-toluidine **2a** (0.1 equiv., 0.25 equiv., 0.4 equiv., and 0.5 equiv.).

4.4 Reference List

(1) Liu, X.; Qin, J.; Dai, W.; Zhu, Z.; Zhou, P.; Wang, Y.; Nie, J.; Yang, Y.; Zhang, Z. Metal-Free and Additive-Free Synthesis of Imides and Nitriles from Ketones via Oxidative Cleavage of C(O)–C Bonds. *ACS Catal.,* **2022**, *12*, 13300-13311.

(2) Zhou, W.; Wu, S.; Melchiorre, P. Tetrachlorophthalimides as Organocatalytic Acceptors for Electron Donor-Acceptor Complex Photoactivation. *J. Am. Chem. Soc.,* **2022**, *144*, 8914-8919.

(3) Song, W.; Dong, K.; Li, M. Visible Light-Induced Amide Bond Formation. *Org. Lett.,* **2020**, *22*, 371-375.

(4) Xiong, W.; Shi, Q.; Liu, W. H. Simple and Practical Conversion of Benzoic Acids to Phenols at Room Temperature. *J. Am. Chem. Soc.,* **2022**, *144*, 15894-15902.

(5) Procopio, D.; Marset, X.; Guillena, G.; Di Gioia, M. L.; Ramón, D. J. Visible-Light-Mediated Amide Synthesis in Deep Eutectic Solvents. *Adv. Synth. Catal.,* **2023**, *366*, 870-876.

(6) Wang, W.; Liu, J.; Yang, L.; Song, S.; Jiao, N. A Catalytic Method to Activate Nitromethane by the Cooperation of Homo- and Heterogeneous Catalysis. *Angew. Chem. Int. Ed. Engl.,* **2024**, *63*, e202312354.

(7) Straathof, N. J. W.; Tegelbeckers, B. J. P.; Hessel, V.; Wang, X.; Noël, T. A Mild and Fast Photocatalytic Trifluoromethylation of Thiols in Batch and Continuous-Flow. *Chem. Sci.,* **2014**, *5*, 4768-4773.

(8) Nabi, M.; Sharma, K.; Wandre, R. S.; Gade, A. B. One-Pot Oximation-Beckmann Rearrangement under Mild, Aqueous Micellar Conditions. *Green Chem.,* **2025**, *27*, 5332-5339.

(9) Pfeifer, V.; Certiat, M.; Bouzouita, D.; Palazzolo, A.; Garcia-Argote, S.; Marcon, E.; Buisson, D. A.; Lesot, P.; Maron, L.; Chaudret, B.; Tricard, S.; Rosal, I.; Poteau, R.; Feuillastre, S.; Pieters, G. Hydrogen Isotope Exchange Catalyzed by Ru Nanocatalysts: Labelling of Complex Molecules Containing *N*-Heterocycles and Reaction Mechanism Insights. *Chemistry* **2020**, *26*, 4988-4996.

(10) Kolesnikov, P. N.; Usanov, D. L.; Muratov, K. M.; Chusov, D. Dichotomy of Atom-Economical Hydrogen-Free Reductive Amidation *vs* Exhaustive Reductive Amination. *Org. Lett.,* **2017**, *19*, 5657-5660.

(11) Wu, Q. X.; Shu, T.; Fang, W. Y.; Qin, H. L. Discovery of KOH/$BrCH_2SO_2F$ as Water-Removable System for Clean, Mild and Robust Synthesis of Amides and Peptides. *Eur. J. Org. Chem.,* **2022**, *2022*, e202200719.

(12) Sharma, N.; Sekar, G. Stable and Reusable Binaphthyl-Supported Palladium Catalyst for Aminocarbonylation of Aryl Iodides. *Adv. Synth. Catal.* **2016**, *358*, 314-320.

(13) Li, N.; Wang, L.; Zhang, L.; Zhao, W.; Qiao, J.; Xu, X.; Liang, Z. Air-stable Bis(pentamethylcyclopentadienyl) Zirconium Perfluorooctanesulfonate as an Efficient and Recyclable Catalyst for the Synthesis of *N*-Substituted Amides. *ChemCatChem,* **2018**, *10*, 3532-3538.

(14) Li, H. C.; Li, G. N.; Sun, K.; Chen, X. L.; Jiang, M. X.; Qu, L. B.; Yu, B. Ce(III)/Photoassisted Synthesis of Amides from Carboxylic Acids and Isocyanates. *Org. Lett.,* **2022**, *24*, 2431-2435.

(15) Chirila, P. G.; Skibinski, L.; Miller, K.; Hamilton, A.; Whiteoak, C. J. Towards a Sequential One-Pot Preparation of 1,2,3-Benzotriazin-4(3H)-ones Employing a Key Cp*Co(III)-catalyzed C−H Amidation Step. *Adv. Synth. Catal.,* **2018**, *360*, 2324-2332.

(16) Yang, L. M.; Li, S. S.; Zhang, Y. Y.; Lu, J. L.; Deng, J. T.; Ma, A. J.; Zhang, X. Z.; Zhang, S. Y.; Peng, J. B. Palladium Catalyzed Aminocarbonylation of Benzylic Ammonium Triflates with Nitroarenes: Synthesis of Phenylacetamides. *Adv. Synth. Catal.,* **2021**, *363*, 2061-2065.

(17) Forni, J. A.; Micic, N.; Connell, T. U.; Weragoda, G.; Polyzos, A. Tandem Photoredox Catalysis: Enabling Carbonylative Amidation of Aryl and Alkylhalides. *Angew. Chem. Int. Ed. Engl.,* **2020**, *59*, 18646-18654.

(18) Szabo, T.; Papp, M.; Nemeth, D. R.; Dancso, A.; Volk, B.; Milen, M. Synthesis of Indolo[2,3-c]quinolin-6(7H)-ones and Antimalarial Isoneocryptolepine. Computational Study on the Pd-Catalyzed Intramolecular C-H Arylation. *J. Org. Chem.,* **2021**, *86*, 128-145.

(19) Yu, B.; Zheng, Y.; Yuan, Z.; Li, S.; Zhu, H.; De La Cruz, L. K.; Zhang, J.; Ji, K.; Wang, S.; Wang, B. Toward Direct Protein S-Persulfidation: A Prodrug Approach That Directly Delivers Hydrogen Persulfide. *J. Am. Chem. Soc.,* **2018**, *140*, 30-33.

(20) Khairullin, R. A.; Gazizov, M. B.; Kirillina, Y. S.; Gazizova, K. S.; Khayarov, K. R. Reaction of *N*-tert-butyl-2-haloaldimines with Thiolcarboxylic Acids. *Russian J. Gen. Chem.,* **2017**, *87*, 2736-2738.

4.5 Spectral Data

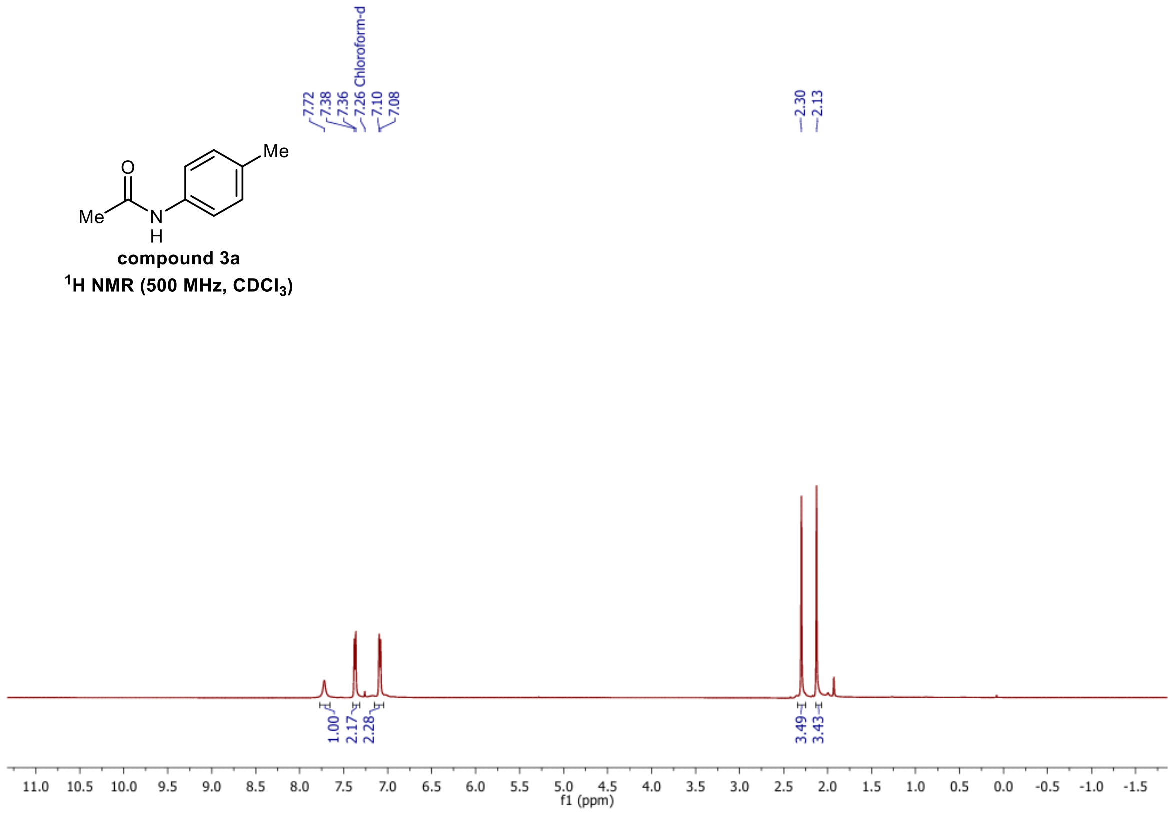

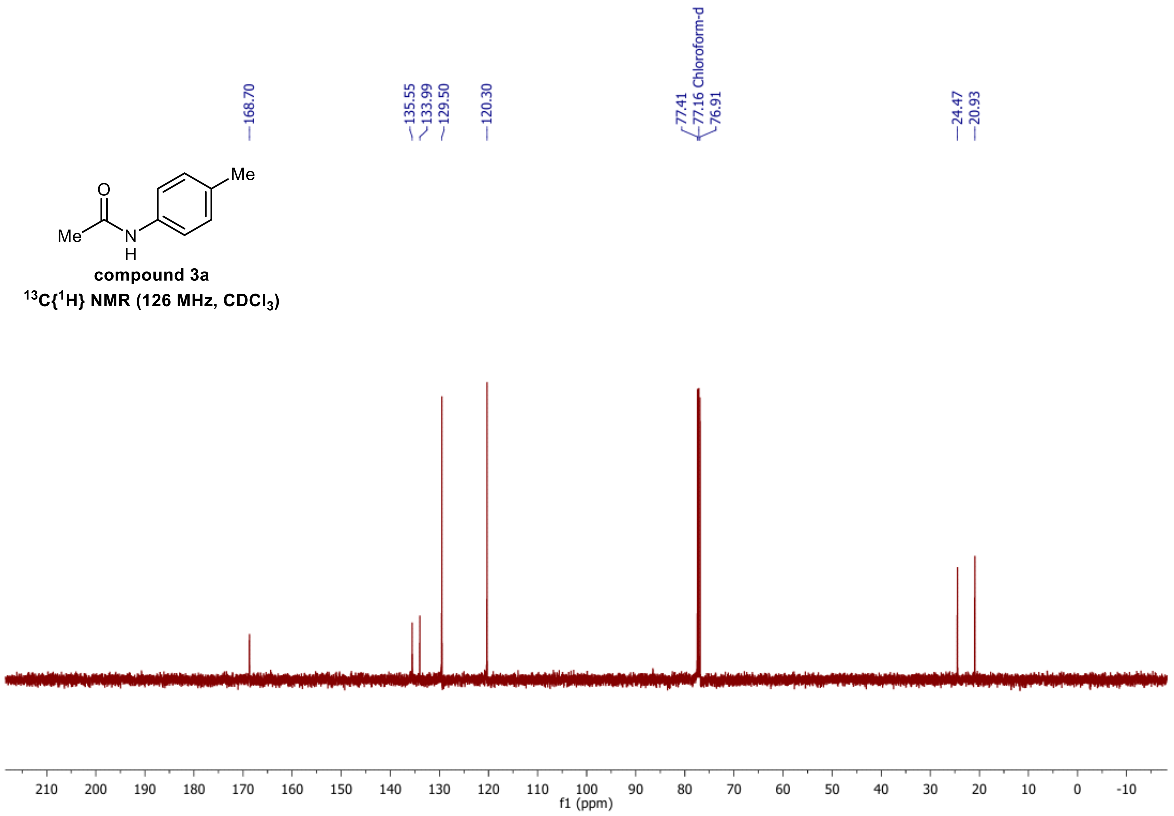
168.70
135.55
133.99
129.50
120.30
77.41
77.16 Chloroform-d
76.91
24.47
20.93
Me
O
Me
N
H
compound 3a
13C{1H} NMR (126 MHz, CDCl3)
210 200 190 180 170 160 150 140 130 120 110 100 90 80 70 60 50 40 30 20 10 0 -10
f1 (ppm)

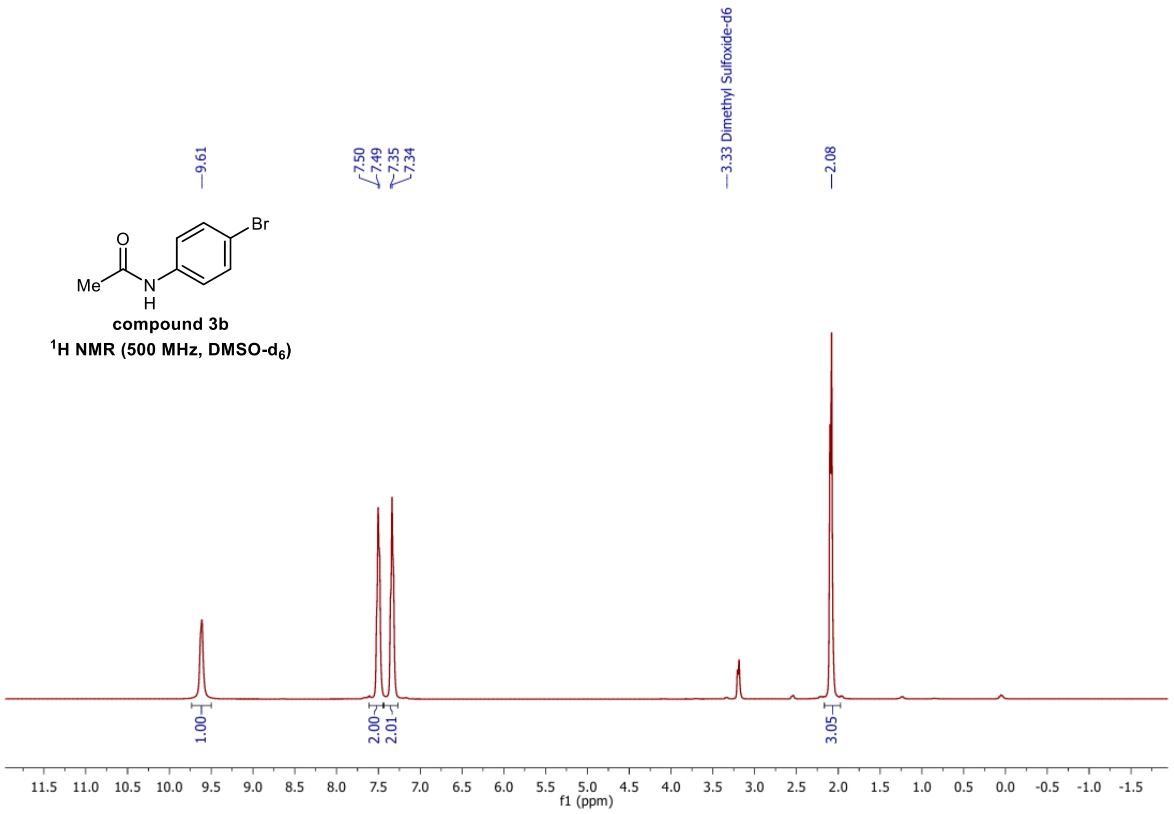
9.61
7.50
7.49
7.35
7.34
3.33 Dimethyl Sulfoxide-d6
2.08
Br
O
Me
N
H
compound 3b
1H NMR (500 MHz, DMSO-d6)
1.00
2.00
2.01
3.05
11.5 11.0 10.5 10.0 9.5 9.0 8.5 8.0 7.5 7.0 6.5 6.0 5.5 5.0 4.5 4.0 3.5 3.0 2.5 2.0 1.5 1.0 0.5 0.0 -0.5 -1.0 -1.5
f1 (ppm)

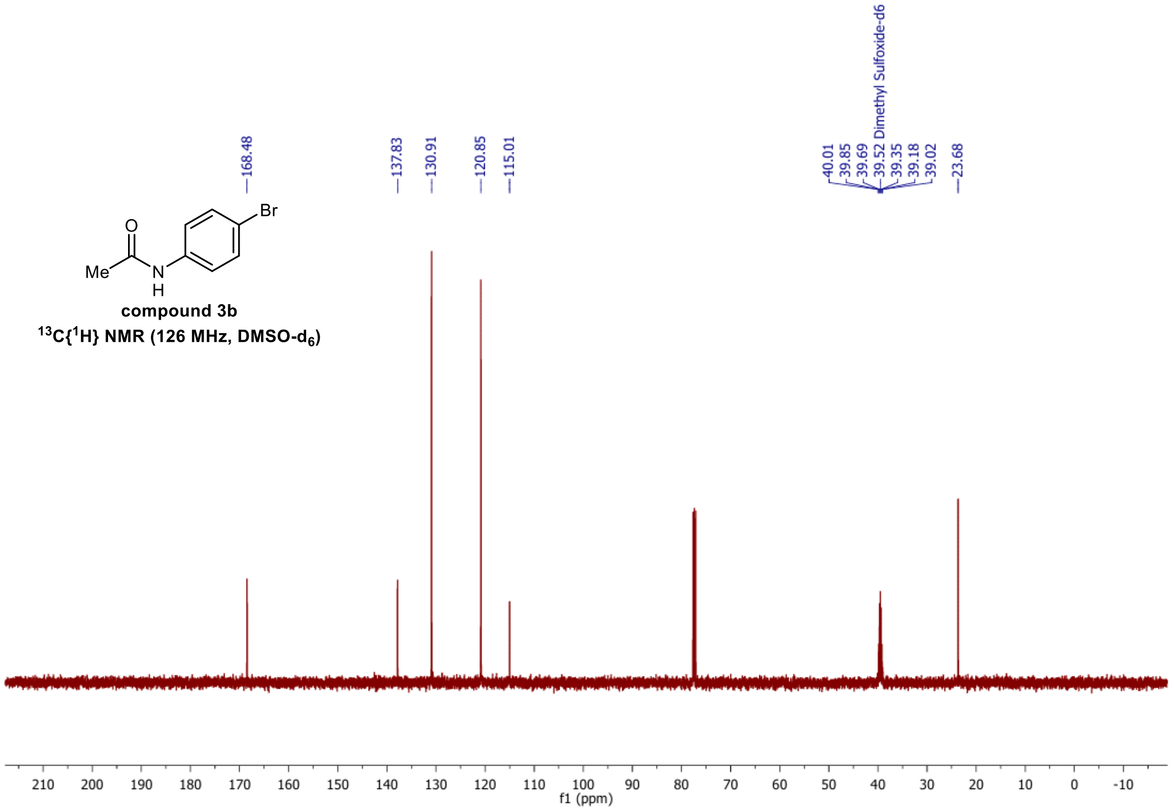

compound 3b

$^{13}C\{^{1}H\}$ NMR (126 MHz, DMSO-d_6)

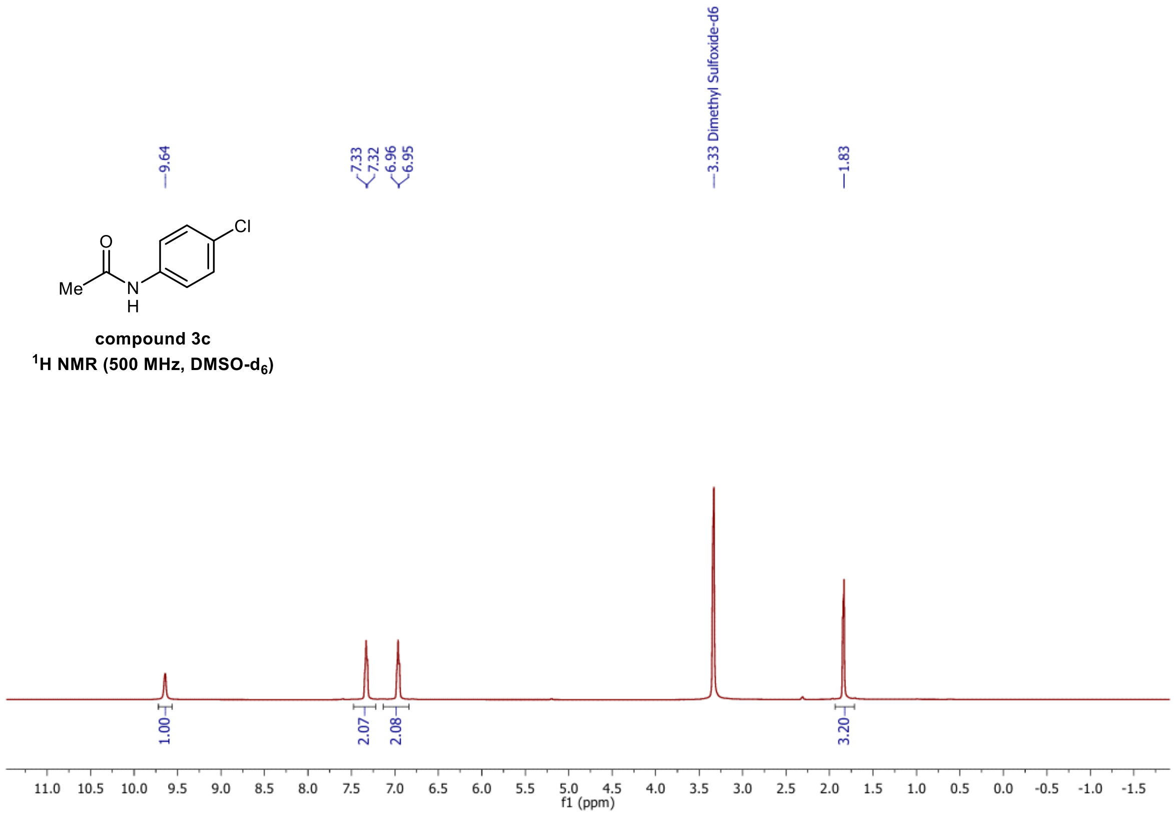

compound 3c

^{1}H NMR (500 MHz, DMSO-d_6)

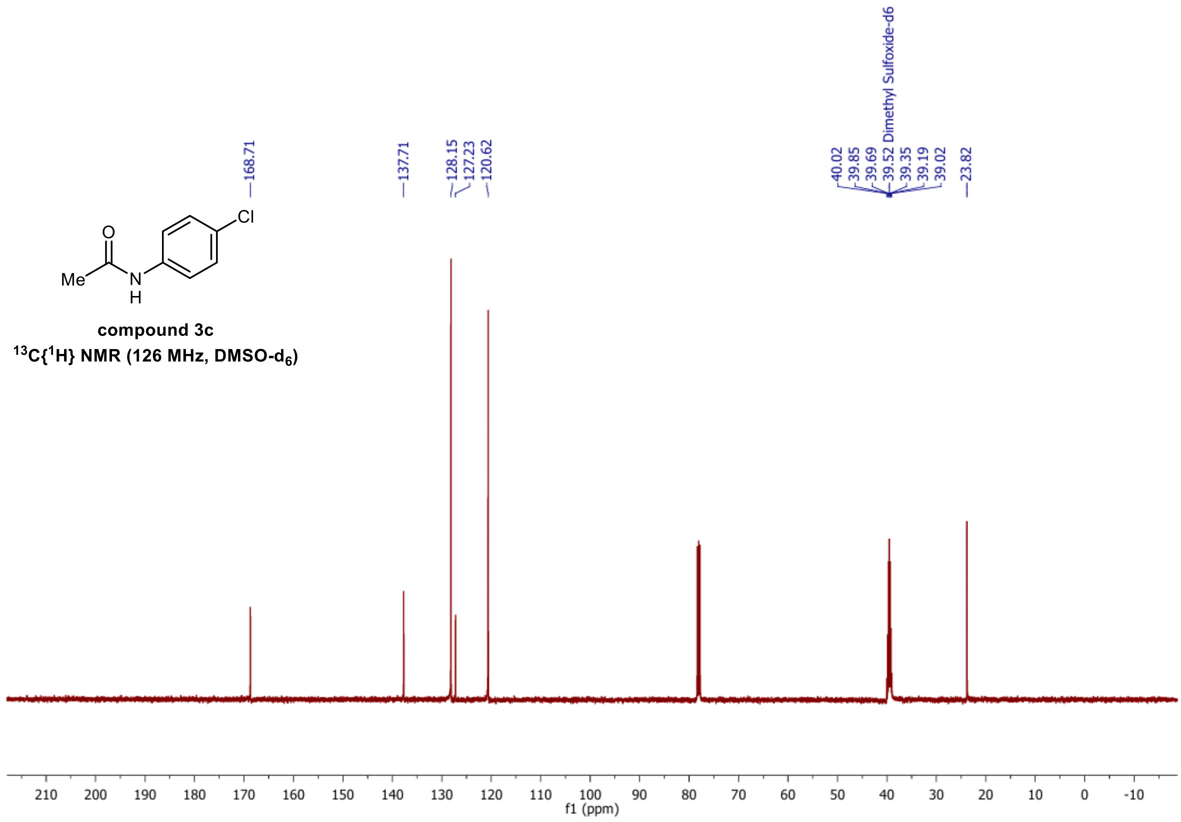

Cl
O
Me
N
H
compound 3c
13C{1H} NMR (126 MHz, DMSO-d6)
168.71
137.71
128.15
127.23
120.62
40.02
39.85
39.69
39.52 Dimethyl Sulfoxide-d6
39.35
39.19
39.02
23.82
f1 (ppm)

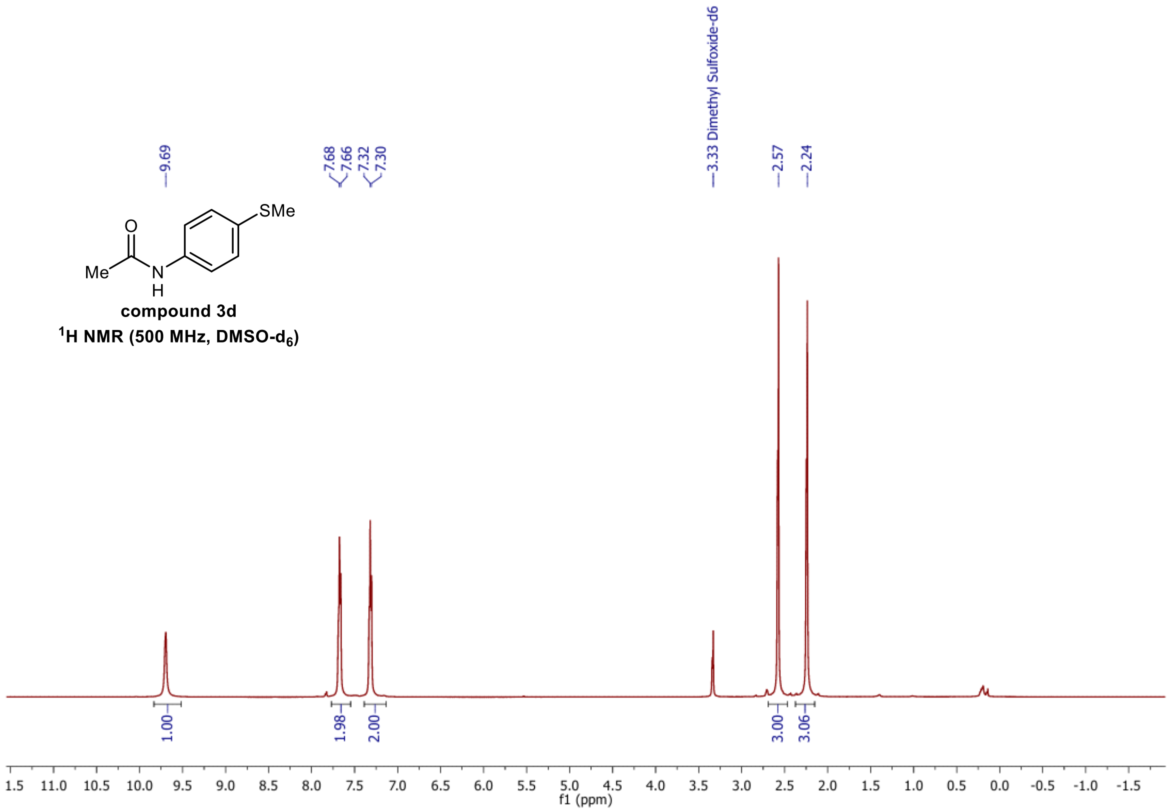

SMe
O
Me
N
H
compound 3d
1H NMR (500 MHz, DMSO-d6)
9.69
7.68
7.66
7.32
7.30
3.33 Dimethyl Sulfoxide-d6
2.57
2.24
1.00
1.98
2.00
3.00
3.06
f1 (ppm)

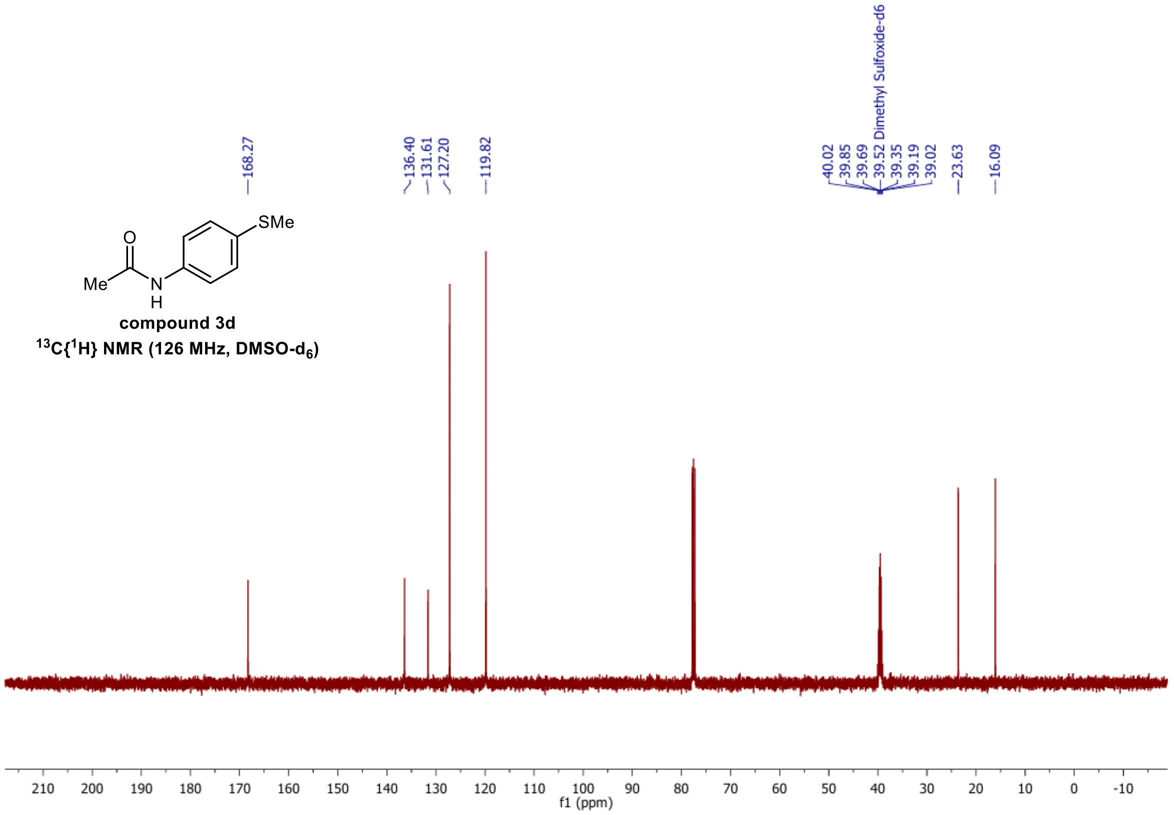

compound 3d
$^{13}C\{^{1}H\}$ NMR (126 MHz, DMSO-d_6)

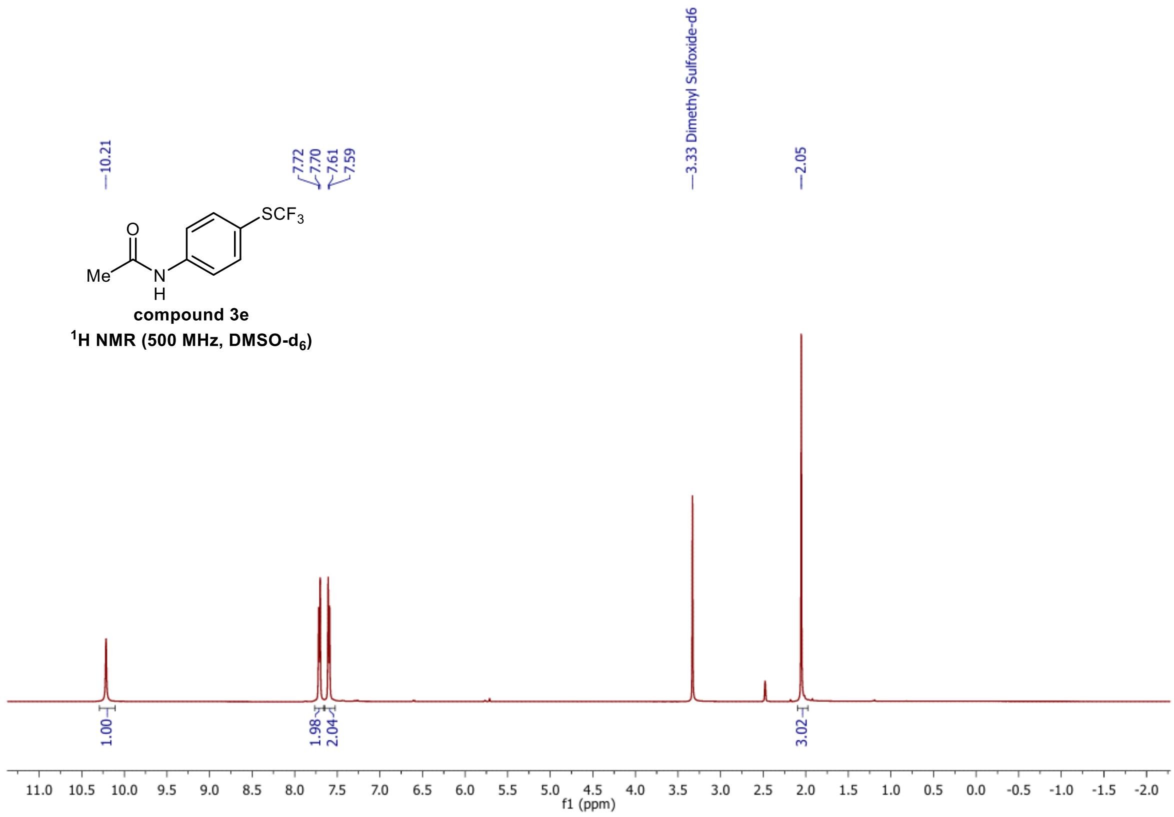

compound 3e
^{1}H NMR (500 MHz, DMSO-d_6)

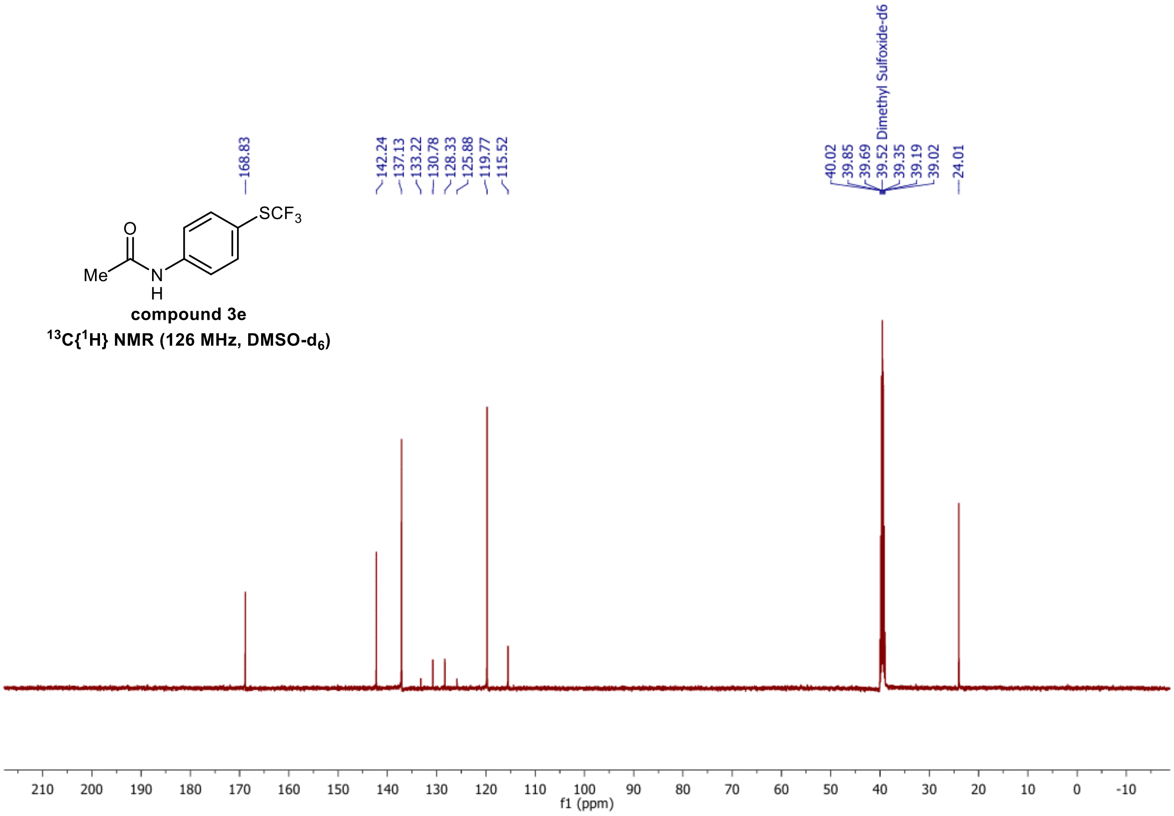

compound 3e

$^{13}C\{^1H\}$ NMR (126 MHz, DMSO-d_6)

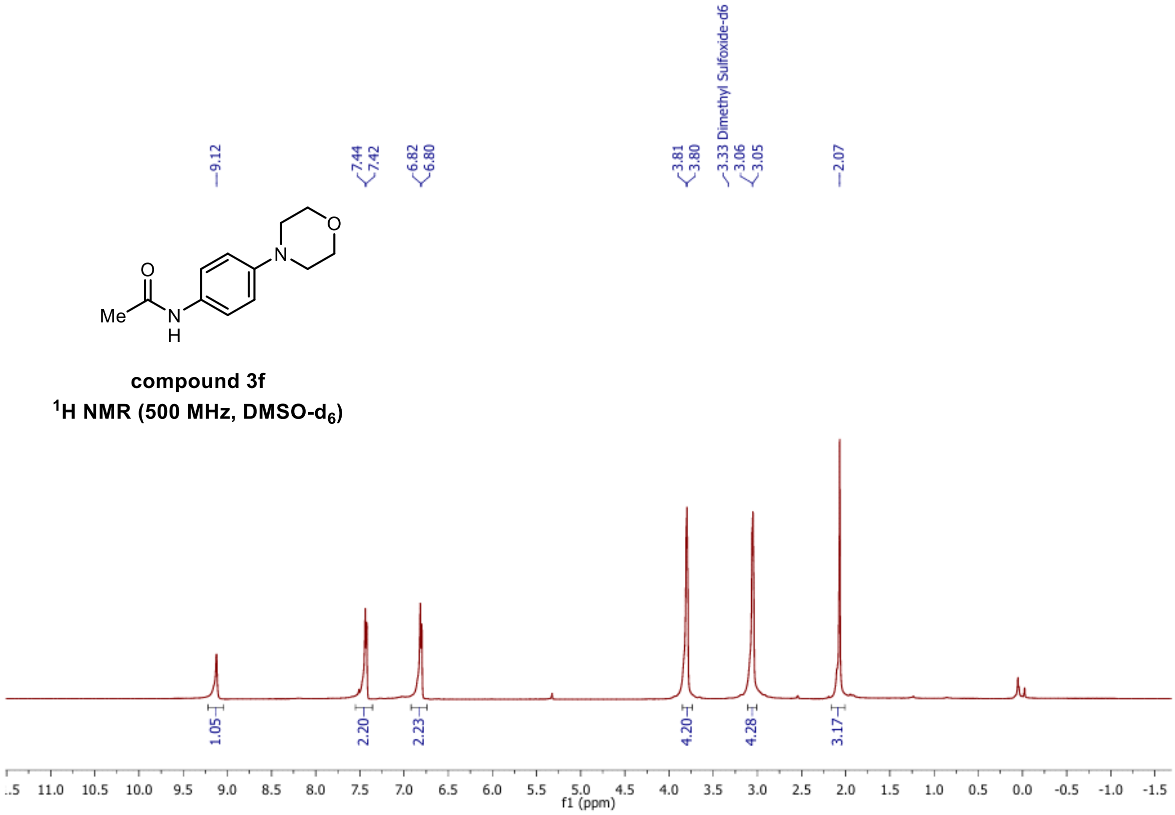

compound 3f

^1H NMR (500 MHz, DMSO-d_6)

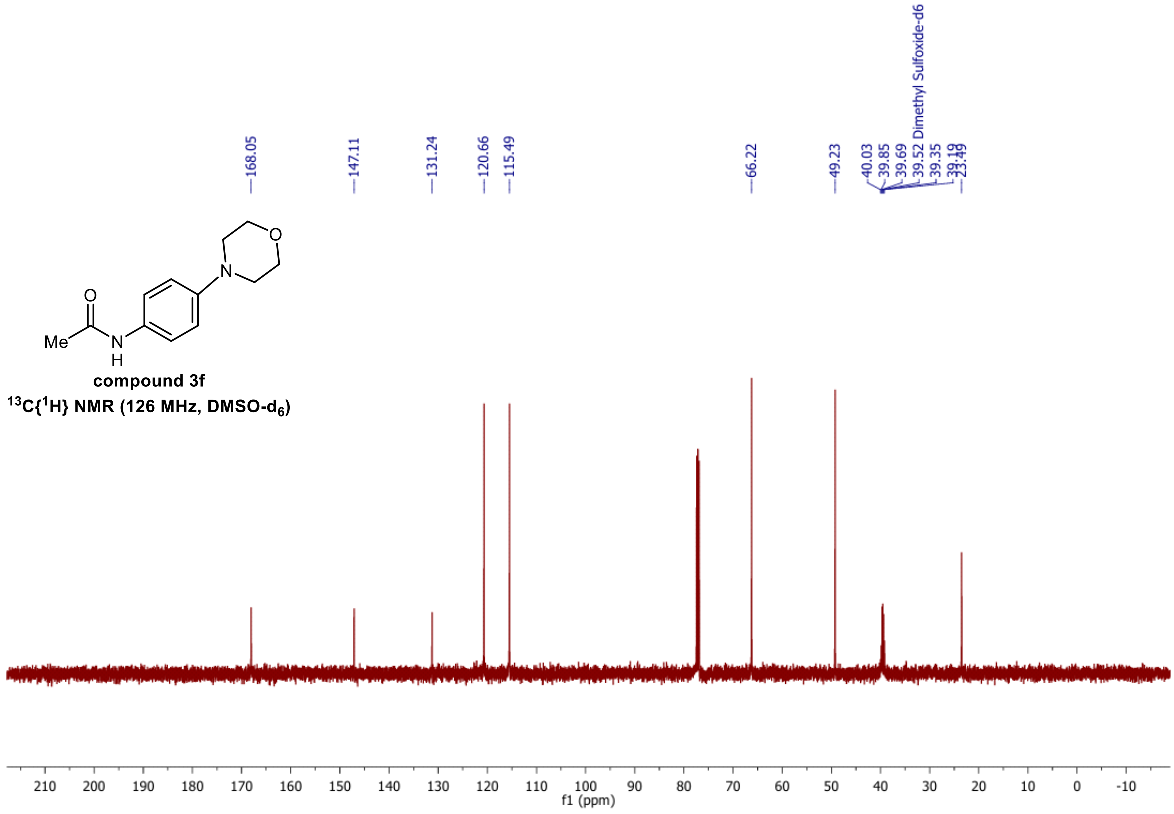
168.05
147.11
131.24
120.66
115.49
66.22
49.23
40.03
39.85
39.69
39.52 Dimethyl Sulfoxide-d6
39.35
39.19
23.49
O
N
Me
N
H
compound 3f
$^{13}C\{^1H\}$ NMR (126 MHz, DMSO-d_6)
210 200 190 180 170 160 150 140 130 120 110 100 90 80 70 60 50 40 30 20 10 0 -10
f1 (ppm)

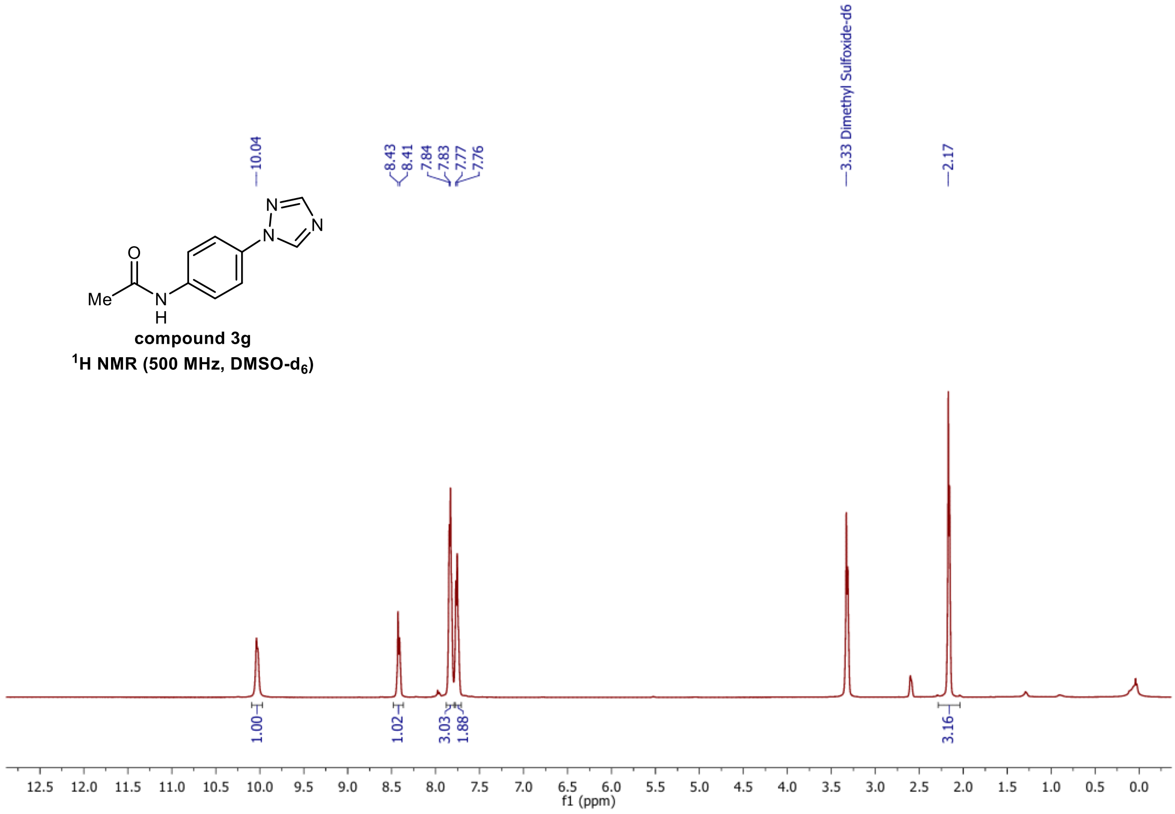
10.04
8.43
8.41
7.84
7.83
7.77
7.76
3.33 Dimethyl Sulfoxide-d6
2.17
N
N
N
O
Me
N
H
compound 3g
^1H NMR (500 MHz, DMSO-d_6)
1.00
1.02
3.03
1.88
3.16
12.5 12.0 11.5 11.0 10.5 10.0 9.5 9.0 8.5 8.0 7.5 7.0 6.5 6.0 5.5 5.0 4.5 4.0 3.5 3.0 2.5 2.0 1.5 1.0 0.5 0.0
f1 (ppm)

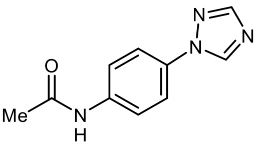

compound 3g

$^{13}C\{^{1}H\}$ NMR (126 MHz, DMSO-d_6)

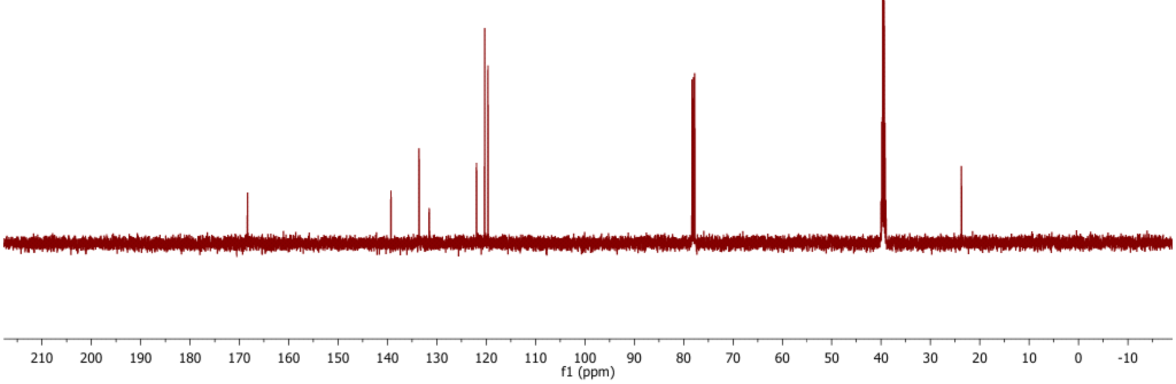

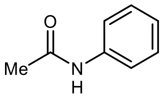

compound 3h

^{1}H NMR (500 MHz, $CDCl_3$)

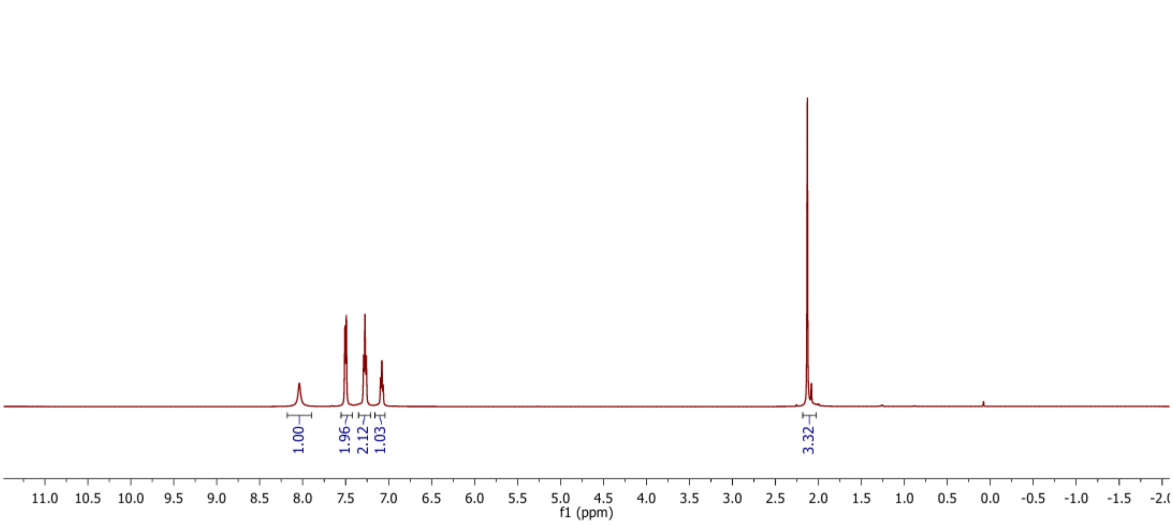

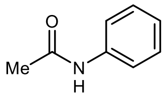

compound 3h

$^{13}C\{^1H\}$ NMR (126 MHz, $CDCl_3$)

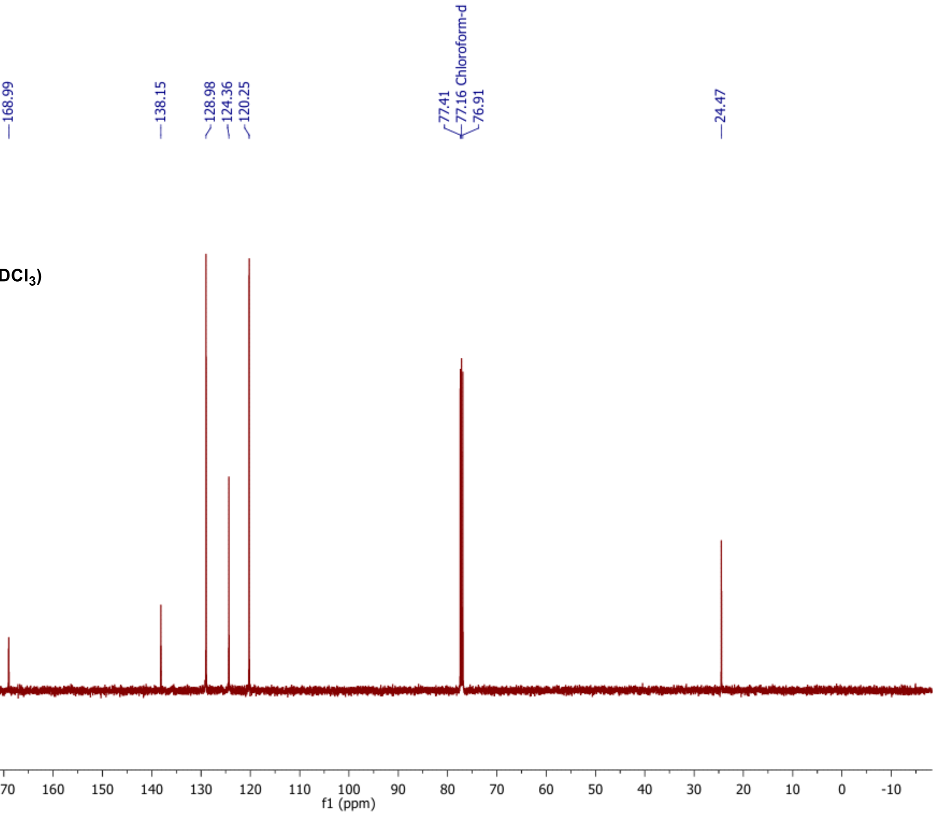

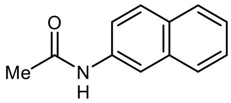

compound 3i

^1H NMR (500 MHz, DMSO-d_6)

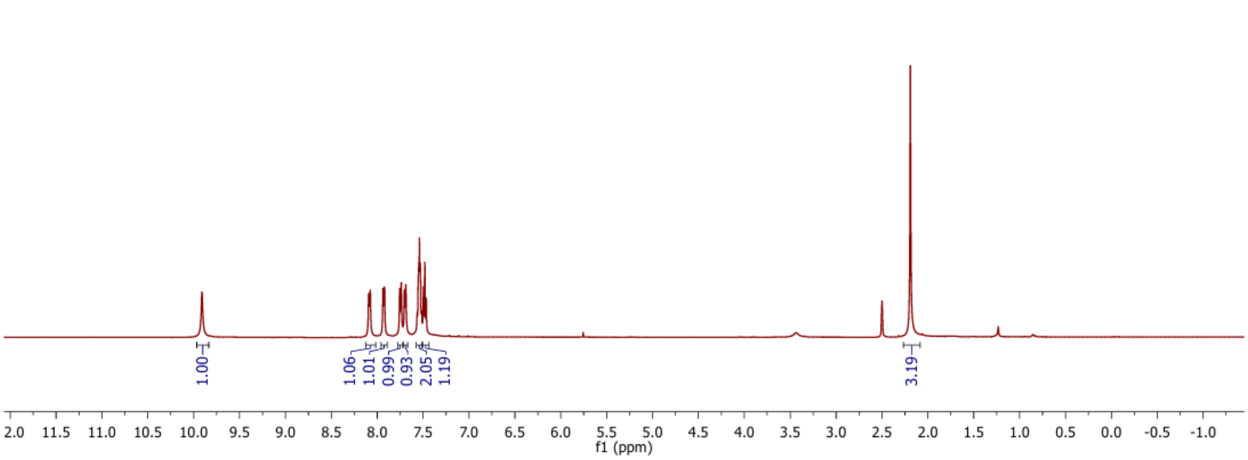

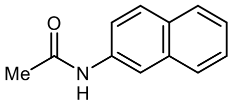

compound 3i

$^{13}C\{^{1}H\}$ NMR (126 MHz, DMSO-d_6)

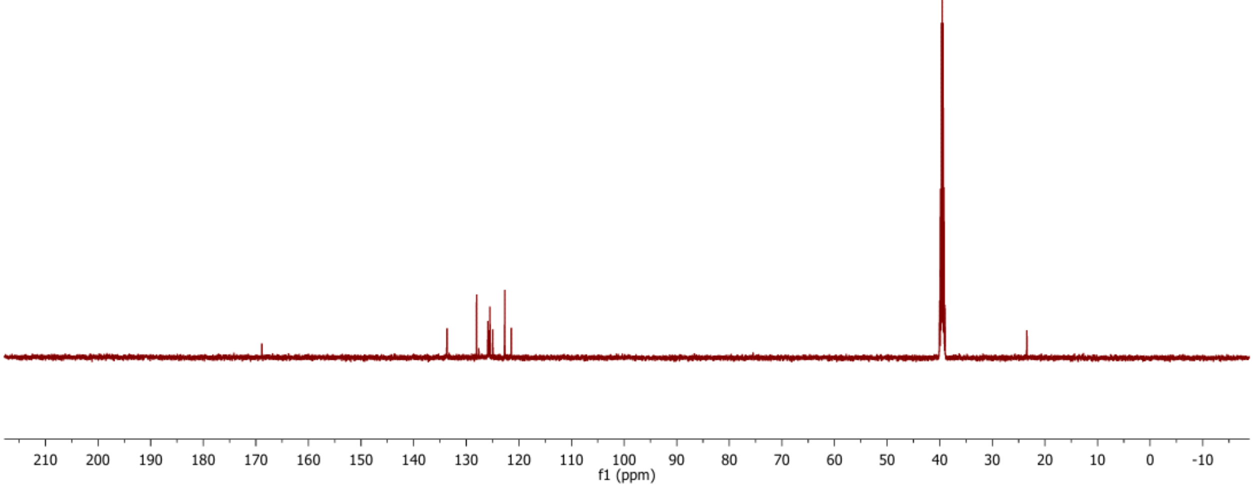

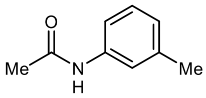

compound 3j

^{1}H NMR (500 MHz, $CDCl_3$)

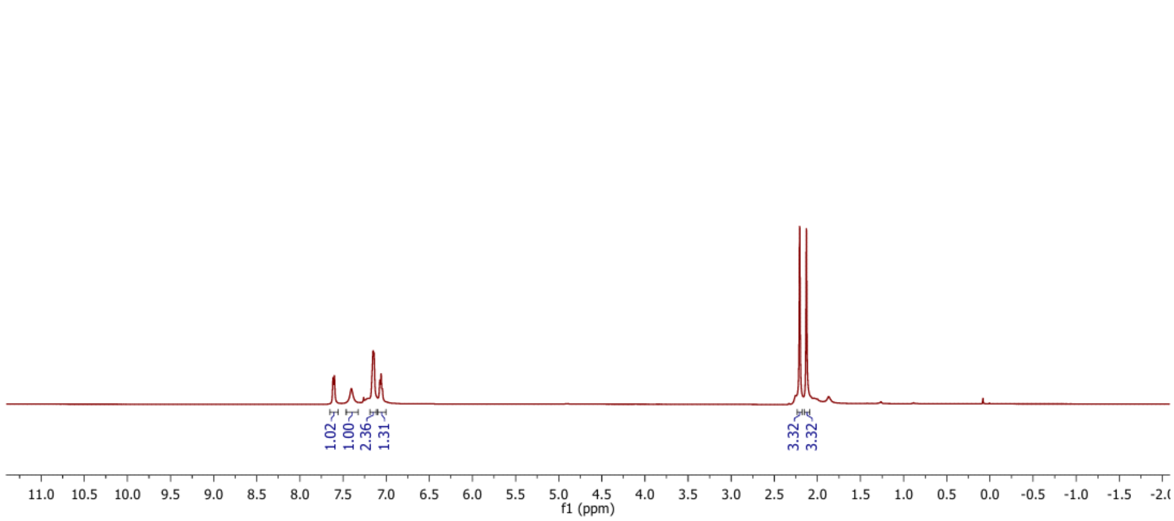

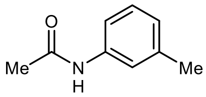

compound 3j

$^{13}C\{^{1}H\}$ NMR (126 MHz, $CDCl_3$)

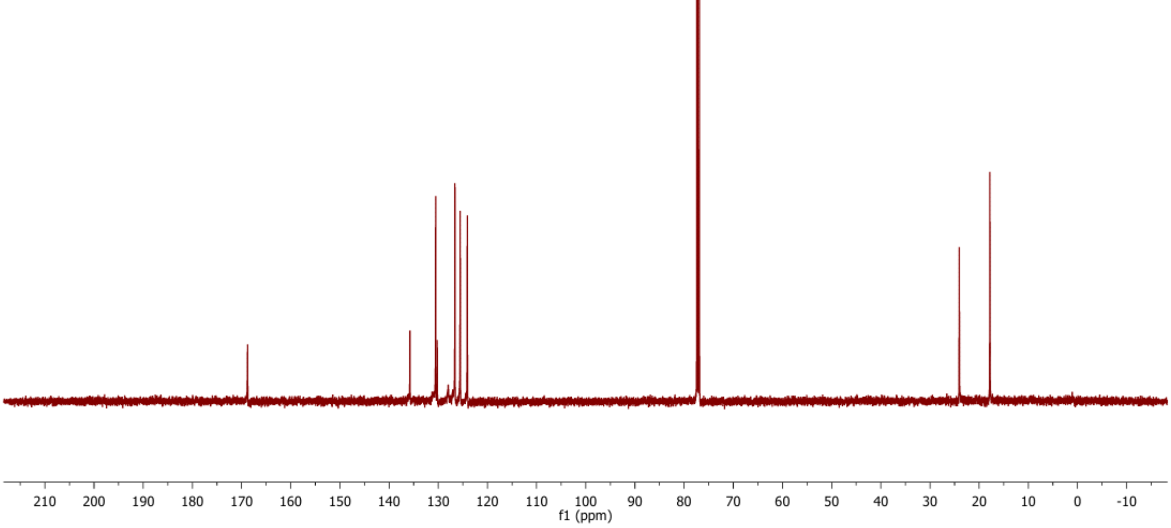

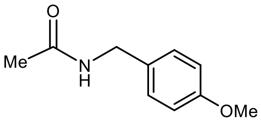

compound 3o

^{1}H NMR (500 MHz, $CDCl_3$)

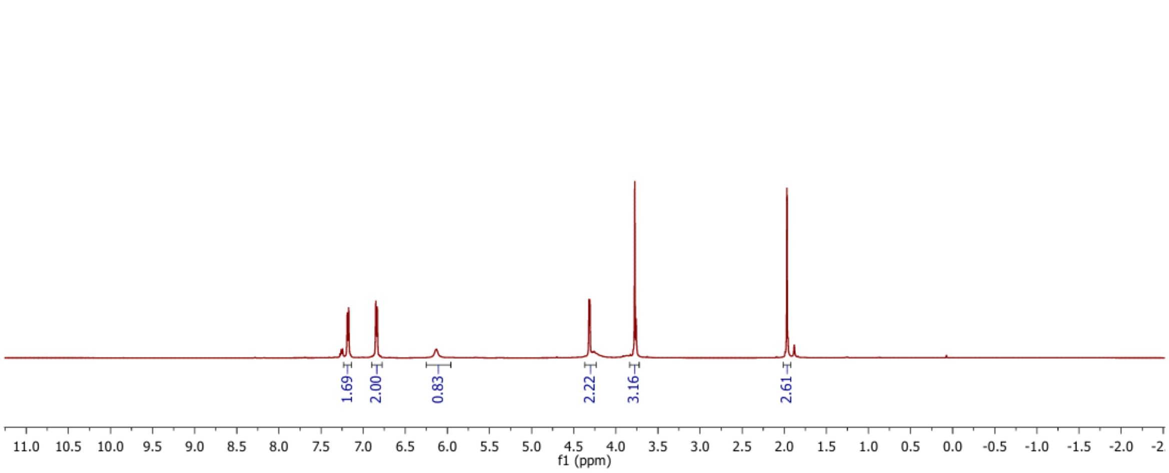

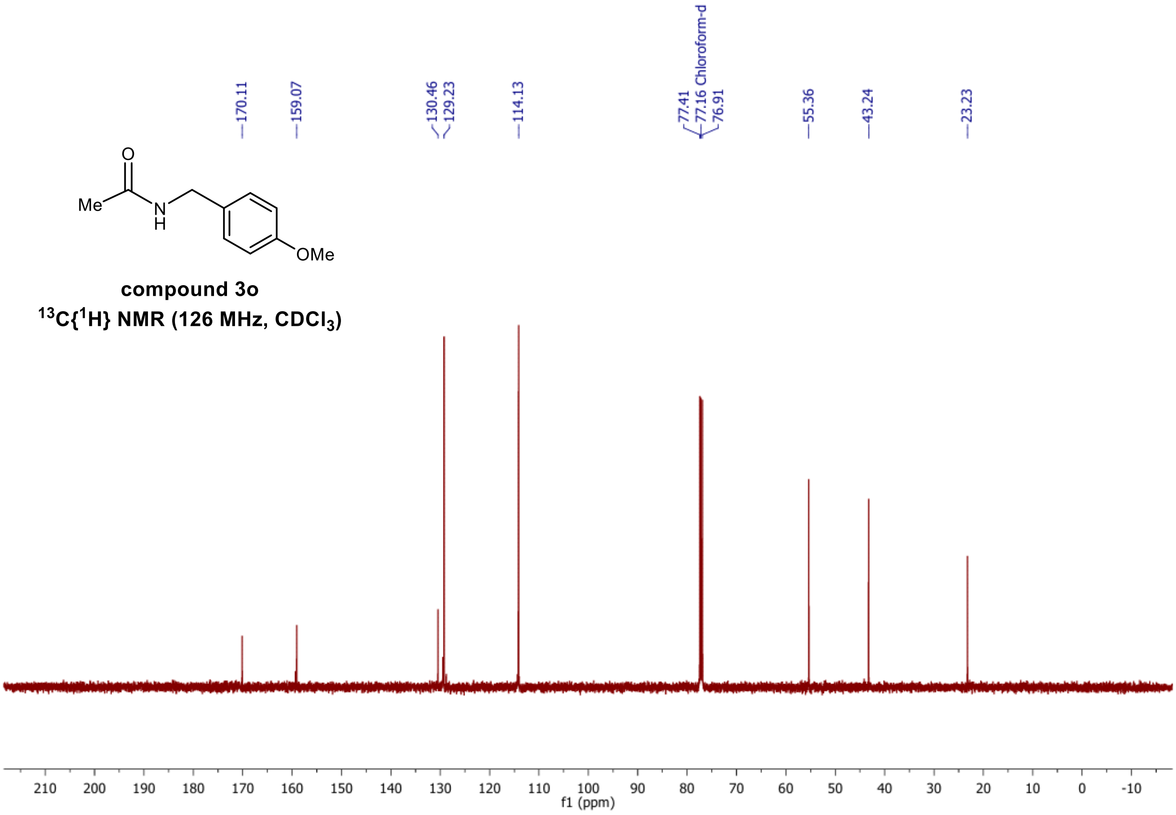

compound 3o
$^{13}C\{^{1}H\}$ NMR (126 MHz, $CDCl_3$)

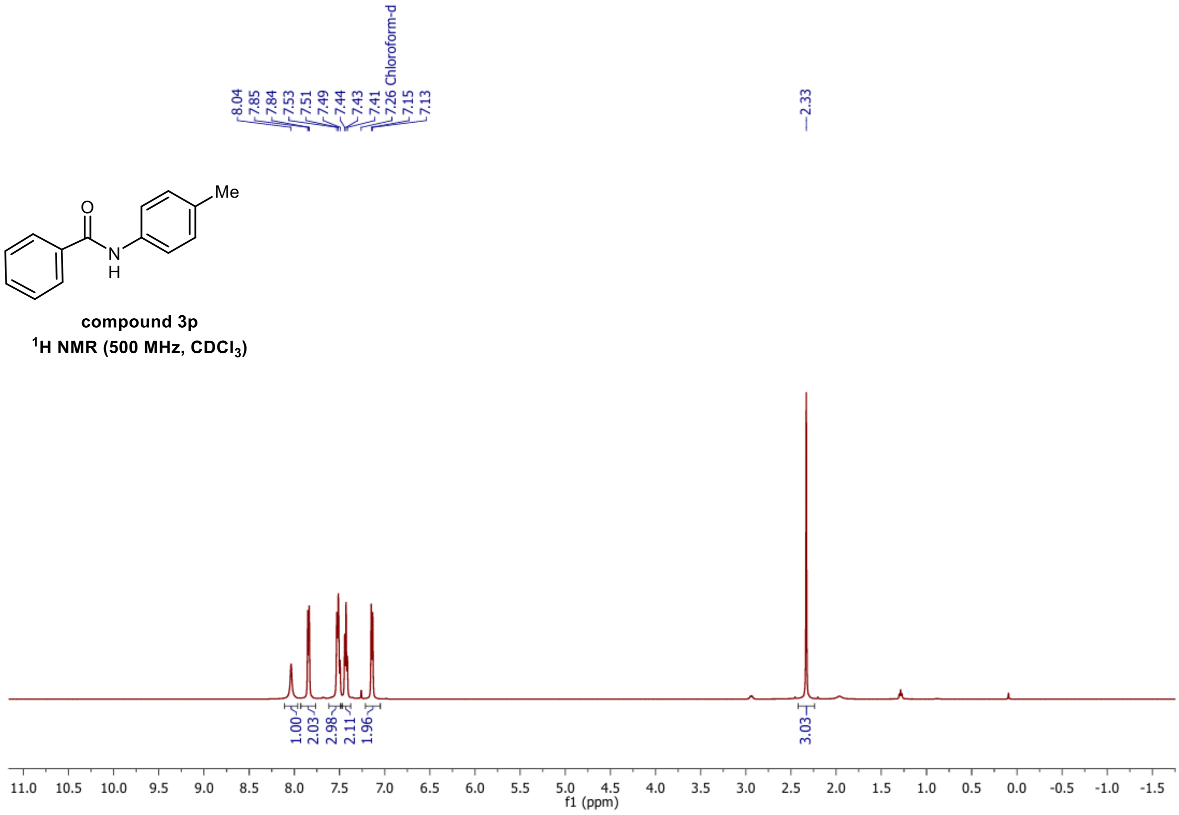

compound 3p
^{1}H NMR (500 MHz, $CDCl_3$)

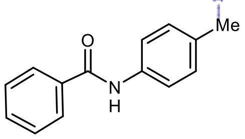

compound 3p

$^{13}C\{^{1}H\}$ NMR (126 MHz, $CDCl_3$)

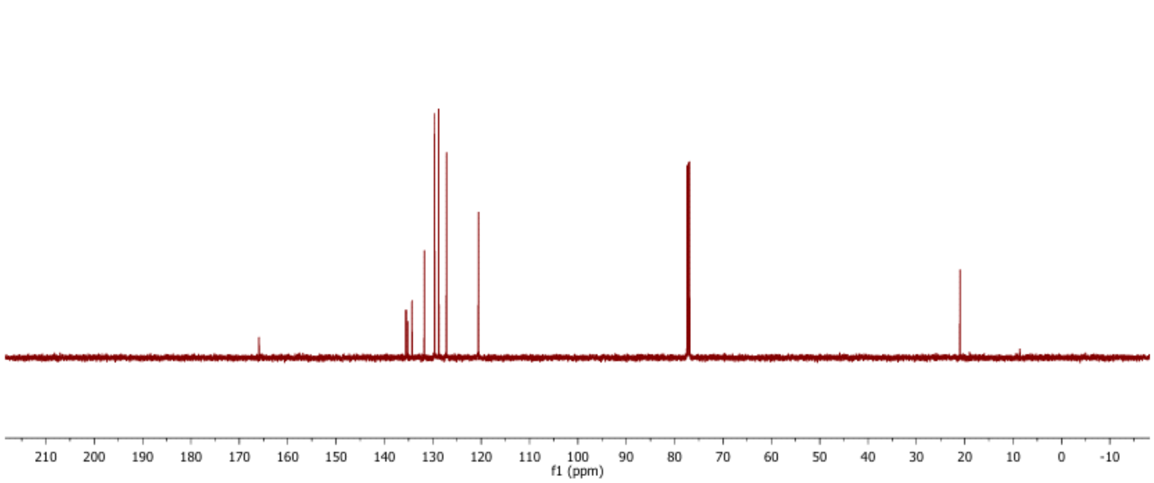

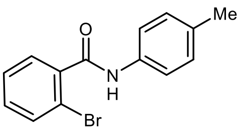

compound 3q

^{1}H NMR (500 MHz, $CDCl_3$)

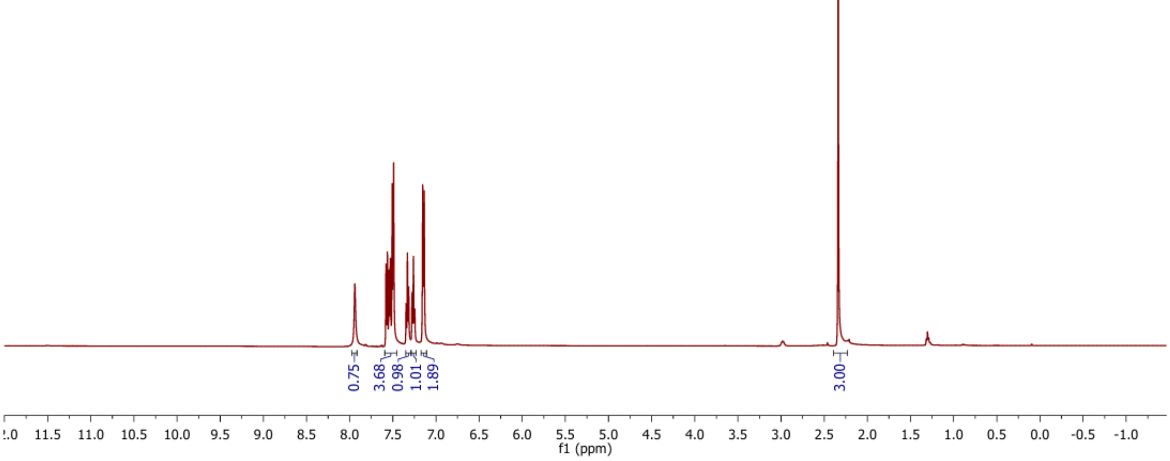

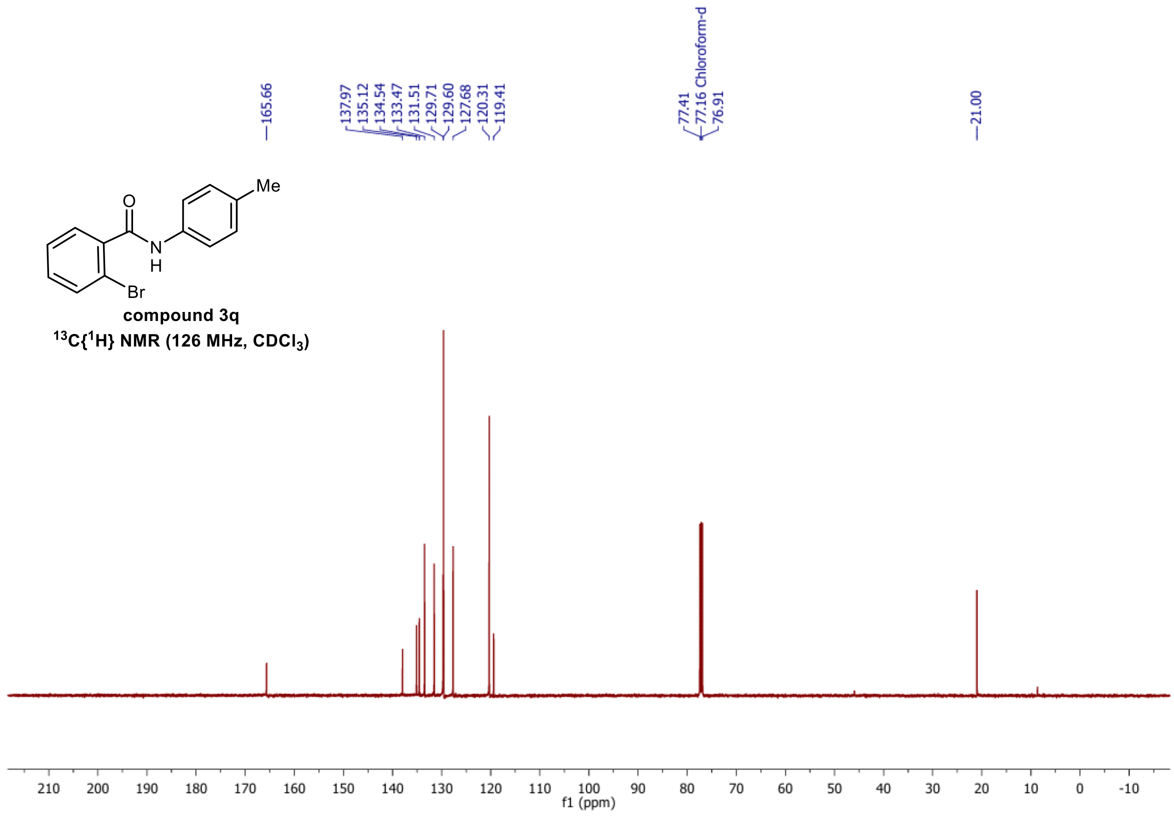
165.66
137.97
135.12
134.54
133.47
131.51
129.71
129.60
127.68
120.31
119.41
77.41
77.16 Chloroform-d
76.91
21.00
O
N
H
Me
Br
compound 3q
$^{13}C\{^{1}H\}$ NMR (126 MHz, $CDCl_3$)
f1 (ppm)

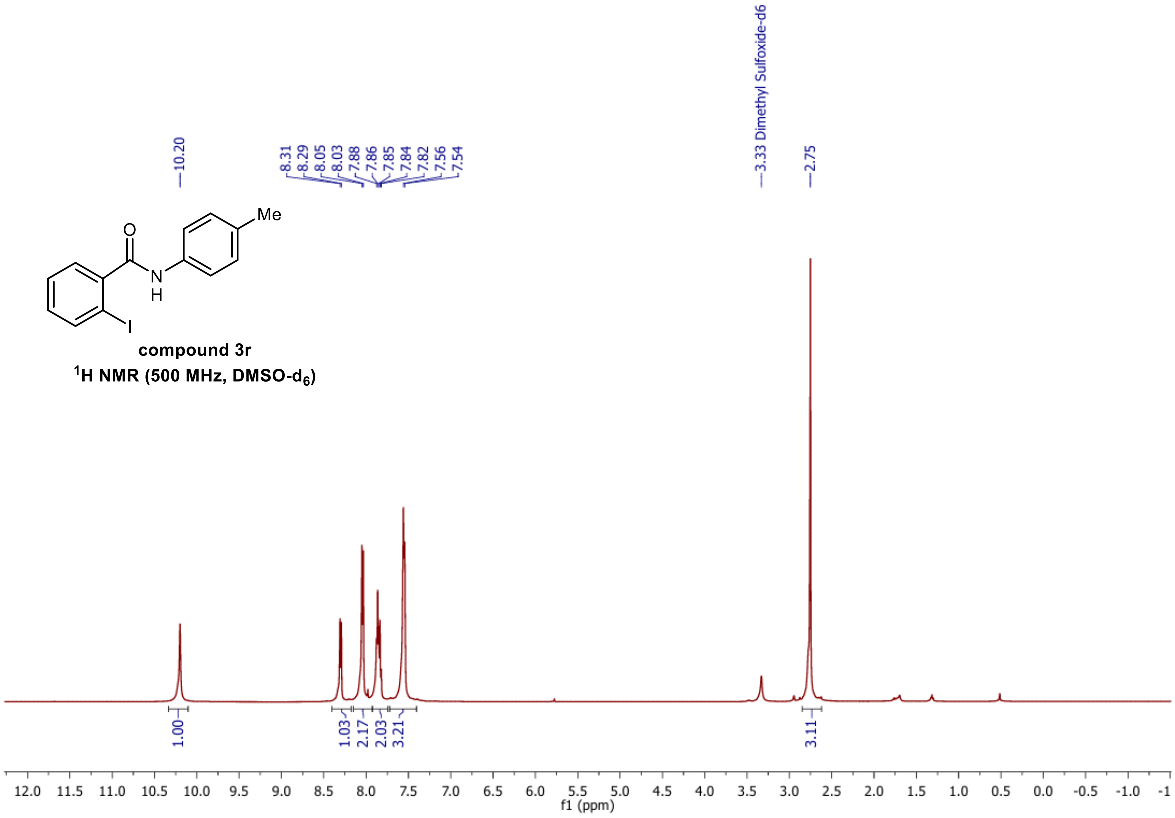
10.20
8.31
8.29
8.05
8.03
7.88
7.86
7.85
7.84
7.82
7.56
7.54
3.33 Dimethyl Sulfoxide-d6
2.75
O
N
H
Me
I
compound 3r
^{1}H NMR (500 MHz, DMSO-d_6)
1.00
1.03
2.17
2.03
3.21
3.11
f1 (ppm)

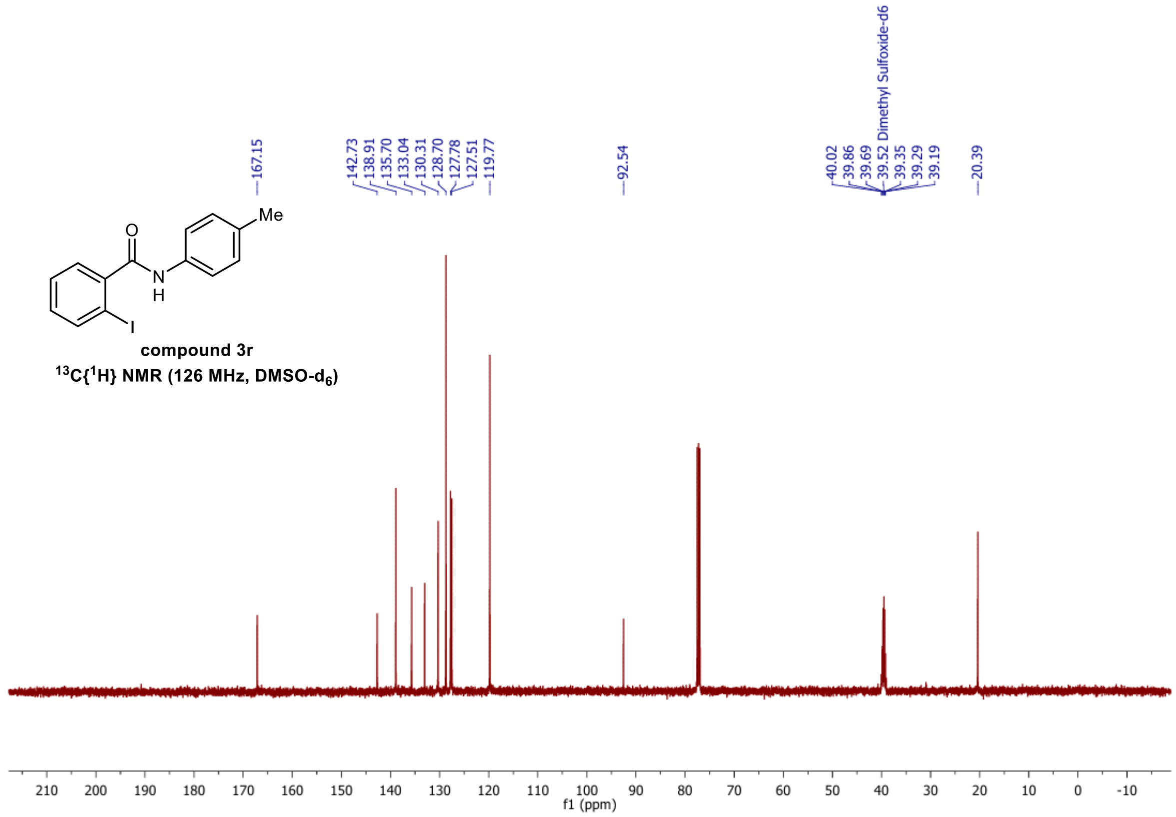
O
N
H
I
Me
compound 3r
13C{1H} NMR (126 MHz, DMSO-d6)
167.15
142.73
138.91
135.70
133.04
130.31
128.70
127.78
127.51
119.77
92.54
40.02
39.86
39.69
39.52 Dimethyl Sulfoxide-d6
39.35
39.29
39.19
20.39
f1 (ppm)

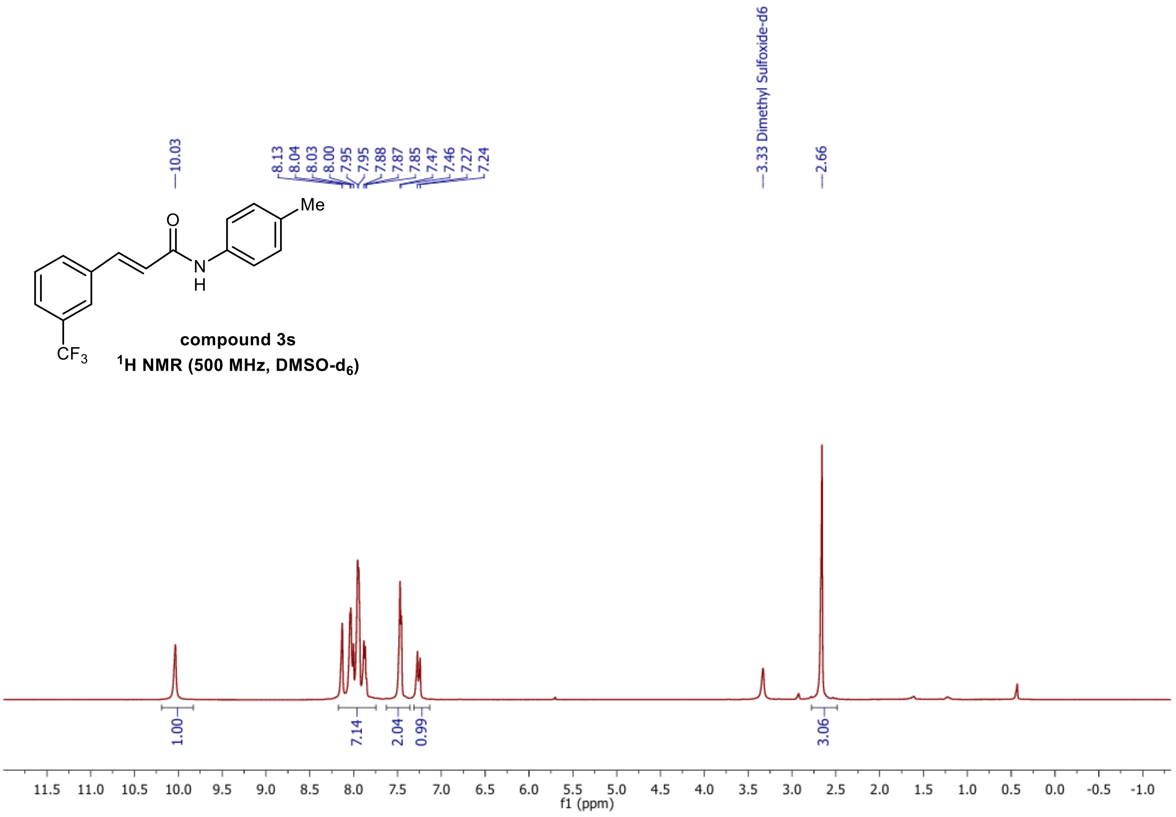
O
N
H
Me
CF3
compound 3s
1H NMR (500 MHz, DMSO-d6)
10.03
8.13
8.04
8.03
8.00
7.95
7.95
7.88
7.87
7.85
7.47
7.46
7.27
7.24
3.33 Dimethyl Sulfoxide-d6
2.66
1.00
7.14
2.04
0.99
3.06
f1 (ppm)

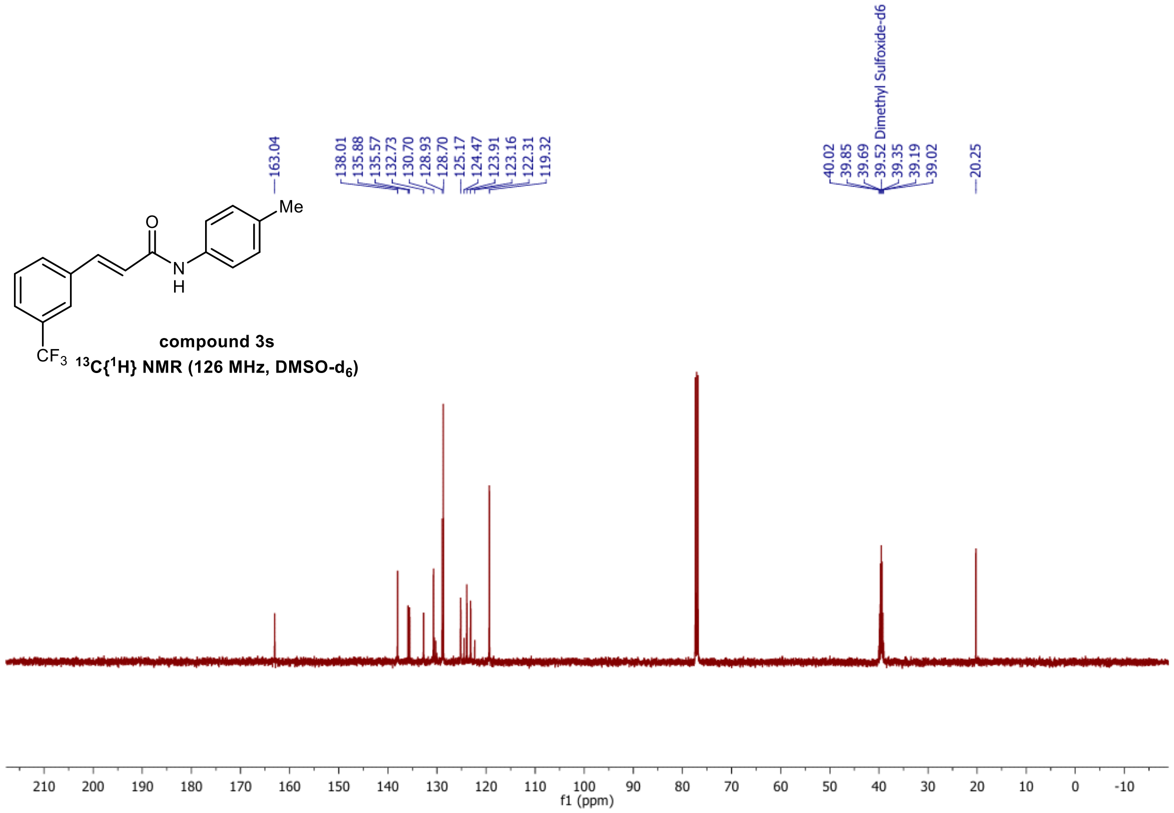
compound 3s
$^{13}C\{^1H\}$ NMR (126 MHz, DMSO-d_6)
O
N
H
Me
CF_3
163.04
138.01
135.88
135.57
132.73
130.70
128.93
128.70
125.17
124.47
123.91
123.16
122.31
119.32
40.02
39.85
39.69
39.52 Dimethyl Sulfoxide-d6
39.35
39.19
39.02
20.25
f1 (ppm)

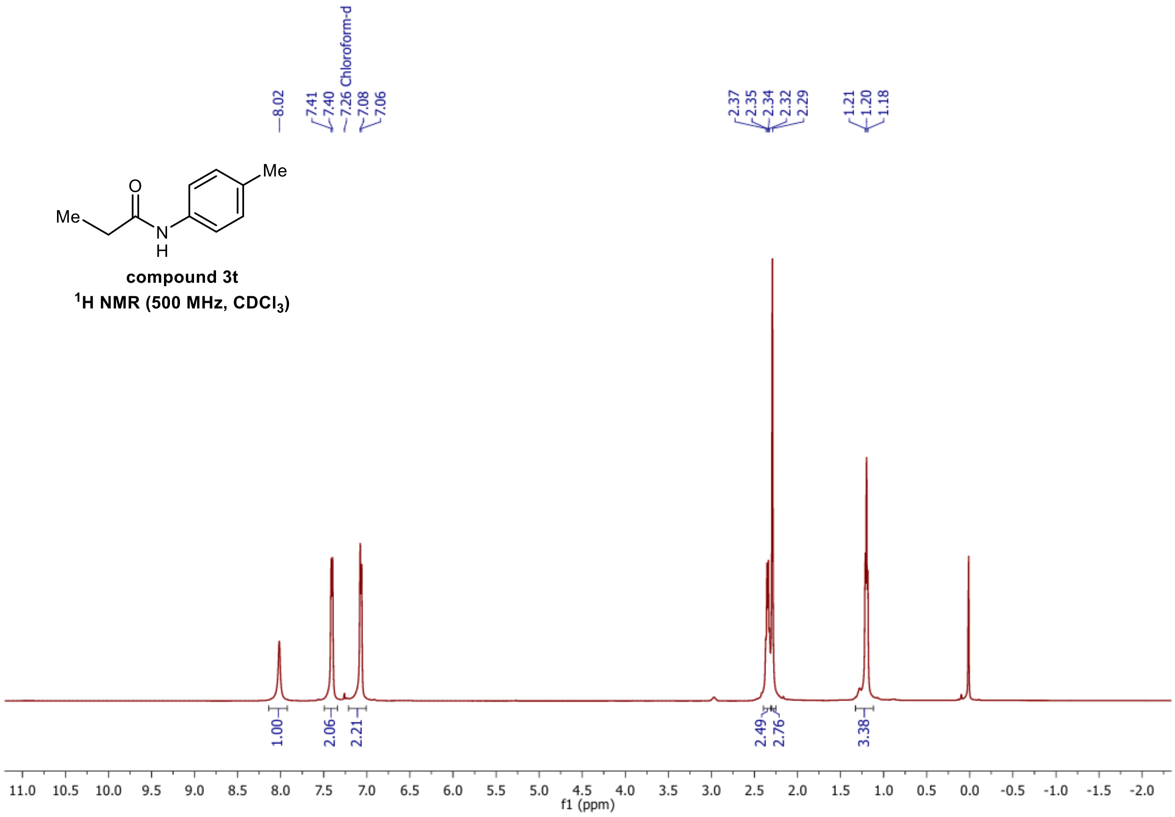
compound 3t
^1H NMR (500 MHz, $CDCl_3$)
Me
O
N
H
Me
8.02
7.41
7.40
7.26 Chloroform-d
7.08
7.06
2.37
2.35
2.34
2.32
2.29
1.21
1.20
1.18
1.00
2.06
2.21
2.49
2.76
3.38
f1 (ppm)

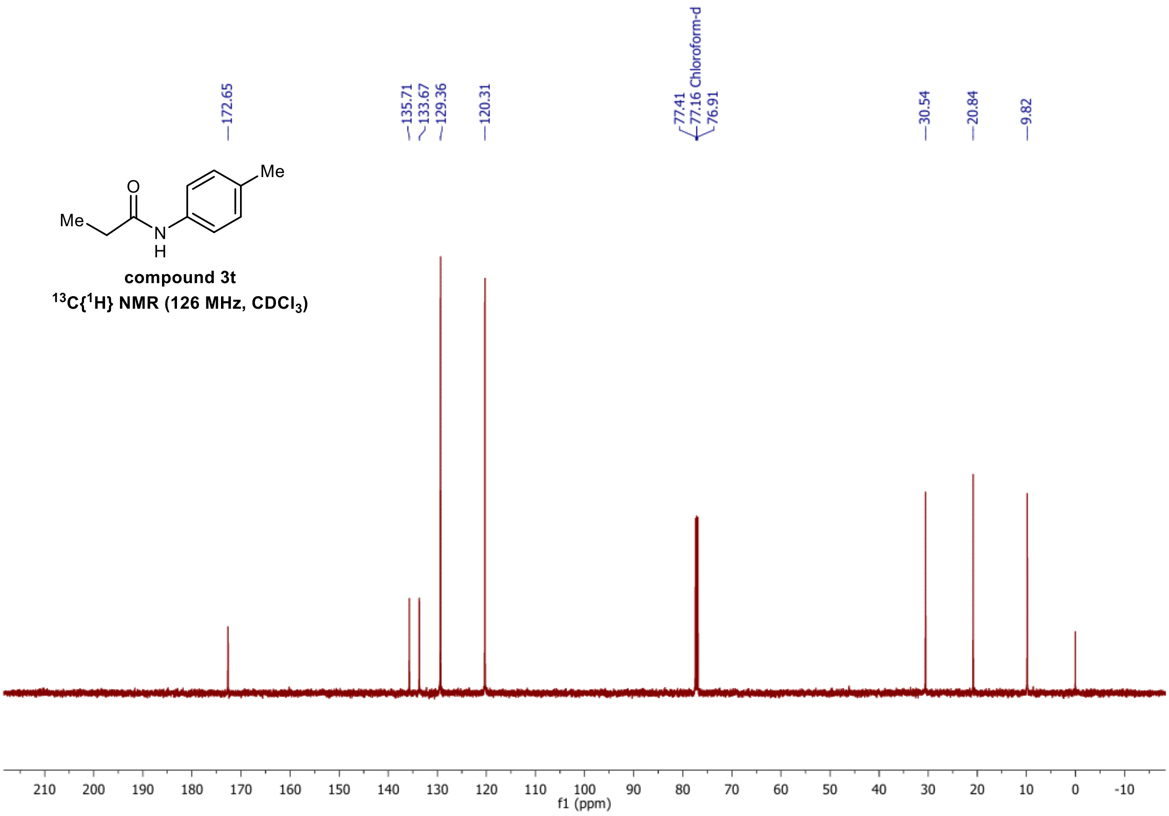
172.65
135.71
133.67
129.36
120.31
77.41
77.16 Chloroform-d
76.91
30.54
20.84
9.82
Me
O
Me
N
H
compound 3t
13C{1H} NMR (126 MHz, CDCl3)
f1 (ppm)

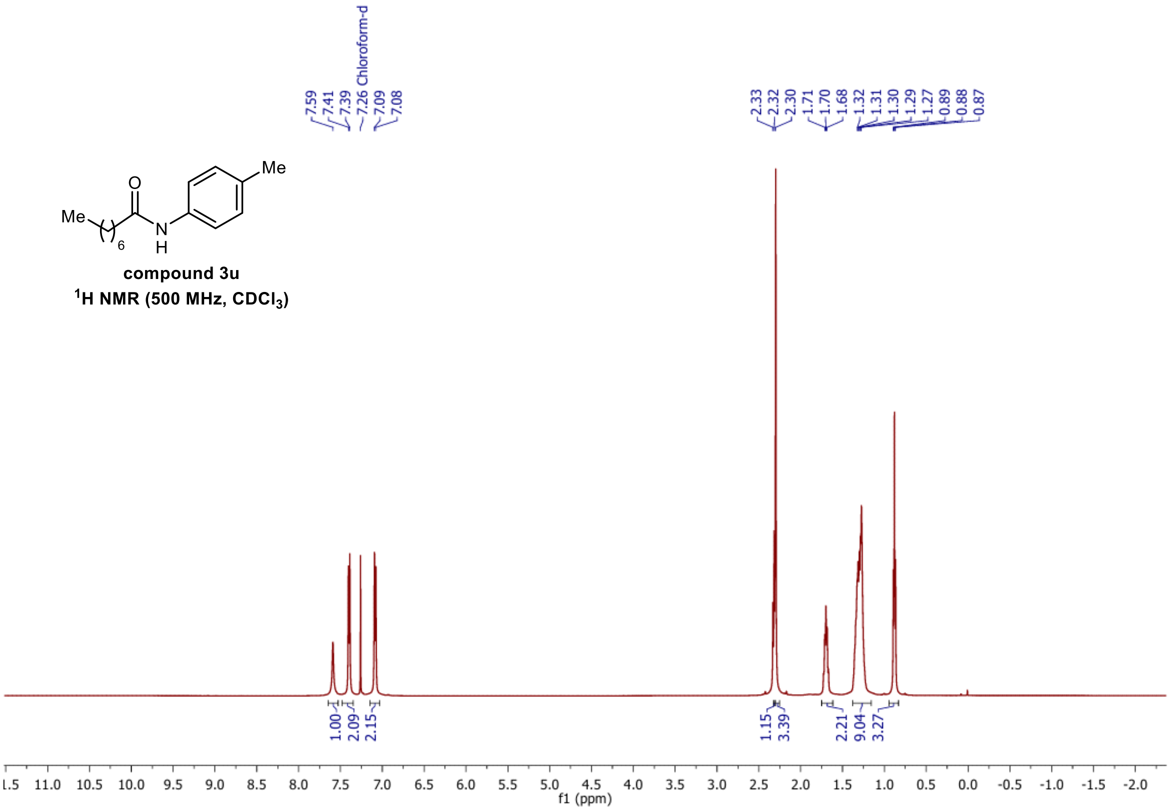
7.59
7.41
7.39
7.26 Chloroform-d
7.09
7.08
2.33
2.32
2.30
1.71
1.70
1.68
1.32
1.31
1.30
1.29
1.27
0.89
0.88
0.87
Me
O
Me
6
N
H
compound 3u
1H NMR (500 MHz, CDCl3)
1.00
2.09
2.15
1.15
3.39
2.21
9.04
3.27
f1 (ppm)

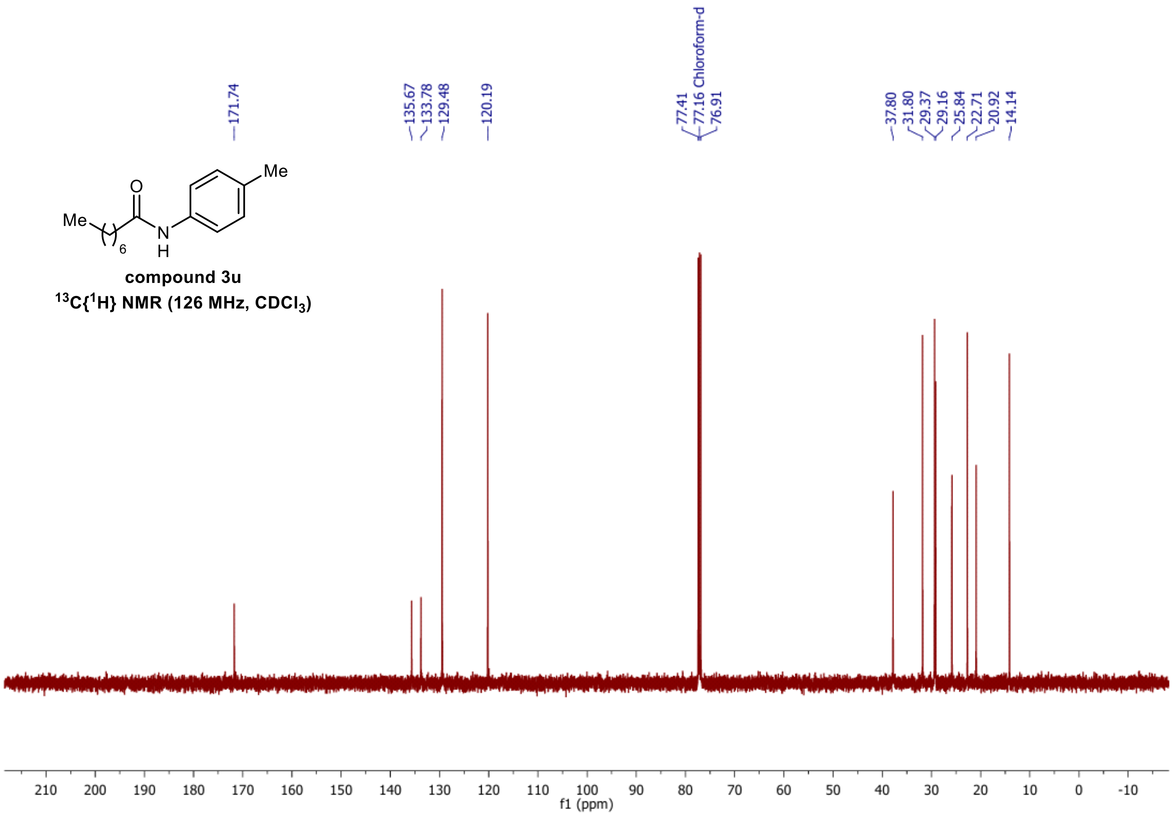

171.74
135.67
133.78
129.48
120.19
77.41
77.16 Chloroform-d
76.91
37.80
31.80
29.37
29.16
25.84
22.71
20.92
14.14
Me
O
N
H
6
compound 3u
$^{13}C\{^{1}H\}$ NMR (126 MHz, $CDCl_3$)
210 200 190 180 170 160 150 140 130 120 110 100 90 80 70 60 50 40 30 20 10 0 -10
f1 (ppm)

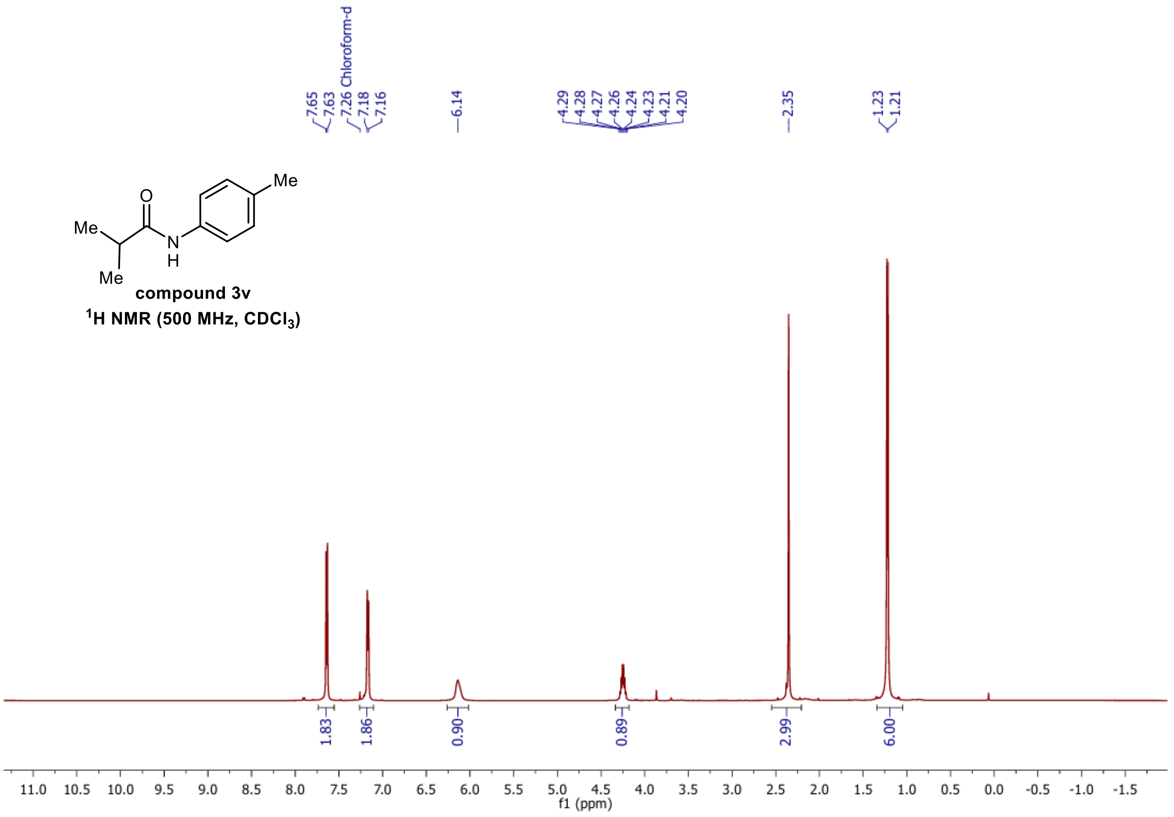

7.65
7.63
7.26 Chloroform-d
7.18
7.16
6.14
4.29
4.28
4.27
4.26
4.24
4.23
4.21
4.20
2.35
1.23
1.21
Me
O
Me
N
H
Me
compound 3v
^{1}H NMR (500 MHz, $CDCl_3$)
1.83
1.86
0.90
0.89
2.99
6.00
11.0 10.5 10.0 9.5 9.0 8.5 8.0 7.5 7.0 6.5 6.0 5.5 5.0 4.5 4.0 3.5 3.0 2.5 2.0 1.5 1.0 0.5 0.0 -0.5 -1.0 -1.5
f1 (ppm)

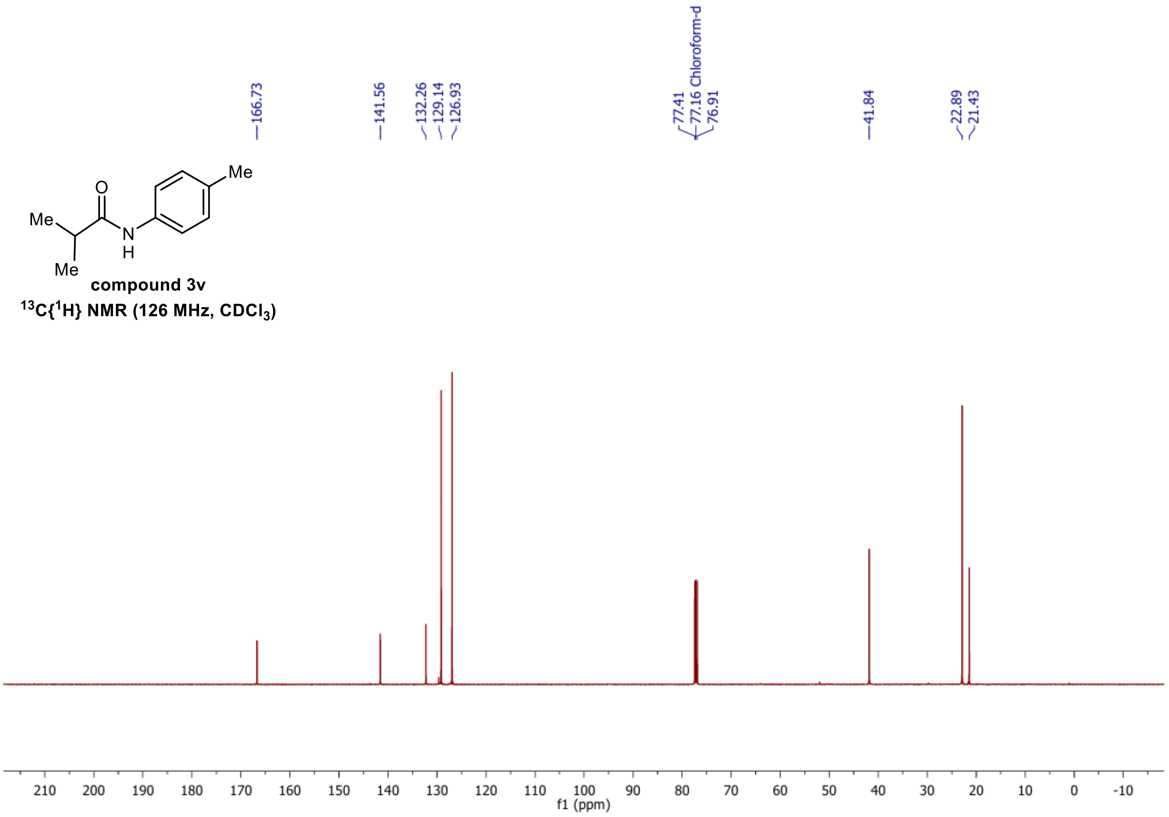
166.73
141.56
132.26
129.14
126.93
77.41
77.16 Chloroform-d
76.91
41.84
22.89
21.43
Me
O
Me
N
H
Me
compound 3v
13C{1H} NMR (126 MHz, CDCl3)
210 200 190 180 170 160 150 140 130 120 110 100 90 80 70 60 50 40 30 20 10 0 -10
f1 (ppm)

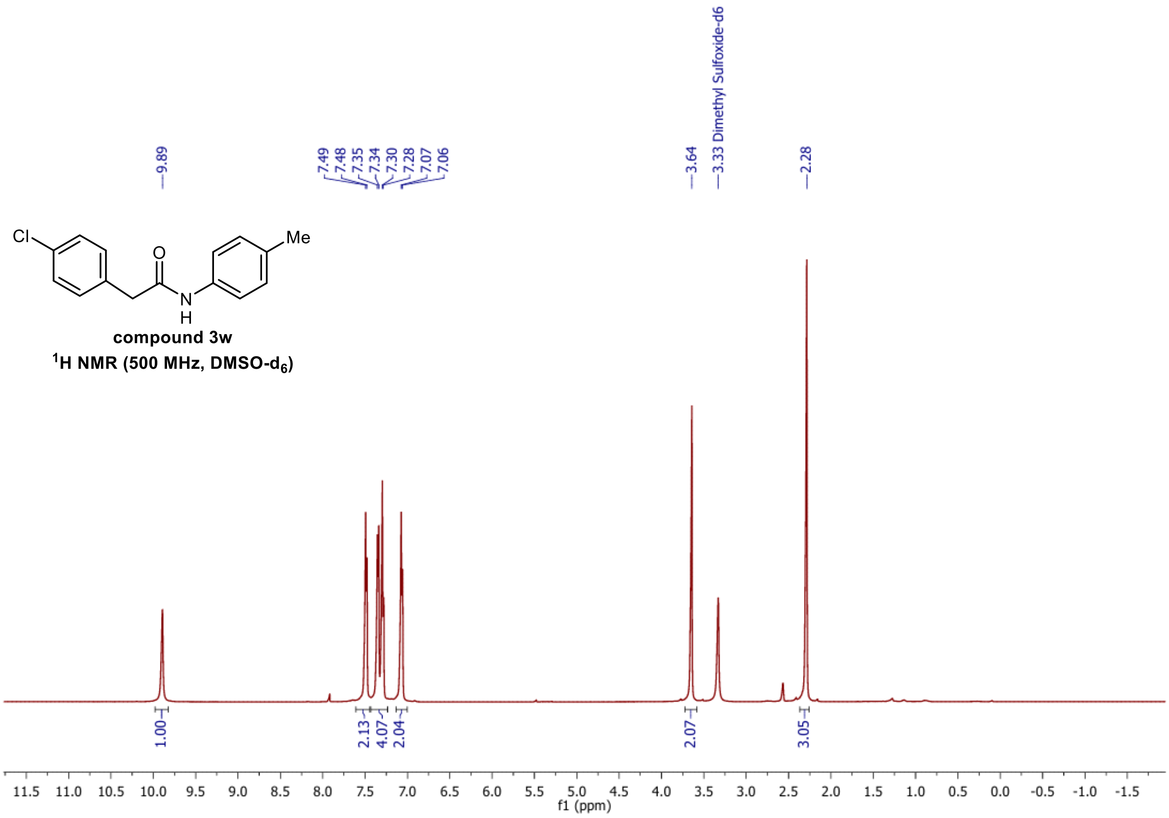
9.89
7.49
7.48
7.35
7.34
7.30
7.28
7.07
7.06
3.64
3.33 Dimethyl Sulfoxide-d6
2.28
Cl
Me
O
N
H
compound 3w
1H NMR (500 MHz, DMSO-d6)
1.00
2.13
4.07
2.04
2.07
3.05
11.5 11.0 10.5 10.0 9.5 9.0 8.5 8.0 7.5 7.0 6.5 6.0 5.5 5.0 4.5 4.0 3.5 3.0 2.5 2.0 1.5 1.0 0.5 0.0 -0.5 -1.0 -1.5
f1 (ppm)

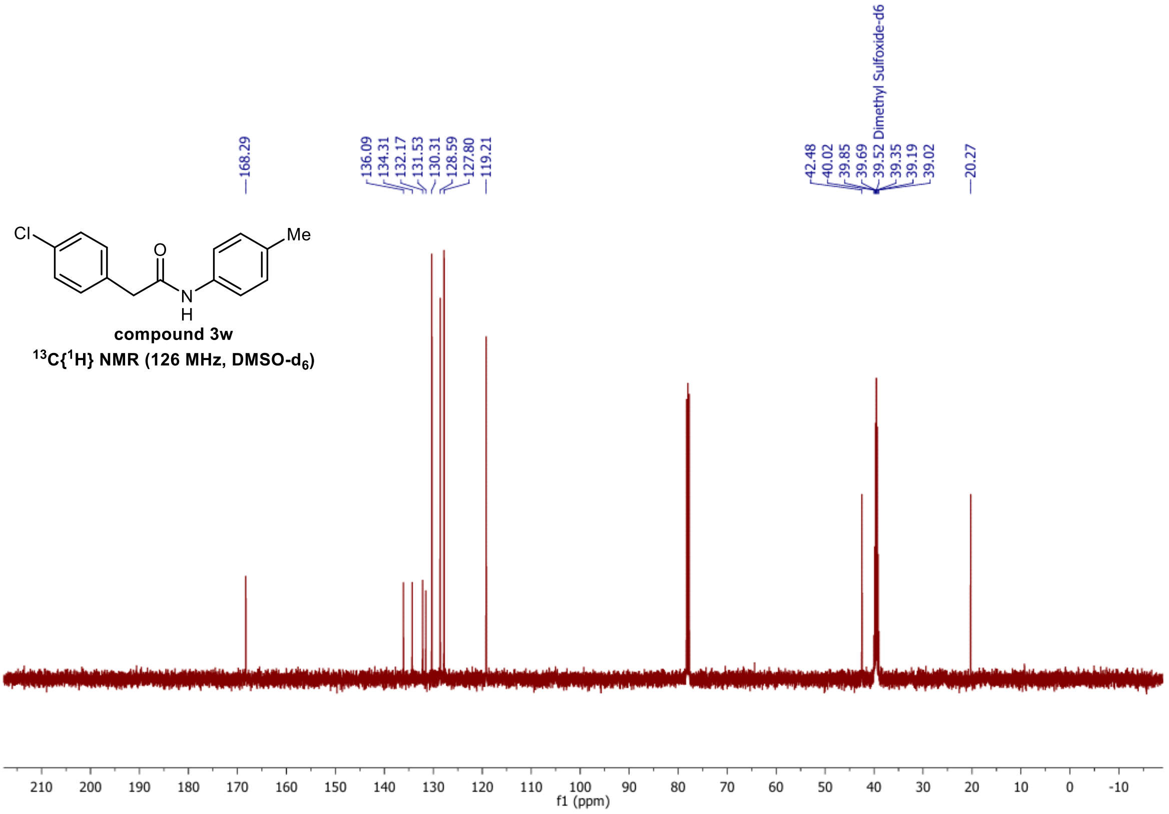
compound 3w
$^{13}C\{^{1}H\}$ NMR (126 MHz, DMSO-d_6)
168.29
136.09
134.31
132.17
131.53
130.31
128.59
127.80
119.21
42.48
40.02
39.85
39.69
39.52 Dimethyl Sulfoxide-d6
39.35
39.19
39.02
20.27
f1 (ppm)

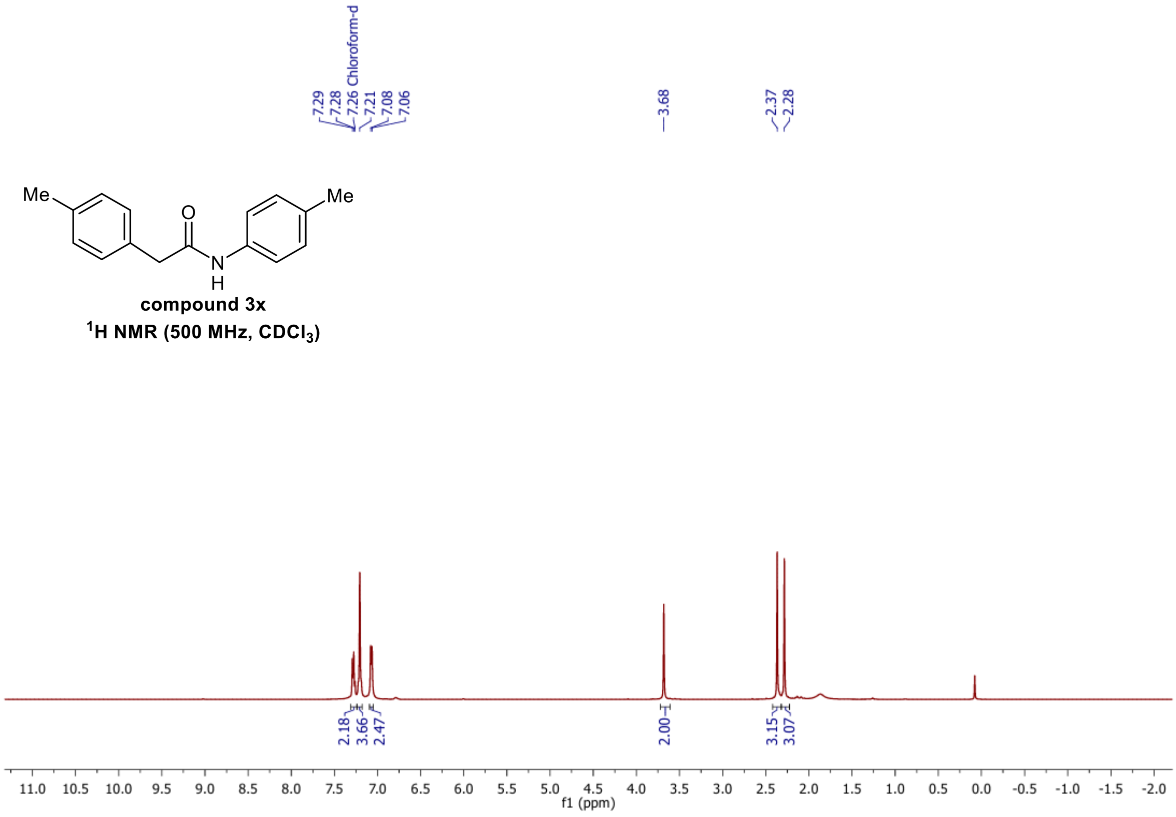
compound 3x
^{1}H NMR (500 MHz, $CDCl_3$)
7.29
7.28
7.26 Chloroform-d
7.21
7.08
7.06
3.68
2.37
2.28
2.18
3.66
2.47
2.00
3.15
3.07
f1 (ppm)

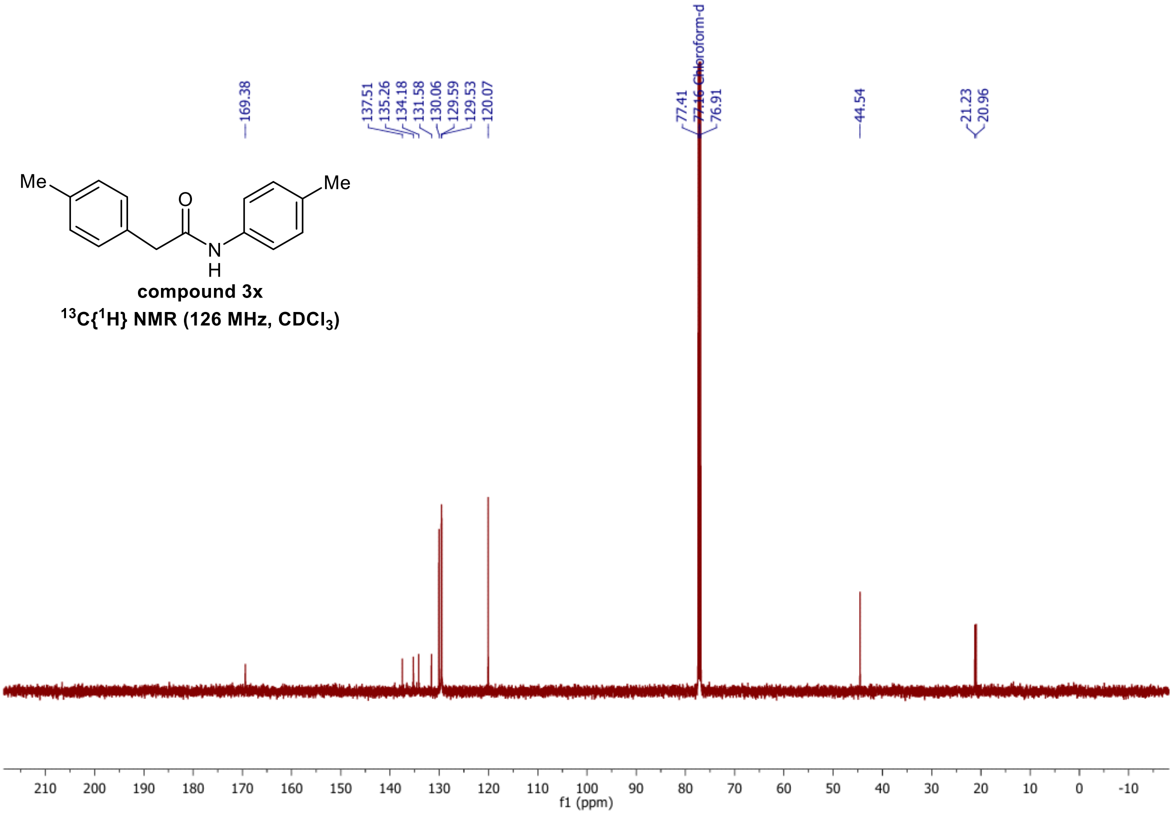

compound 3x

$^{13}C\{^{1}H\}$ NMR (126 MHz, $CDCl_3$)

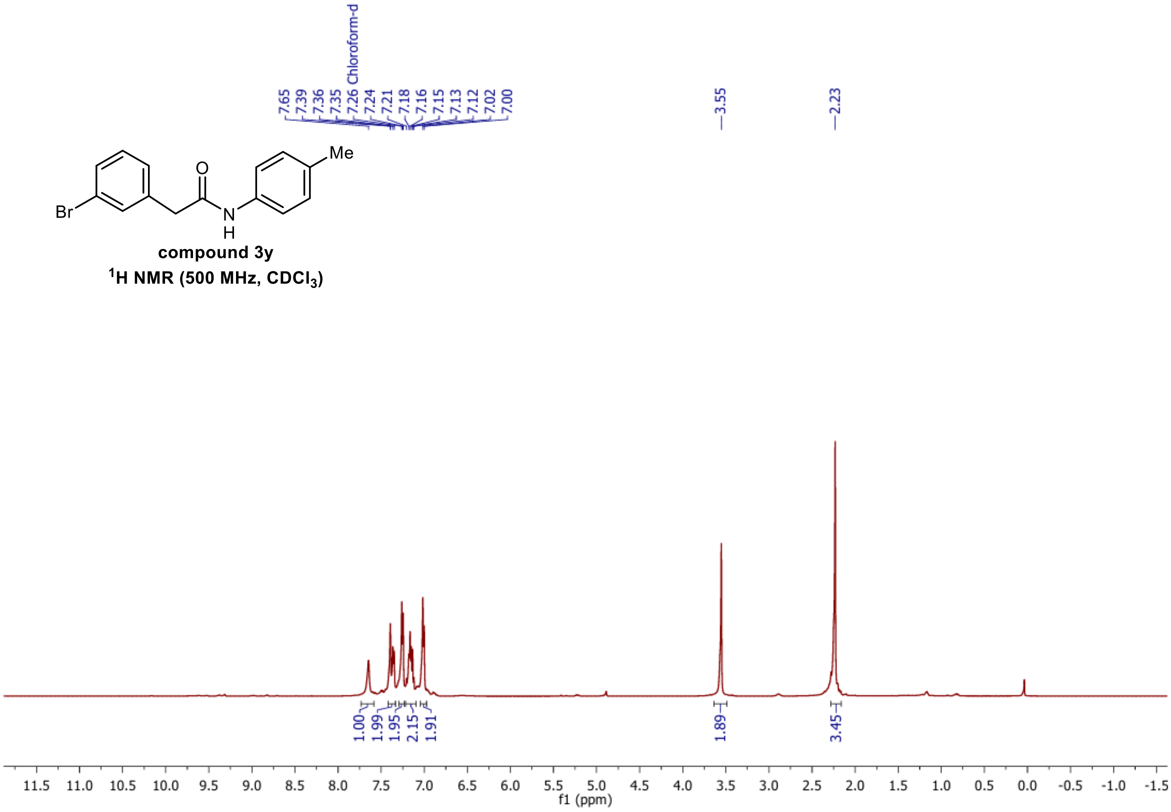

compound 3y

^{1}H NMR (500 MHz, $CDCl_3$)

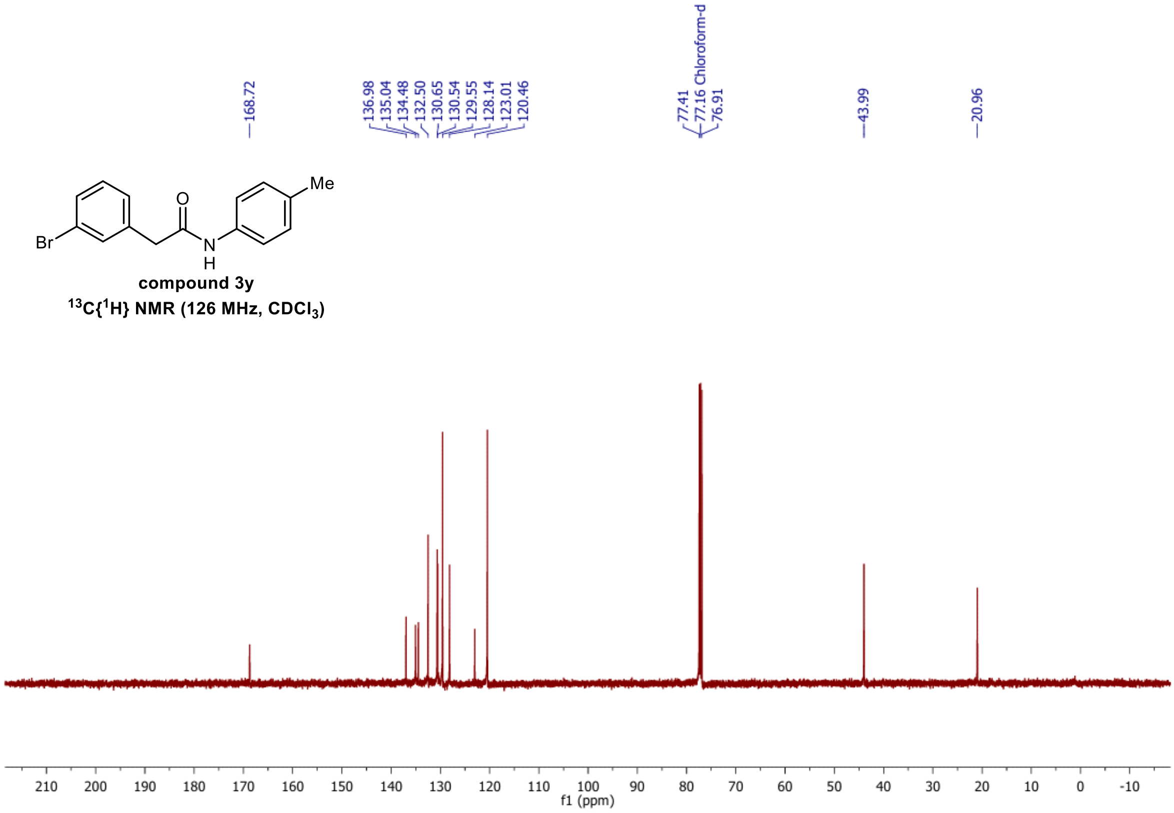
168.72
136.98
135.04
134.48
132.50
130.65
130.54
129.55
128.14
123.01
120.46
77.41
77.16 Chloroform-d
76.91
43.99
20.96
Br
O
N
H
Me
compound 3y
13C{1H} NMR (126 MHz, CDCl3)
f1 (ppm)

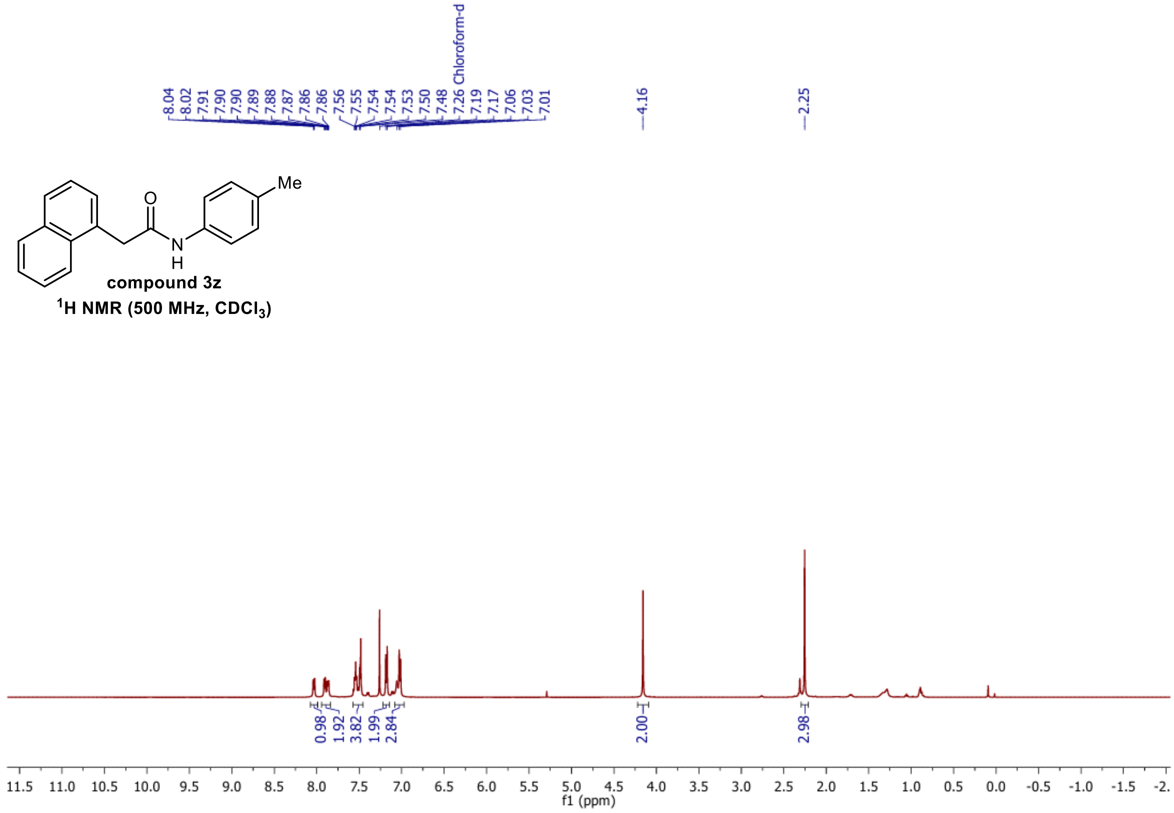
8.04
8.02
7.91
7.90
7.90
7.89
7.88
7.87
7.86
7.86
7.56
7.55
7.54
7.54
7.53
7.50
7.48
7.26 Chloroform-d
7.19
7.17
7.06
7.03
7.01
4.16
2.25
O
N
H
Me
compound 3z
1H NMR (500 MHz, CDCl3)
0.98
1.92
3.82
1.99
2.84
2.00
2.98
f1 (ppm)

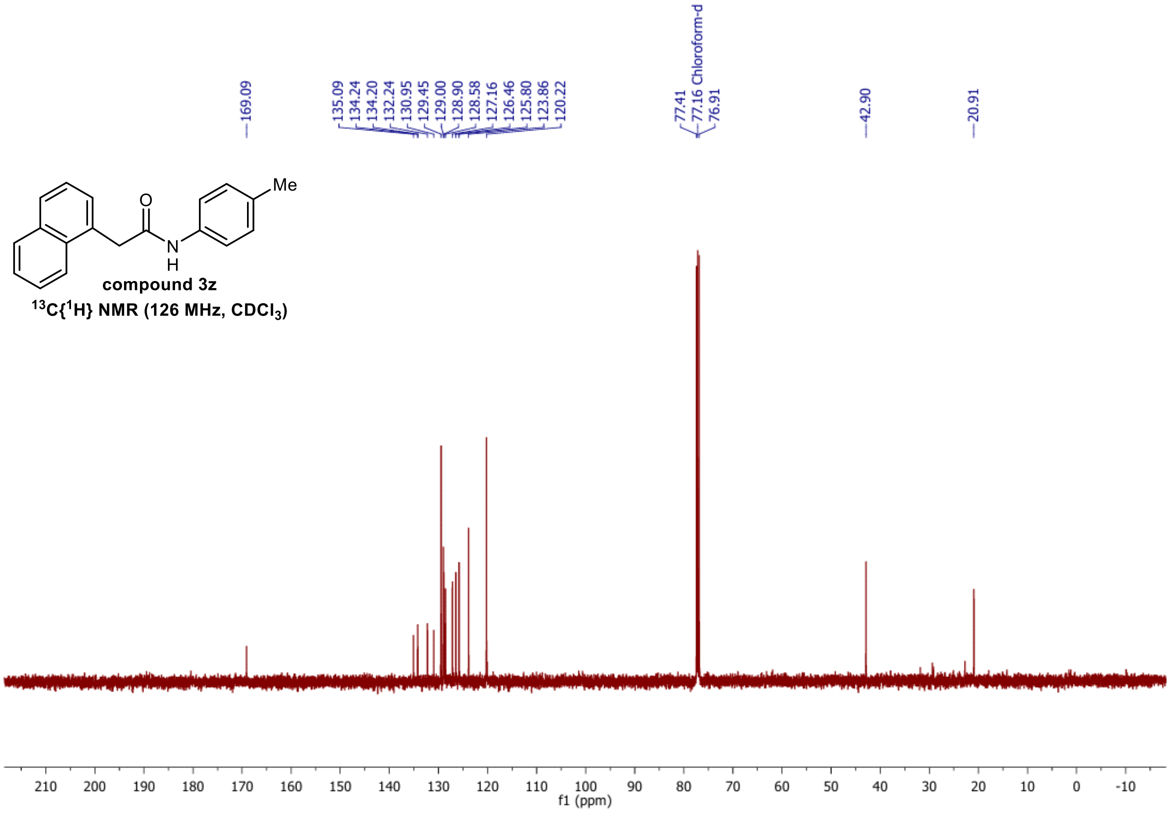

compound 3z
$^{13}C\{^{1}H\}$ NMR (126 MHz, CDCl$_3$)
169.09
135.09
134.24
134.20
132.24
130.95
129.45
129.00
128.90
128.58
127.16
126.46
125.80
123.86
120.22
77.41
77.16 Chloroform-d
76.91
42.90
20.91
f1 (ppm)

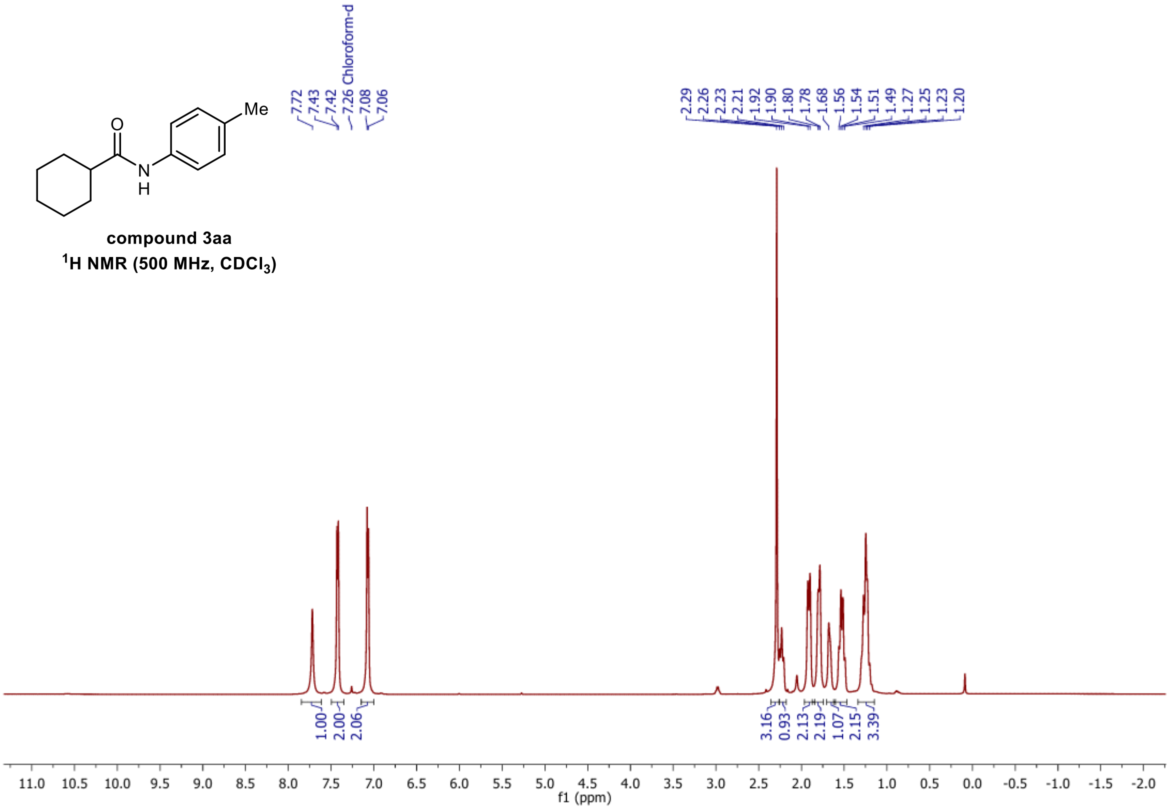

compound 3aa
^{1}H NMR (500 MHz, CDCl$_3$)
7.72
7.43
7.42
7.26 Chloroform-d
7.08
7.06
2.29
2.26
2.23
2.21
1.92
1.90
1.80
1.78
1.68
1.56
1.54
1.51
1.49
1.27
1.25
1.23
1.20
1.00
2.00
2.06
3.16
0.93
2.13
2.19
1.07
2.15
3.39
f1 (ppm)

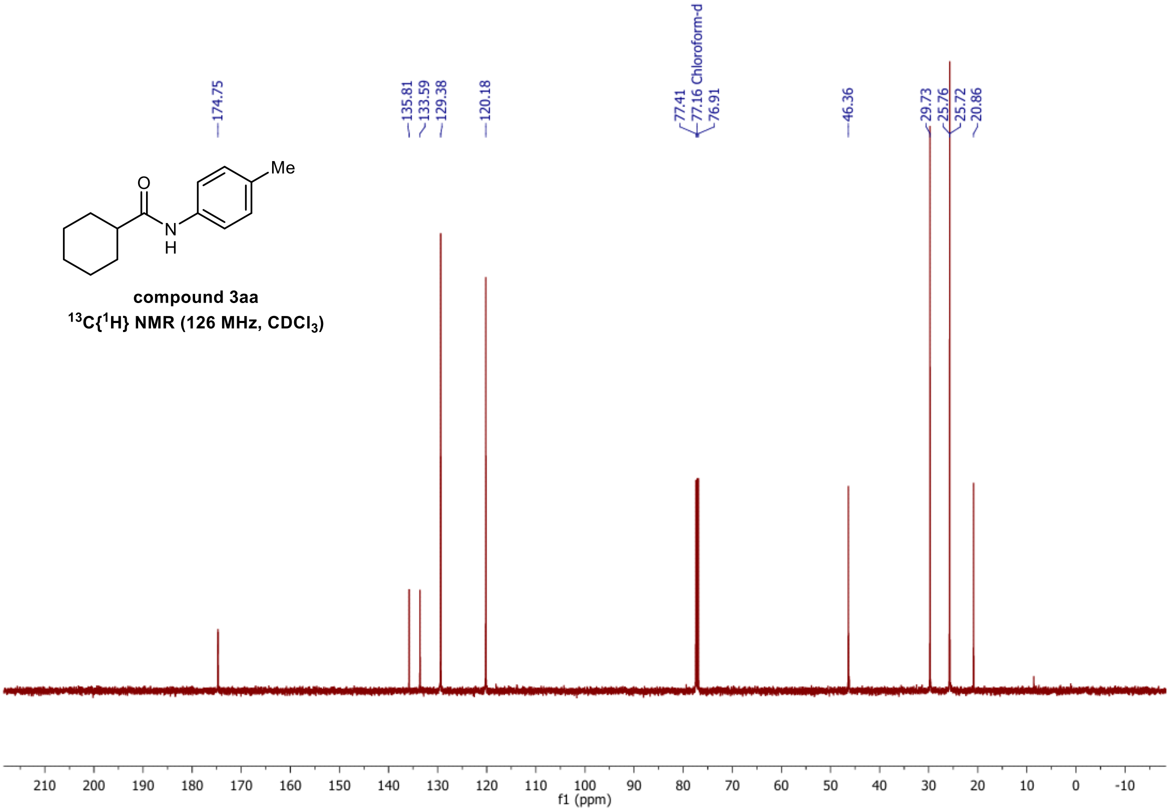
174.75
135.81
133.59
129.38
120.18
77.41
77.16 Chloroform-d
76.91
46.36
29.73
25.76
25.72
20.86
O
N
H
Me
compound 3aa
13C{1H} NMR (126 MHz, CDCl3)
210 200 190 180 170 160 150 140 130 120 110 100 90 80 70 60 50 40 30 20 10 0 -10
f1 (ppm)

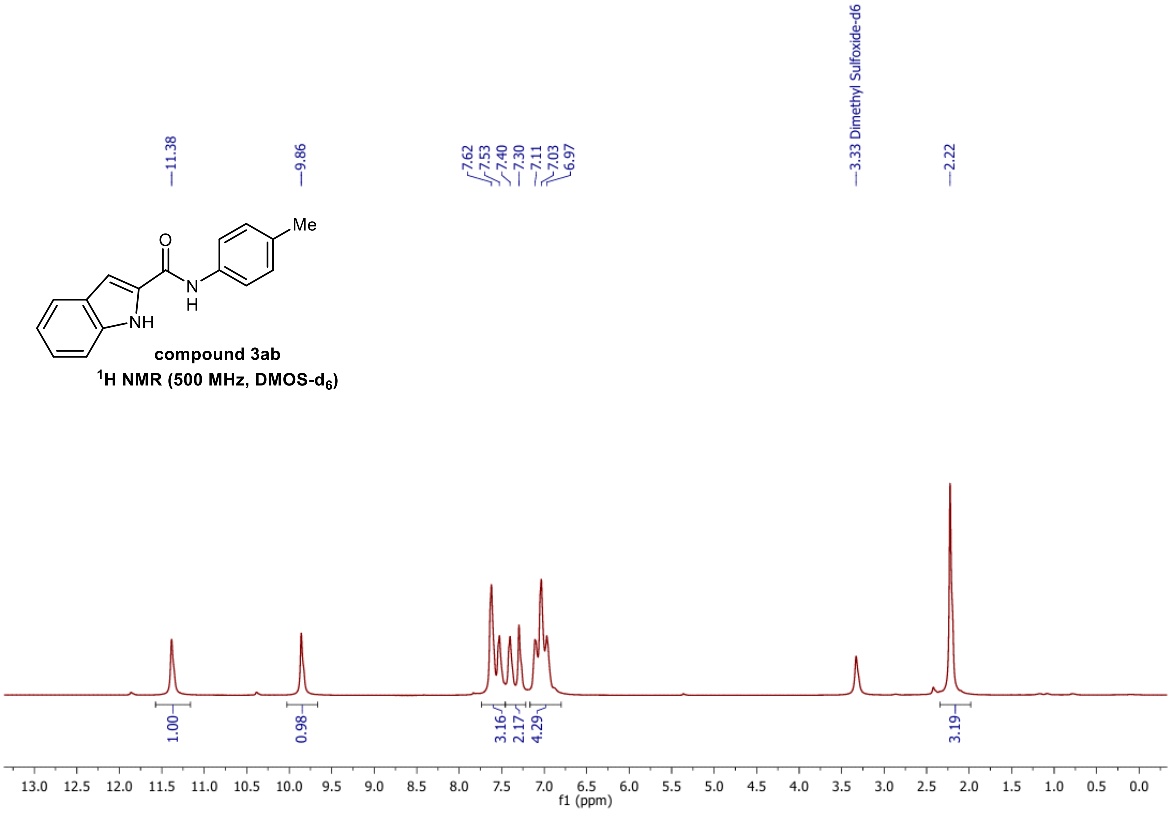
11.38
9.86
7.62
7.53
7.40
7.30
7.11
7.03
6.97
3.33 Dimethyl Sulfoxide-d6
2.22
O
N
H
NH
Me
compound 3ab
1H NMR (500 MHz, DMOS-d6)
1.00
0.98
3.16
2.17
4.29
3.19
13.0 12.5 12.0 11.5 11.0 10.5 10.0 9.5 9.0 8.5 8.0 7.5 7.0 6.5 6.0 5.5 5.0 4.5 4.0 3.5 3.0 2.5 2.0 1.5 1.0 0.5 0.0
f1 (ppm)

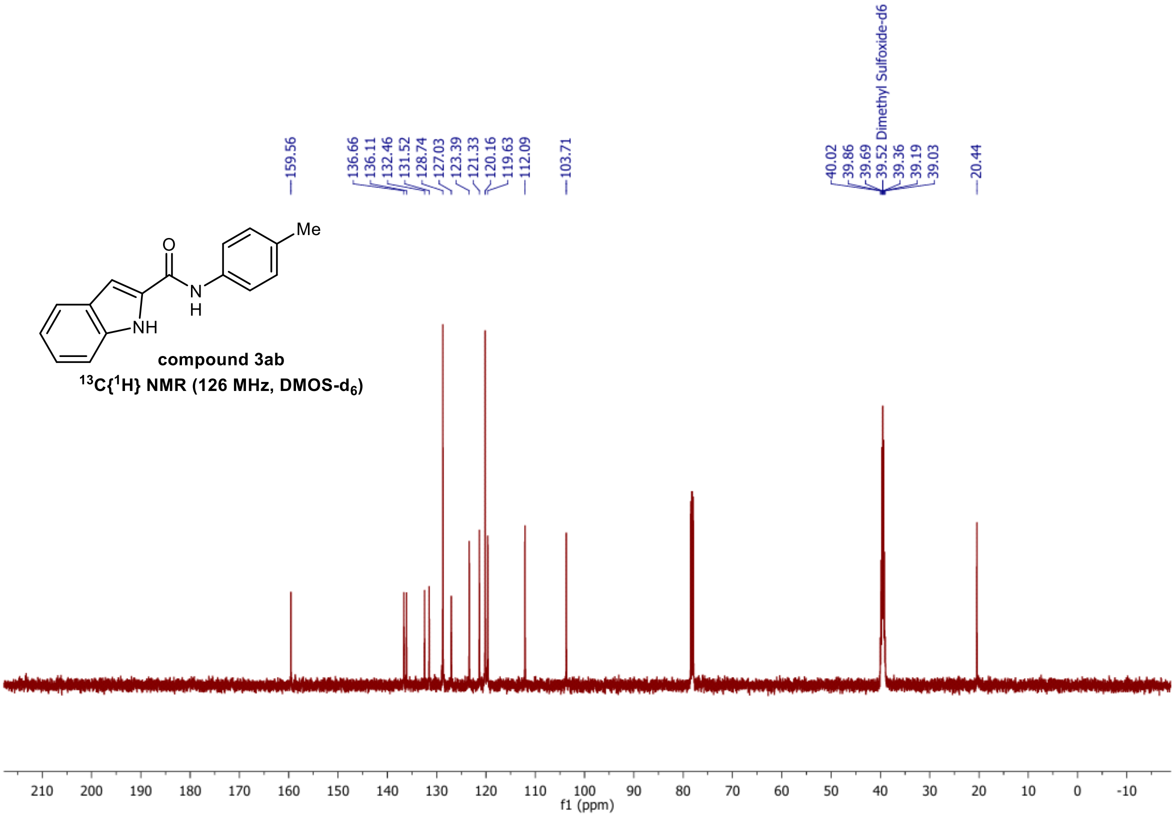
compound 3ab
$^{13}C\{^{1}H\}$ NMR (126 MHz, DMOS-d_6)
159.56
136.66
136.11
132.46
131.52
128.74
127.03
123.39
121.33
120.16
119.63
112.09
103.71
40.02
39.86
39.69
39.52 Dimethyl Sulfoxide-d6
39.36
39.19
39.03
20.44
f1 (ppm)

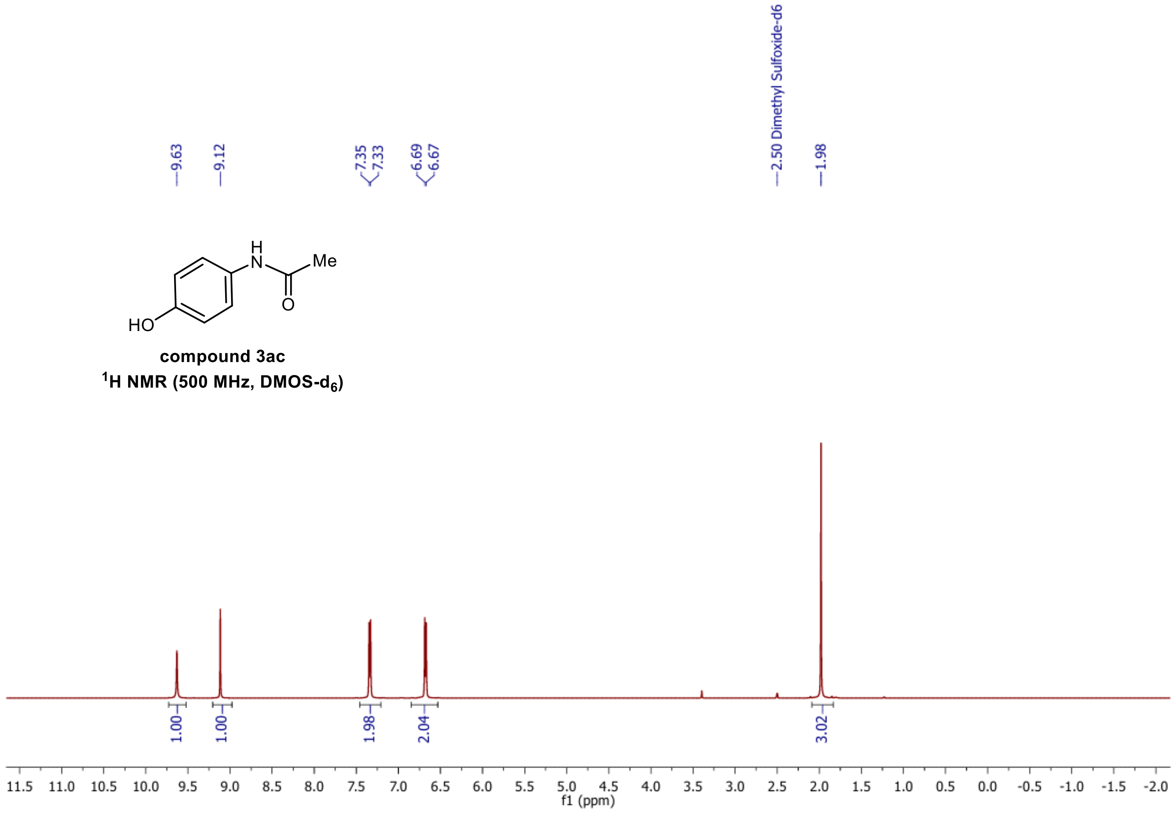
compound 3ac
^{1}H NMR (500 MHz, DMOS-d_6)
9.63
9.12
7.35
7.33
6.69
6.67
2.50 Dimethyl Sulfoxide-d6
1.98
1.00
1.00
1.98
2.04
3.02
f1 (ppm)

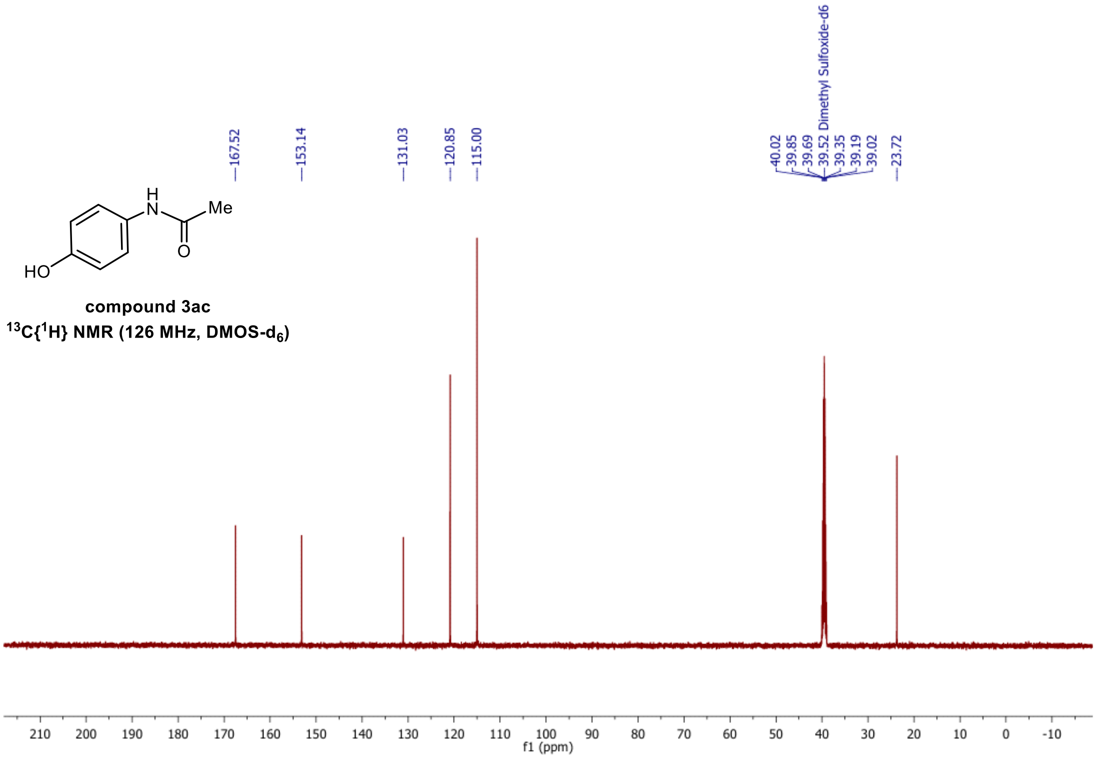
167.52
153.14
131.03
120.85
115.00
40.02
39.85
39.69
39.52 Dimethyl Sulfoxide-d6
39.35
39.19
39.02
23.72
HO
H
N
Me
O
compound 3ac
$^{13}C\{^{1}H\}$ NMR (126 MHz, DMOS-d_6)
210 200 190 180 170 160 150 140 130 120 110 100 90 80 70 60 50 40 30 20 10 0 -10
f1 (ppm)
